# Supplementary material for: Dual bronchodilators in Bronchiectasis study (DIBS): protocol for a pragmatic, multicentre, placebo-controlled, three-arm, double-blinded, randomised controlled trial studying bronchodilators in preventing exacerbations of bronchiectasis
Source: BMJ Open. 2023 Aug 10;13(8):e071906. doi: 10.1136/bmjopen-2023-071906 (PMC10423789; doi:10.1136/bmjopen-2023-071906)
Supplement: Supplementary data [file bmjopen-2023-071906supp001.pdf]

Newcastle University

TO BE PRINTED ON TRUST HEADED PAPER

Dual Bronchodilators in Bronchiectasis Study

DIBS

Participant Information Sheet

Version 4.0: 18th February 2022

INVITATION

We are inviting you to take part in our research study called DIBS. Please read the following information to help you decide if you want to take part. We would like you to understand why this research is being done and what it would involve for you. You don't have to decide straight away and you can talk to your friends and family or GP. A member of our team will go through this information sheet with you and answer any questions you may have. The study is being funded by the National Institute for Health Research (NIHR) which is the Health Research arm of the NHS.

TRIAL SUMMARY

- This study is looking at the use of bronchodilator inhalers for preventing exacerbations of bronchiectasis. Bronchodilators are a type of medication taken that relax the muscles in the lungs and widen the airways to make breathing easier. The inhalers being tested are a dual bronchodilator inhaler and a dual bronchodilator combined with corticosteroid inhaler (corticosteroid inhalers are also sometimes referred to as steroid inhalers). You will stop taking any inhaler(s) you are already on with the exception of your quick-acting inhaler (such as salbutamol). You will be randomised to either one of the two treatment inhalers or the placebo inhaler (a 'dummy' treatment). You will have four times the chance of

Morton M, et al. BMJ Open 2023; 13:e071906. doi: 10.1136/bmjopen-2023-071906

being on either one of the treatment inhalers as on the placebo inhaler. You and the study doctor will not know which inhaler you are taking.

- You will use your study inhaler once a day for 12 months (365 days).
- You will have 4 hospital visits for the study over the 12 months. These visits will take place alongside your normal hospital appointments or as additional visits. During these visits you will have some tests and complete some questionnaires.
- You will have a telephone call approximately every month to check that you have received your replacement inhaler through the post.
- You will be asked to complete an exacerbation diary each week for 12 months to record any bronchiectasis exacerbations.
- The study will include 600 bronchiectasis patients at over 20 hospitals.
- Your participation is voluntary, you will be part of a national effort to better understand bronchiectasis and its treatment.

Please read the following information for further details about the study if you are interested in taking part.

WHY IS DIBS NEEDED?

There are over 300,000 patients in the UK who suffer from bronchiectasis. There are currently no licensed treatments and no cures, which means there are large differences in care. Patients with bronchiectasis are normally treated with inhaled medicines approved for asthma and smoking-related chronic obstructive pulmonary disease (COPD) without any proof they work in bronchiectasis. We do not know which inhaled treatment is best for bronchiectasis; this is why we are conducting this research study. We will look at whether a dual bronchodilator inhaler or a dual bronchodilator plus a corticosteroid inhaler can reduce the number of bronchiectasis exacerbations (or flare ups) requiring antibiotic or steroid treatment compared to no treatment. An exacerbation (or flare up) is a sustained deterioration in your condition that would lead you to think about seeing your healthcare team or starting emergency packs.

Morton M, *et al. BMJ Open* 2023; 13:e071906. doi: 10.1136/bmjopen-2023-071906

The safety and side effects of these inhalers in other respiratory conditions are well known.

WHY AM I BEING INVITED TO TAKE PART?

You are being invited to take part in this research study because you have been diagnosed with a respiratory condition called bronchiectasis. You are also being invited because within the last 2 years you have had 2 or more exacerbations (or flare ups) within 12 months of each other which have required antibiotic or steroid treatment.

DO I HAVE TO TAKE PART?

No, it is up to you to decide whether you want to join the study. If you agree to take part, we will ask you to complete a consent form. If you choose not to, you will continue to get the standard care arranged by your doctor.

If you agree to take part, you can change your mind at any time. You can stop taking study medication and carry on with the questionnaires and study visits, or you can withdraw from the study completely. You do not have to give a reason, but it is helpful to the study if you do so ways it can be improved can be understood. If you decide to withdraw completely, data collected up to this point will be retained for analysis. This will not affect the care that you receive.

WHAT WILL GIVING CONSENT MEAN FOR ME?

By signing a consent form, this means that you fully understand what taking part in the study means for you. That's why it is really important that you take as much time as you want to read this information sheet and ask lots of questions.

WHAT WOULD TAKING PART INVOLVE?

We will ask you to attend 4 hospital visits:

- Initial visit
- 1 month follow-up visit
- 6 month follow-up visit
- 12 month follow-up visit

Morton M, et al. BMJ Open 2023; 13:e071906. doi: 10.1136/bmjopen-2023-071906

These visits are not part of your standard care but could take place alongside your standard care visits if it is possible. The diagram on page 66 shows the details of the study hospital visits and what will take place at each visit.

During your study hospital visits you will be asked to complete some questionnaires. Some will be completed by you and others will be completed by a member of the study team who will ask you questions. These questionnaires are used to assess any breathlessness you may have, the impact of your health on your usual activities, your mental health and quality of life. There are also questionnaires about how often you have had to access healthcare for routine appointments and urgent care, any costs you have incurred accessing healthcare and time taken to travel to access healthcare. We will use this to compare the overall healthcare costs between each treatment arm in the study.

A blood sample and urine sample (if needed for a pregnancy test) will be taken at the first (baseline) study visit. The blood sample is taken to look at reasons why patients may respond differently to the inhalers and as a baseline test for safety reasons. We will collect approximately 1 teaspoon (5 mL) full of blood. These samples will be analysed in the local hospital laboratory and will be destroyed after analysis. No samples (blood or urine) will be stored for the purposes of this study.

All of your study visits and procedures will be carried out in line with your study hospital’s COVID-19 policies and procedures. These will be explained to you when your appointments are arranged.

For the 12 months of the study you will be asked to complete an exacerbation diary each week. You will record any exacerbations of your bronchiectasis in the last week and any antibiotic or steroid treatment.

We will arrange delivery of your inhalers in between the face to face visits, at a time convenient for you. These will be delivered by post or courier, depending on your local research team’s policy.

You will have phone calls approximately every month with a member of the research team to check:

- That you have received your replacement inhaler in the post
- The current dose count on your inhaler

- Whether your contact details have changed
- Whether you have any planned travel/holidays that may affect the timing of the subsequent inhaler postings
- Completion of your exacerbation diary each week
- Whether you have had any adverse events (new or worsened signs or symptoms) or any hospital admissions

You will have to stop taking any current inhalers, with the exception if you have a blue (Salbutamol) inhaler used to relieve a sudden attack of breathlessness or wheezing. If you get this sort of attack you must continue to use a quick-acting inhaler (such as salbutamol).

You will have a phone call between 7 and 10 days after your last dose of the study inhaler. This call is to check whether you have had any adverse events (new or worsened signs or symptoms) or any hospital admissions since your most recent study visit.

We are interested in the long-term health of participants in this study. With your permission we will continue to ask for information relating to your health following the end of your participation in the study for up to one year (referred to in the study as 24 month follow up data), depending on the date you join the study. You will not need to visit hospital or be called by the research team for this. Instead, we will ask for information from Bronchiectasis Registries (BronchUK [The United Kingdom Bronchiectasis Registry] and EMBARC [European Multicentre Bronchiectasis Audit and Research Collaboration]) if you have already given consent to these projects for health follow ups. Or, we may ask for information from routinely collected sources such as NHS Digital (including Hospital Episodes Statistics [HES]) and the Office for National Statistics (ONS) (ONS/HES data will not be collected for participants with trial visits at Scottish hospitals). Members of the hospital research team may also review your medical records. The data collected will only include information about you for up to one year after your last dose of study medication, but, because data is requested in batches from HES, ONS and other databases, the request for your data may be made up to two years after your participation in the study has ended.

Figure 1. Hospital visit details

SCREENING/BASELINE VISIT – VISIT LENGTH 2-3 HOURS

- Written informed consent
- Eligibility for the trial confirmed
- Medical history, smoking history and medication review
- Urine pregnancy test (if needed)
- Blood tests
- Questionnaires
- Lung function tests
- Randomisation to treatment arm
- Receive first inhaler
- Test of inhaler technique

1 AND 6 MONTH FOLLOW UP – VISIT LENGTH 1-2 HOURS

- Check contact details
- Medication and smoking review
- Lung function tests
- Questionnaires
- Test of inhaler technique and used inhaler return
- Review of exacerbation diary and any adverse events including hospital visits

12 MONTH FOLLOW UP – VISIT LENGTH 1-2 HOURS

- Medication and smoking review
- Lung function tests
- Questionnaires
- Used inhaler return
- Review of exacerbation diary and any adverse events including hospital visits

IRAS ID: 275595 | DIBS Participant Information Sheet | V4.0 |18th February 2022

WHAT TREATMENT WOULD I BE ON?

The active treatments we are testing have already been tested and shown to work in the smoking related lung condition called COPD. The inhalers are already in wide use across the UK for that condition. If you decide to take part in the study, you will be randomised by computer to one of three treatment groups: each arm has ONLY one inhalation from one inhaler per day.

- Dual inhaler (dual bronchodilator; Umeclidinium/Vilanterol)
- Triple inhaler (dual bronchodilator plus a corticosteroid; Umeclidinium/ Vilanterol & Fluticasone Furoate)
- Placebo inhaler (a 'dummy' treatment)

The dual inhaler contains 2 active medicines: Umeclidinium and Vilanterol; these both belong to a group of medicines called bronchodilators. Bronchodilators widen the muscles around the airways in the lungs which makes it easier for air to get in and out of the lungs. When used regularly, bronchodilators can help to control breathing difficulties.

The triple inhaler contains 3 active medicines: Umeclidinium, Vilanterol and Fluticasone Furoate. Umeclidinium and Vilanterol are the same bronchodilator medicines that are in the dual inhaler. The Fluticasone Furoate is a steroid medicine. Bronchodilators widen the muscles in the lungs and steroids reduce swelling and irritation in the small air passages which makes it easier for air to get in and out of the lungs. When used regularly bronchodilators and steroids can help to control breathing difficulties.

The placebo inhaler does not contain any active medicines; it contains a dry powder that should not have any effect on you.

The dual and triple inhalers have already been tested in large research studies for safety, side effects, and effectiveness in treating COPD and have been licenced for use in the treatment of COPD. The DIBS study is a phase 3 clinical trial. A phase 3 clinical trial is a study which includes a large number of patients to look at whether a treatment works well. We hope to see in DIBS whether bronchodilators and/or bronchodilators and steroids, which have been used already to treat COPD, are effective treatments for patients with bronchiectasis.

Morton M, *et al. BMJ Open* 2023; 13:e071906. doi: 10.1136/bmjopen-2023-071906

The inhalers all look the same. You will have four times the chance of getting an active inhaler as being in the placebo group (i.e. if there are 5 participants, 2 will have the dual inhaler, 2 will have the triple inhaler, and 1 will have the placebo inhaler).

Which inhaler you are given will be completely random. You and the healthcare professionals involved in the study will not know which type of inhaler you are allocated to.

You will be prescribed study inhalers for 12 months (365 days in total). Each inhaler has 30 doses and you need to take one puff of your inhaler every day for 12 months (365 days in total). You should try to take your inhaler at the same time every day. You will be given your first inhaler at your screening/baseline hospital visit and then receive replacement inhalers by post. The inhalers will be sent by recorded delivery and you may need to sign to confirm you have received them. On study visit days, do **not** use your inhaler before you arrive in hospital. You will be asked to bring your current inhaler to hospital and to take your daily dose whilst attending hospital. This is to check whether you are using your inhaler correctly.

PLEASE KEEP ALL OF YOUR USED INHALERS

It is vitally important that you keep hold of all your study inhalers, even the empty ones. You will be asked to return all inhalers at your next study visit so that the number of doses you have used can be checked and recorded.

WHAT ARE THE POSSIBLE BENEFITS OF TAKING PART?

Patients with other lung conditions where these inhalers are already proven to work reported better lung function, better quality of life and less exacerbations (or flare ups). We cannot promise this study will help you directly but the information we get from this study may help to improve treatment for people with bronchiectasis in the future. If you want to find out more about taking part in research studies you can visit the NHS Choices website https://www.nhs.uk/conditions/clinical-trials/

Morton M, *et al. BMJ Open* 2023; 13:e071906. doi: 10.1136/bmjopen-2023-071906

EXPENSES

Your travel expenses (up to £25 per visit) for each hospital visit will be paid for by the study. Alternatively, transport may be arranged for you if your local hospital is able to offer this. In addition, should you prefer to contact the study team for your monthly telephone call rather than the study team telephoning you, the costs of these telephone calls will be paid for by the study.

Your study team will manage any payments to reimburse costs to you and you may be asked to provide receipts for your travel.

WILL MY GP KNOW THAT I'M TAKING PART IN THE STUDY?

Yes, we will send a letter to your GP to inform them that you are taking part in this study, and a copy will be filed in your hospital notes. This is so that your medical records at your GP practice and in hospital contain documentation that you are taking part in a clinical study. Any test results from taking part in this study will also be added to your medical records. Your GP will be asked to inform the study team of any adverse reactions to the study medication or emergency hospital admissions.

Your GP will not know if you have received active treatment or placebo.

WHAT ARE THE POSSIBLE DISADVANTAGES AND RISKS OF TAKING PART?

We want you to be safe in this study at all times, but all medical treatments carry some risk. The inhalers used in this study are widely used by NHS patients with respiratory conditions, and there are some known side effects.

If you react badly to the inhaler your study doctor will be able to change your medication and treat you to try to ease your symptoms. If they need to find out which treatment you are taking (one of the two active treatments or placebo), this information is available in cases of emergency.

If you feel unwell during your time in the study then please contact your research team or study doctor.

POSSIBLE SIDE EFFECTS

As with most medicines the study inhalers do have some side effects which are listed in the table below. As these drugs are widely used in COPD our experience is that very few of these side effects are common causes for patients to stop their inhalers. If you are assigned to the triple inhaler group there may be a higher risk of you getting pneumonia. This is because the steroid inhalers have been shown to make the risk of getting pneumonia higher in patients with COPD. Pneumonia is when there is swelling of the tissue in one or both lungs, usually caused by an infection. The steroid inhaler reduces COPD exacerbations a lot more than the risk of pneumonia it brings to these patients. If you experience any of the side effects listed in the table below, it is important that you let your study team know straight away. The study team can talk to you more about what these mean if you are unsure about any of them. These side effects often are temporary.

**Frequency**	**Dual inhaler (dual bronchodilator)**	**Triple inhaler (dual bronchodilator and corticosteroid)**
**Common or very common**	▪ Increased risk of infection<br>▪ Cough<br>▪ Throat pain<br>▪ Constipation<br>▪ Dry mouth<br>▪ Headache	▪ Increased risk of infection<br>▪ Headache<br>▪ Cough<br>▪ Throat pain<br>▪ Constipation<br>▪ Joint pain<br>▪ Back pain<br>▪ Thrush in the mouth and throat
**Uncommon**	▪ Rash<br>▪ Tremor<br>▪ Altered taste<br>▪ Abnormal heart rhythms<br>▪ Palpitations<br>▪ Hoarse voice	▪ Abnormal heart rhythms<br>▪ Dry mouth<br>▪ Bone fracture<br>▪ Altered taste<br>▪ Hoarse voice<br>▪ Chest infection
**Rare or very rare**	▪ Swelling<br>▪ Anaphylaxis<br>▪ Blurred vision<br>▪ Glaucoma	▪ Swelling<br>▪ Anaphylaxis<br>▪ Skin rash

	▪ Pressure inside the eye<br>▪ Tightening of air passages (sometimes severe)<br>▪ Urinary problems	
**Frequency unknown***	▪ Dizziness	▪ Blurred vision

* the frequency of these side effects are unknown as there is not enough data available at the moment to estimate the frequency.

You could be randomised to receive the placebo 'dummy' treatment. If you get the placebo treatment it could be the case that you experience more exacerbations than on your current treatment/inhaler. You and the study team will not know which treatment you are receiving.

PREGNANCY AND CONTRACEPTION

As we do not know if the study medicines will harm an unborn baby those planning to become pregnant (or their partner becoming pregnant) will be excluded from the study.

To prevent pregnancy during the study, all participants of childbearing potential must use contraception from the day that consent is given to take part in the study up until 7 days after the last dose of study medication is taken.

For male participants contraceptive methods include:

- condom
- practice true abstinence in line with preferred and usual lifestyle

For female participants contraceptive methods include:

- combined hormonal contraception (oral, intravaginal, transdermal)
- progestogen only hormonal contraception (oral, injectable, implantable)
- intrauterine device (IUD)
- intrauterine hormone-releasing system (IUS)
- vasectomised partner
- bilateral tubal occlusion

Morton M, et al. BMJ Open 2023; 13:e071906. doi: 10.1136/bmjopen-2023-071906

- practice true abstinence in line with preferred and usual lifestyle

If you or your partner become pregnant during the course of the study, you must tell your study doctor **immediately** so appropriate action can be discussed.

If you do become pregnant during the study, you must stop taking your study inhaler. We will ask you to return your study inhaler at your next follow-up visit.

We will monitor your pregnancy carefully and will ask if we can collect some information on the health of your baby when it is born. A children's doctor will also be asked to check your baby carefully when it is born.

If your partner becomes pregnant during the study, we will ask them to sign a consent form. This will allow the study team to collect safety information about their pregnancy and their baby.

BREASTFEEDING

If you are breastfeeding you will not be allowed to take part in the study. This is because we don't know if the study medicine is passed into breastmilk or if it is safe in babies.

WHAT WILL HAPPEN TO ME WHEN THE STUDY ENDS?

You will stop taking the study inhalers and you will continue to receive standard care like any other patient with your condition, under the care of your respiratory doctor. You and your respiratory doctor can discuss which treatment would be best for you at that time. You and your doctors will not find out which inhaler you were taking during the study.

WHAT IF SOMETHING GOES WRONG?

If you have a concern about any aspect of this study, you can speak to a member of the study team who will do their best to answer your questions. Further contact details are included at the end of this information sheet. If you are still unhappy and wish to raise your concerns with someone who is not directly involved in your care, you can contact <site to localise with local details such as PALS (or equivalent) phone number and email address>

In the unlikely event that you are harmed during the study and this is due to someone's negligence (they were careless) you may have grounds for legal

Morton M, *et al. BMJ Open* 2023; 13:e071906. doi: 10.1136/bmjopen-2023-071906

action and compensation, but you may need to meet your own legal costs. NHS Indemnity does not offer no-fault compensation (for harm that is not anyone's fault).

The Newcastle Clinical Trials Unit, part of Newcastle University, are managing the study on behalf of the study NHS Sponsor. Newcastle University also have insurance arrangements in place to cover Newcastle University staff involved in designing and managing the DIBS study.

WHO IS ORGANISING AND FUNDING THE STUDY?

The doctor in charge of the study (the Chief Investigator) is Professor Anthony De Soyza, a Respiratory Consultant. He is based in Newcastle upon Tyne.

Study Sponsor: Newcastle upon Tyne Hospitals NHS Foundation Trust. The study Sponsor has responsibility for the study. The study is managed by the Newcastle Clinical Trials Unit, Newcastle University, on behalf of the Sponsor.

Study Funder: National Institute for Health Research Health Technology Assessment programme. This body is funded by the UK government to carry out research for the benefit of the NHS and its patients.

GlaxoSmithKline plc, a pharmaceutical company, have supported the study by providing their inhalers to the study team free of charge.

WHO HAS REVIEWED THIS STUDY?

This study was reviewed and approved by the Research Ethics Committee (North East- Newcastle & North Tyneside 2), the Health Research Authority (HRA) and the Medicines and Healthcare products Regulatory Agency (MHRA). The MHRA are responsible for approving all studies involving medicines. The Newcastle Upon Tyne Hospitals NHS Foundation Trust has reviewed all the study documentation and assessed the risks of this study as part of their responsibility, as study Sponsor. This is to ensure that we are not doing anything harmful to you during the study and that your data is collected safely and stored securely. Bronchiectasis patients helped to design this study. They have also looked at the patient information sheet, the consent form and the exacerbation diary.

WHO IS PROVIDING THE STUDY INHALERS?

Morton M, *et al. BMJ Open* 2023; 13:e071906. doi: 10.1136/bmjopen-2023-071906

The inhalers for all three treatment groups in this study have been provided free of charge by GlaxoSmithKline plc, a pharmaceutical company. They recognise patients with bronchiectasis have no licenced therapies.

WHAT IF RELEVANT NEW INFORMATION BECOMES AVAILABLE?

If, during the course of the study, new information becomes available that is relevant to you, we will tell you about it. We will discuss whether you should or would like to stop the study treatment or withdraw from the study.

WHAT WILL HAPPEN TO THE RESULTS OF THE RESEARCH STUDY?

- The results will be written in medical journals and presented in meetings to other doctors, nurses, researchers and patients.
- A report will be written for the study funder and put on their website.
- All study data that is published will be anonymous. Your identity will always be protected.
- The results will be available at the end of the study through publications, in the wider press and directly to patient groups. You will be able to request a copy of the summary of the results from your study hospital.
- Fully anonymised data may be made available to other researchers to help inform other research studies.

WILL MY TAKING PART IN THIS STUDY BE KEPT CONFIDENTIAL?

Yes. All of the information collected in the study will be entered on computers that are kept secure and password protected.

- You will be given a unique study identification number (called a Participant ID Number) instead of your name being written on study documents. The study team at your hospital will be able to link this number back to you using your date of birth, name, and NHS number (or other nations equivalent). A restricted number of the study team at the Newcastle Clinical Trials Unit may have access to your date of birth, Participant ID number and NHS numbers together. This is for the purpose of requesting the 24 month follow up data, depending on when you join the study.

Morton M, et al. BMJ Open 2023; 13:e071906. doi: 10.1136/bmjopen-2023-071906

- The study team at your hospital will have access to your information during the study to organise planned visits as well as for ongoing safety. They will be in contact with you about receipt of your replacement inhalers and your health
- Your hospital pharmacy/study team will have access to your contact details for posting your replacement study medication
- Your contact details will never be shared with anyone outside of the study with the exception of Royal Mail or an alternative postal/courier service used by your local hospital. You will be asked to consent to the postal service your local hospital uses to have access to your contact details so that they can deliver your replacement inhalers to your home address. The postal services will not know you are in the study just that they need to deliver a package to you
- You will not be named in any results, reports or on websites
- Very occasionally, information might be given during the study that we would have a legal obligation to pass on to others (for instance information which suggested you or others were at risk of harm). In this case, confidentiality would be broken so that we could pass this information to the relevant people. You would be informed of this
- At the end of the study, all study information will be kept in a secure storage area (this is called archiving) for at least 5 years. This makes sure any queries about the running of the study have been answered. All information will be held securely to make sure we protect your confidentiality, after which it will be safely destroyed
- If there are any serious adverse events, we would send details of them to the government medicines agency (MHRA); only your participant ID number would be sent to them. This information may also be sent to researchers outside of the United Kingdom (UK) in the European Economic Area (EEA) who are involved in overseeing the study.
- Some parts of your medical records and the data collected for the study may be looked at by authorised persons from the MHRA, Sponsor (Newcastle Hospitals NHS Foundation Trust) and or the Newcastle Clinical Trials Unit to check that the study is being conducted to the correct standards. All will have a duty of confidentiality to you as a research participant.

- GlaxoSmithKline plc have asked for de-identified data (age, ethnicity, sex at birth (male/female), site number and disease status and new participant identifier) to be shared with them. This is to allow them to complete their own analyses. This data may be used by anyone in the international company and may leave the European Economic Area (EEA).
- In order to learn about the long-term health of participants in this study your NHS number (or other nations equivalent) may be sent to the Newcastle Clinical Trials Unit, part of Newcastle University. Your information would be transferred and stored securely with access limited to the people that need to know this information.

HOW WILL WE USE INFORMATION ABOUT YOU?

We will need to use information from you, from your medical records and from your GP for this research project.

This information will include your initials, date of birth, NHS number (or other nations equivalent), name and contact details. People will use this information to do the research or to check your records to make sure that the research is being done properly.

People who do not need to know who you are will not be able to see your name or contact details. Your data will have a code number, your unique Participant ID, instead.

We will keep all information about you safe and secure.

Once we have finished the study, we will keep some of the data so we can check the results. We will write our reports in a way that no-one can work out that you took part in the study.

WHAT ARE YOUR CHOICES ABOUT HOW YOUR INFORMATION IS USED?

- You can stop being part of the study at any time, without giving a reason, but we will keep information about you that we already have.

- If you choose to stop taking part in the study, we would like to continue collecting information about your health from central NHS (or other nations equivalent) records, your hospital and your GP. If you do not want this to happen, tell us and we will stop.
- We need to manage your records in specific ways for the research to be reliable. This means that we won't be able to let you see or change the data we hold about you.

WHERE CAN YOU FIND OUT MORE ABOUT HOW YOUR INFORMATION IS USED?

You can find out more about how we use your information

- at www.hra.nhs.uk/information-about-patients/
- our leaflet available from https://www.newcastle-hospitals.nhs.uk/help/privacy/privacy-notice-for-patients/
- by asking one of the research team
- by sending an email to the Sponsor Data Protection Officer at nuth.dpo@nhs.net
- by ringing the Newcastle upon Tyne Hospital Data Protection Officer on 0191 223 1474

FURTHER INFORMATION AND CONTACT DETAILS

If you have any further questions or would like any further information about the study or the rights of participants, please feel free to contact the people below.

They are also who you or your doctor should contact in the event of an emergency, if your study participation is in any way involved.

[LOCAL CONTACT DETAILS]

Thank you for reading this information sheet.

Morton M, *et al. BMJ Open* 2023; 13:e071906. doi: 10.1136/bmjopen-2023-071906

Newcastle University

Print on Trust headed paper

Dual Bronchodilators in Bronchiectasis Study (DIBS)

DIBS

INFORMED CONSENT FORM

V2.0 29th March 2021

Participant ID: | | | | | | |

Principal Investigator: ___________________________________

		Please INITIAL the boxes if
1.	I confirm that I have read the Participant Information Sheet dated __/___/____ version ___ for the above study and that I have had the opportunity to consider the information, ask questions and have had these answered satisfactorily.	
2.	I understand that my participation is voluntary and that I am free to withdraw at any time without giving any reason, without my medical care or legal rights being affected.	
3.	I consent to receiving the study medication outlined in the Participant Information Sheet.	
4.	I understand that my personal data (name, address, telephone number) will be used for the purposes of posting study inhalers to my home address and for contacting me. I give permission for this information to be stored by responsible people at my local NHS Trust and for them to be transferred to Royal Mail, or any alternative postal or courier service used by my local hospital, so that my replacement inhalers can be posted to my home address.	
5.	I understand that information about me that is relevant to this study will leave the Trust and be sent securely to Newcastle University. This includes my date of birth, gender and ethnicity which will be stored on the study database. My NHS number will also be collected and stored separately in order to collect data about my long-term health, and could be accessed for up to three years after my enrollment into this study. I understand that my data will be stored securely and managed confidentially as part of this study.	
6.	I understand that any personal information collected about me for the study will be kept confidential and not be made public. I understand that data from the study will be	

Morton M, *et al. BMJ Open* 2023; 13:e071906. doi: 10.1136/bmjopen-2023-071906

	published in medical journals, at research meetings and shared with other researchers, including researchers potentially outside the United Kingdom (UK) in the European Economic Area (EEA). I understand that data from the study will be de-identified and that I will not be directly identified in the published results.	
7.	I understand that de-identified data from the study will be shared with GlaxoSmithKline plc (GSK), including the following personal information:<br>• Age<br>• Sex at birth (female/male)<br>• Information about my bronchiectasis disease<br>• Ethnicity<br>• Study identification number<br>I understand that I will not be directly identified in any data shared with GSK.	
8.	I consent to study data being transferred to GSK plc. I understand that GSK is an international company and that data may be transferred outside of the United Kingdom (UK).	
9.	I understand that the information held and maintained by:<br>• Bronchiectasis Databases (including BronchUK [The United Kingdom Bronchiectasis Registry] and EMBARC [European Multicentre Bronchiectasis Audit and Research Collaboration], if you have previously consented to take part in these projects)<br>• NHS Digital (including Hospital Episodes Statistics [HES]-not applicable for Scottish sites)<br>• Central UK NHS bodies (including the Office for National Statistics [ONS]-not applicable for Scottish sites)<br>may be used to provide information about my health status to the research team. I consent to the research team requesting information about my health from routine sources, including Bronchiectasis Databases, NHS Digital and ONS (if applicable) as needed, including up to one year after my participation in the study finishes, and to reviewing my medical records to compare this information.	
10.	I understand that the information collected about me will be used to support other research in the future, and may be shared anonymously with other researcher projects and researchers.	

Morton M, *et al. BMJ Open* 2023; 13:e071906. doi: 10.1136/bmjopen-2023-071906

11.	I understand that parts of my medical records and data collected during the study may be looked at by responsible people. I give my permission for these people to have access to my medical records.<br>This includes people from Newcastle University, study Sponsor, regulatory authorities and local NHS Trust where it is relevant to my taking part in research.	
12.	I understand that the information provided in this study is being managed by the Newcastle Clinical Trials Unit, which is part of Newcastle University.	
13.	I agree to my General Practitioner being involved in the study, including any necessary exchange of information about me between my GP and the research team	
14.	I agree to the information provided and this signed consent form being stored for 5 years after the end of the study.	
15.	I agree to take part in the above study.	

For participants of childbearing potential:		
16.	I understand that I will have to use an effective form of contraception as outlined in the Participant Information Sheet, if sexually active.	
17.	Females only: I understand that if I am of childbearing potential, I will need to have a urine pregnancy test to ensure that I am not pregnant. I understand that this is for safety reasons.	

Name of Participant	Date	Signature
Name of Person taking consent	Date	Signature

When completed: File 1 copy in the investigator site file (original); Provide 1 copy for the study participant; File 1 copy in the participant's medical notes.

IRAS ID: 275595 | DIBS Informed Consent Form | V2.0 | 29 March 2021
