# Supplementary material for: Dual bronchodilators in Bronchiectasis study (DIBS): protocol for a pragmatic, multicentre, placebo-controlled, three-arm, double-blinded, randomised controlled trial studying bronchodilators in preventing exacerbations of bronchiectasis
Source: BMJ Open. 2023 Aug 10;13(8):e071906. doi: 10.1136/bmjopen-2023-071906 (PMC10423789; doi:10.1136/bmjopen-2023-071906)
Supplement: Author's manuscript [file bmjopen-2023-071906.draft_revisions.pdf]

BMJ Open

BMJ Open is committed to open peer review. As part of this commitment we make the peer review history of every article we publish publicly available.

When an article is published we post the peer reviewers' comments and the authors' responses online. We also post the versions of the paper that were used during peer review. These are the versions that the peer review comments apply to.

The versions of the paper that follow are the versions that were submitted during the peer review process. They are not the versions of record or the final published versions. They should not be cited or distributed as the published version of this manuscript.

BMJ Open is an open access journal and the full, final, typeset and author-corrected version of record of the manuscript is available on our site with no access controls, subscription charges or pay-per-view fees (http://bmjopen.bmj.com).

If you have any questions on BMJ Open's open peer review process please email info.bmjopen@bmj.com

BMJ Open

Dual Bronchodilators in Bronchiectasis Study (DIBS): protocol for a pragmatic, multicentre, placebo controlled, 3 arm, double-blinded, randomised controlled trial studying bronchodilators in preventing exacerbations of bronchiectasis

Journal:	*BMJ Open*
Manuscript ID	bmjopen-2023-071906
Article Type:	Protocol
Date Submitted by the Author:	27-Jan-2023

Complete List of Authors:	Morton, Miranda; Newcastle University, Newcastle Clinical Trials Unit<br>Wilson, Nina; Newcastle University Faculty of Medical Sciences, Population Health Sciences Institute<br>Homer, Tara; Newcastle University, Health Economics Group<br>Simms, Laura; Newcastle University,<br>Steel, Alison; Newcastle University, Newcastle Clinical Trials Unit<br>Maier, Rebecca; Newcastle University, Newcastle Clinical Trials Unit<br>Wason, James; Newcastle University, Population Health Sciences Institute<br>Ternent, Laura; Newcastle University, Health Economics Group<br>Abouhajar, Alaa; Newcastle University, Newcastle Clinical Trials Unit<br>Allen, Maria; The Newcastle upon Tyne Hospitals NHS Foundation Trust<br>Joyce, Richard; Newcastle University, Newcastle Clinical Trials Unit<br>Hildreth, Victoria; Newcastle University, Newcastle Clinical Trials Unit<br>Lakey, Rachel; Newcastle University, Newcastle Clinical Trials Unit<br>Cherlin, Svetlana; Newcastle University<br>Walker, Adam; The Newcastle upon Tyne Hospitals NHS Foundation Trust<br>Devereux, Graham ; Liverpool School of Tropical Medicine, Department of Clinical Sciences<br>Chalmers, J; University of Dundee,<br>Hill, A; The University of Edinburgh<br>Haworth, Charles; Royal Papworth Hospital NHS Foundation Trust<br>Hurst, John; UCL Medical School,, Academic Unit of Respiratory Medicine<br>De Soyza, Anthony; Newcastle University

Keywords:	RESPIRATORY MEDICINE (see Thoracic Medicine), Adult thoracic medicine < THORACIC MEDICINE, Clinical trials < THERAPEUTICS

SCHOLARONE™
Manuscripts

Dual Bronchodilators in Bronchiectasis Study (DIBS): protocol for a pragmatic, multicentre, placebo controlled, 3 arm, double-blinded, randomised controlled trial studying bronchodilators in preventing exacerbations of bronchiectasis

Authors: Miranda Morton[1], Nina Wilson[2], Tara Homer[3], Laura Simms[1], Alison Steel[1], Rebecca Maier[1], James Wason[2], Laura Ternent[3], Alaa Abouhajar[1], Maria Allen[4], Richard Joyce[1], Victoria Hildreth[1], Rachel Lakey[1], Svetlana Cherlin[2], Adam Walker[5], Graham Devereux[6], James Chalmers[7], Adam Hill[8], Charles Haworth[9], John Hurst[10], Anthony De Soyza[11]

[1] Newcastle Clinical Trials Unit, Newcastle University, Newcastle upon Tyne, UK. [2] Biostatistics Research Group, Newcastle University, Newcastle upon Tyne, UK. [3] Health Economics Group, Newcastle University, Newcastle upon Tyne, UK. [4] R&D Pharmacy, The Newcastle upon Tyne Hospitals NHS Foundation Trust, Newcastle upon Tyne, UK. [5] Newcastle Specials (Pharmacy Production Unit), The Newcastle upon Tyne Hospitals NHS Foundation Trust, Newcastle upon Tyne, UK. [6] Liverpool School of Tropical Medicine, Liverpool, UK. [7] School of Medicine, University of Dundee, Dundee, UK. [8] Department of Respiratory Medicine, Royal Infirmary of Edinburgh, Edinburgh, UK. [9] Cambridge Centre for Lung Infection, Royal Papworth Hospital NHS Foundation Trust, Cambridge, UK. [10] Centre for Inflammation and Tissue Repair, UCL, London, UK. [11] Faculty of Medical Sciences, Newcastle University, Newcastle upon Tyne, UK.

Corresponding Author: Anthony De Soyza, Professor of Pulmonary Medicine. Postal address: Population Health Sciences Institute, Faculty of Medical Sciences, Newcastle University, Room L2060, Framlington Place, Newcastle upon Tyne, NE2 4HH. Email address: Anthony.de-soyza@newcastle.ac.uk

Key words: Bronchiectasis, Bronchodilators, Inhaled steroid

Word count (excluding title page, abstract, references, figures and tables): 5095

For peer review only

For peer review only - http://bmjopen.bmj.com/site/about/guidelines.xhtml

ABSTRACT

Introduction

Bronchiectasis is a long-term lung condition, with dilated bronchi, chronic inflammation, chronic infection and acute exacerbations. Recurrent exacerbations are associated with poorer clinical outcomes such as increased severity of lung disease, further exacerbations, hospitalisations, reduced quality of life and increased risk of death. Despite an increasing prevalence of bronchiectasis, there is a critical lack of high-quality studies into the disease and no treatments specifically approved for its treatment. This trial aims to establish whether inhaled dual bronchodilators (long acting beta agonist [LABA] and long acting muscarinic antagonist [LAMA]) taken as either a stand-alone therapy or in combination with inhaled corticosteroid (ICS) reduce the number of exacerbations of bronchiectasis requiring treatment with antibiotics during a 12 month treatment period.

Methods

This is a multicentre, pragmatic, double blind, randomised controlled trial, incorporating an internal pilot and embedded economic evaluation. 600 adult patients (≥ 18 years) with CT confirmed bronchiectasis will be recruited and randomised to either inhaled dual therapy (LABA + LAMA), triple therapy (LABA + LAMA + ICS) or matched placebo, in a 2:2:1 ratio (respectively). The primary outcome is the number of protocol defined exacerbations requiring treatment with antibiotics during the 12 month treatment period.

Ethics and Dissemination

Favourable ethical opinion was received from the North East – Newcastle & North Tyneside 2 Research Ethics Committee (reference: 21/NE/0020). Results will be disseminated in peer reviewed publications, at national and international conferences, in the NIHR HTA journal and to participants and the public (using lay language).

Registration

ISRCTN15988757

STRENGTHS AND LIMITATIONS OF THIS TRIAL

- Significant unmet clinical need for patients with bronchiectasis and a critical lack in research into bronchiectasis
- Trial aims to advance the evidence basis for inhaled treatments in bronchiectasis
- Challenge of a large multicentre trial in the respiratory disease area in the peri COVID-19 era
- Changing biology of bronchiectasis following shielding behaviours during the COVID-19 pandemic

For peer review only - http://bmjopen.bmj.com/site/about/guidelines.xhtml

For peer review only

INTRODUCTION

Bronchiectasis is a chronic lung condition, characterised by dilated bronchi, leading to symptoms of breathlessness and chronic productive cough, with intermittent infective exacerbations. Bronchiectasis has various potential aetiologies including immune-deficiency syndromes, allergic bronchopulmonary aspergillosis, chronic obstructive pulmonary disease, ciliary dysfunction and post-infectious, yet studies have found that between a quarter and half of cases are idiopathic [1, 2]. Patients often have recurrent, costly hospital admissions, a poorer quality of life [3, 4] and clinically significant fatigue [5, 6].

Current estimates suggest a prevalence of 100,000 adult patients with the condition in the UK. Importantly, studies demonstrate that up to 50% of patients with chronic obstructive pulmonary disease (COPD) have co-existent bronchiectasis [7]. With approximately 1,000,000 patients with COPD in the UK [8], there is potential for a significant increase of case-finding of COPD-associated bronchiectasis over the coming years.

Bronchiectasis mortality rates are approximately 50% higher than that of uncomplicated COPD (calculated at 3% per annum) and have been reported to be increasing [9]. Prognosis varies, with a prior study of 91 patients [10] finding that the primary cause of death was usually respiratory, with survival rates of 91% at 4 years and 68.3% at 12.3 years. The same study found factors such as chronic infection with *Pseudomonas aeruginosa* increase mortality.

Multicentre data suggests the Bronchiectasis severity index (BSI) a multi component clinical scoring system is useful in predicting both mortality and morbidity (hospitalisation). This score was validated in 1300 patients across Europe [11].

Infective exacerbations lead to significant morbidity. Within the UK national audit data patient group average exacerbation rate is approximately 3 per year (nearly twice the rate of COPD) with an attendant risk of hospitalisation. This is consistent with published American data on the increasing burden of bronchiectasis [12] and in the UK [13]. Previously published UK data also emphasises the burden of bronchiectasis, uncertainties in aetiology and lack of evidence for the treatments that are often used [14]. Hence, improved interventions in bronchiectasis are urgently required.

There is no cure for bronchiectasis. Current modalities of treatment include oral, inhaled or intravenous antibiotics given regularly with additional courses administered for exacerbations. Mucolytics and regular physiotherapy are used to aid sputum clearance and there are additional guidelines for investigation, diagnosis and management of bronchiectasis produced by the British Thoracic Society (BTS) [15].

For peer review only - http://bmjopen.bmj.com/site/about/guidelines.xhtml

National audits in the UK suggest up to 80% of patients with bronchiectasis are on inhaled therapy despite limited evidence [15]. Prior studies of inhaled cortico-steroids (ICS) in bronchiectasis have shown no clear benefit [15] and are therefore not recommended in prevailing bronchiectasis guidelines. ICS, however, have been shown to have benefits in asthma [16] and in COPD [17]. More recent data emerging from COPD suggests that ICS reduce COPD exacerbation rates but increases pneumonia rates, a side effect that may be very pertinent in bronchiectasis. International guidelines for COPD now suggest a selective application of ICS and that not all patients with COPD benefit from ICS therapy [17]

The role of triple therapy (ICS/LABA/LAMA) and dual bronchodilation (LABA/LAMA) therapy is unclear in bronchiectasis with no robust randomised placebo-controlled data available. Given the likely higher bacterial and inflammatory load in bronchiectasis airways these therapies need be specifically studied within this population

METHODS AND ANALYSIS

Trial methods and analysis are reported as per SPIRIT reporting guidelines [18].

Trial Design

DIBS is a pragmatic, multi-centre, placebo-controlled, three-arm, double blinded, randomised controlled trial, incorporating an internal pilot. The trial aim is to recruit a total of 600 adult patients with bronchiectasis from up to 25 secondary care NHS sites. The trial is Sponsored by The Newcastle upon Tyne Hospitals NHS Foundation Trust (tnu-tr.sponsormanagement@nhs.net).

The internal pilot involves approximately 15 secondary care NHS sites recruiting for a 12-month recruitment period (following activation of the first site to recruitment). Site activation of the NHS sites will be as soon as possible with a staggered approach. The recruitment aim is to recruit 98-125 participants equating to an average recruitment between 1.25 and 1.6 participants recruited per site per month. At the end of the internal pilot a review of will be made by the Trial Management Group (TMG), the Trial Steering Committee (TSC), the Independent Data Monitoring and Ethics Committee (IDMEC) and a consultation with the funder against the following progression criteria to proceed from the internal pilot to the main trial recruitment:

- Average recruitment ≥ 1.6 participants/month/site activated – continue to main trial and open additional sites (up to 25 sites total)
- Average recruitment ≥ 1.25 participants/month/site activated – continue to main trial and open additional sites (up to 25 sites total) plus provide an improvement plan after identifying barriers to recruitment through discussion with sites, TMG, TSC and IDMEC as required

For peer review only - http://bmjopen.bmj.com/site/about/guidelines.xhtml

- Average recruitment < 1.25 participants/month/site activated – seek further guidance from funder

The TSC and IDMEC are composed of independent experts in the field (clinicians, statisticians) and patient a public representatives. Each committees' roles are defined in their charters.

The main trial phase will follow on from the pilot without a break in recruitment. The main phase of the trial will involve an additional 10 secondary care NHS sites bringing the total number of sites recruiting to the trial to 25 sites. The recruitment target for each site remains 1 – 2 patients per month throughout the main phase of recruitment which will last for 14 months. The last patient last visit will be 12 months after the last participant has been recruited.

Patient eligibility – Inclusion and Exclusion Criteria

Patients are eligible for the trial is all of the following inclusion criteria apply:

1) Adult patients with CT (Computed Tomography) scan confirmed bronchiectasis and bronchiectasis is the predominant primary respiratory disease in the view of the investigator (CT images / CT reports must be available to complete radiological scoring for Bronchiectasis Severity Index)
2) History of 2 or more exacerbations in any 12-month period in the preceding 2 years requiring antibiotics and/or steroids
3) Evidence of airflow limitation with an FEV1/FVC ratio less than 0.7 and/or daily mucus expectoration
4) Have either (i) less than 20 pack year history of smoking OR (ii) greater than 20 pack year history of smoking with FEV1 >79% predicted (to exclude COPD)
5) For patients taking ICS, LAMA or LABA treatment prior to recruitment, willing to have these treatments changed or stopped
6) Stable bronchiectasis with no exacerbations for 4 weeks prior to baseline
7) Stable dose of oral steroid for 4 weeks prior to baseline (only applicable for patients taking oral steroid as part of standard care)

Patients are excluded from the trial if any of the following exclusion criteria apply:

1) Cystic fibrosis related bronchiectasis
2) Where bronchiectasis is not the main disease or there are contraindications to ICS withdrawal
3) Predominant COPD or asthma *

For peer review only - http://bmjopen.bmj.com/site/about/guidelines.xhtml

*Patients who have a historical diagnosis of asthma and/or COPD but where the investigator has sufficient evidence to refute these diagnoses can still be included

4) Indication to remain on ICS (e.g. asthma, COPD, allergic bronchopulmonary aspergillosis, inflammatory bowel disease) or known intolerance to any of the trial drugs or their ingredients
5) Patients with galactose intolerance, total lactase deficiency or glucose-galactose malabsorption
6) Inability to perform spirometry or quality of life questionnaires
7) Patients who are:
   a) pregnant
   b) breast feeding
   c) of childbearing potential with a positive urine pregnancy test prior to starting trial IMP
   d) male or female of childbearing potential unwilling to use contraception throughout the trial (postmenopausal women must be amenorrhoeic for at least 12 months to be considered of non-childbearing potential).
8) Anyone with cognitive impairment who may not be able to consent
9) Those who do not speak English or cannot comply with trial procedures
10) Any potential participant who the investigator believes will not be able to complete the trial visits and procedures
11) A history of allergy or hypersensitivity to any corticosteroid, anticholinergic/muscarinic receptor antagonist, β2-agonist, lactose/milk protein or magnesium stearate or a medical condition such as narrow-angle glaucoma, prostatic hypertrophy or bladder neck obstruction that, in the opinion of the investigator contraindicates trial participation
12) Use of acute antibiotics or systemic steroids within 4 weeks of baseline (except for antibiotics and/or stable doses of prednisone ≤ 5mg used to treat non-respiratory conditions)
13) Malignancy diagnosed within 5 years of the first trial medication administration where the investigator feels the trial may be affected by recurrence or progression of the malignancy (e.g. patients with stable breast cancer, current prostate cancer or “expected curative” cancer surgery) may not be excluded at the investigators discretion
14) Administration of an investigational agent within 30 days of first dose of trial medication

Objectives and Outcomes

The primary objective for the trial is to compare the effects of inhaled dual bronchodilators either as a stand-alone dual therapy (LABA + LAMA) or in combination with inhaled corticosteroid as a triple therapy (LABA + LAMA + ICS) to placebo on the number of protocol defined bronchiectasis

For peer review only - http://bmjopen.bmj.com/site/about/guidelines.xhtml

exacerbations experienced per participant over the 12-month trial treatment period. The protocol definition of a bronchiectasis exacerbation is a continued worsening of one or more of symptoms including increased cough, sputum discolouration, excess sputum production, breathlessness and/or fatigue, that require treatment with antibiotic(s).

Additionally, there is a primary economic objective to compare the cost-effectiveness, measured in terms of incremental cost per quality-adjusted life-year (QALY) gained over the 12-month trial treatment period.

Secondary objectives include comparisons between the effects of dual therapy (LABA + LAMA), triple therapy (LABA + LAMA + ICS) and placebo on the number of bronchiectasis related hospital admissions, the number of emergency hospital admissions, the time to the first exacerbation of bronchiectasis following randomisation, the number of adverse events and cessation of treatment, quality of life, breathlessness and lung function, respiratory and cardiac mortality, economic costs and the incremental cost per QALY gained over the patient's life course. Objectives and Outcome measures are described fully in

Table 1.

Table 1. Objectives and outcome measures

	Objectives	Outcome Measures
**Primary**	Compare effects of inhaled dual bronchodilators either as a stand-alone therapy (LABA/LAMA) or in combination with ICS (ICS/LABA/LAMA; triple therapy) therapy to placebo on the number of protocol defined bronchiectasis exacerbations (per participant)	• Number of protocol defined bronchiectasis exacerbations requiring treatment with antibiotics during 12-month treatment period
**Primary Economic**	Compare cost-effectiveness measured in terms of incremental cost-per-QALY gained over the 12 month treatment period.	• Incremental cost-per QALY at 12 months<br>• Participant costs (time and travel) at 12 months
**Secondary**	To compare the effects of dual bronchodilators (LABA/LAMA) and triple (ICS/LABA/LAMA) therapy and placebo on:	
	• Hospital admissions with a primary diagnosis of exacerbation of bronchiectasis	• Number of hospital admissions for bronchiectasis exacerbations during 12-month treatment period<br>• Hospitalisation due to bronchiectasis exacerbation collected up to 24 months after visit 1: screening/baseline (used to extend modelling beyond 12 months as a sensitivity analysis).

For peer review only - http://bmjopen.bmj.com/site/about/guidelines.xhtml

	• Time to first exacerbation of bronchiectasis	• Time to first exacerbation of bronchiectasis
	• Number of emergency hospital admissions	• Number of emergency hospital admissions (all cause) during 12-month treatment period
	• Adverse events/drug reactions and cessation of treatment	• Number of adverse events/drug reactions and cessation of treatment 1-, 6- and 12-months visits.
	• Disease related health status using the St Georges Respiratory questionnaire (SGRQ) and Quality of Life Bronchiectasis (QOL-B) questionnaire	• SGRQ and QOL-B at baseline, 1-, 6- and 12-months visits
	• Health related quality of life using the EQ-5D-5L questionnaire	• EQ-5D-5L at baseline, 1-, 6- and 12-months visits
	• Breathlessness using Baseline and Transition Dyspnoea Indices (BDI and TDI)	• BDI at baseline and TDI at 1-, 6- and 12-months visits
	• Lung function using spirometry	Spirometry at baseline, 1-, 6- and 12-month visits:<br>• forced expiratory volume in 1 second (FEV1)<br>• forced vital capacity (FVC)
	• All-cause, respiratory and cardiac mortality	• Data from medical records and Office for National Statistics (ONS) records collected up to 24 months after visit 1: screening/baseline
	• Incremental cost per exacerbation avoided	• Costs based on cost of the interventions (micro-costed) and use of health services collected baseline, 1-, 6- and 12-months
	• Costs to the NHS and patients and lifetime cost-effectiveness based on extrapolation modelling	• Data to populate the model will come from the trial and will be supplemented by Hospital Episode Statistics (HES) and ONS data (collected up to 24 months after visit 1: screening/baseline) and by relevant literature and expert opinion and extrapolated over a patient lifetime
	• Rates of radiologically confirmed pneumonia, compared to participant's normal baseline	• Number of pneumonia events and total number of participants suffering pneumonia

For peer review only - http://bmjopen.bmj.com/site/about/guidelines.xhtml

**Exploratory**	• To investigate the relationship between key outcomes (exacerbations and quality of life as measured by SGRQ and QOL-B) with baseline eosinophil (single level recorded at baseline), median eosinophil level (median of last 3 available recording when not on oral steroids) and baseline BSI	• Eosinophil measurement at baseline and the median of the last 3 measurements available<br>• SGRQ and QOL-B as completed by participants at 1-, 6- and 12-month follow-up visits<br>• BSI measured at baseline<br>• The number of protocol defined bronchiectasis exacerbations requiring treatment with antibiotics during 12-month treatment period

Identification, consent and screening of patients

Potential participants aged 18 years or older with CT scan confirmed bronchiectasis, where bronchiectasis is the predominant primary respiratory disease, will be identified and recruited through secondary care NHS sites. Potential participants will be identified by clinicians and members of the research team from patients attending outpatient clinics, spirometry, oxygen services, in-patient admissions and by examination of databases such as local, BronchUK [19] and EMBARC [20] registries. Primary care practices may also be set up as Participant Identification Centres (PICs) to identify potential participants and signpost them to an appropriate secondary care recruitment site.

Potential participants will be approached in person or by post with a trial specific Patient Information Sheet (PIS) (Supplementary Information). Each potential participant will have time to read and consider the PIS before having an opportunity to discuss the trial further with a member of the research team. Written, informed consent will be obtained by a medically qualified member of the research team prior to any trial specific screening activity.

Screening of potential participants will involve:

- Collection of demographic data, medical history, medication history and smoking history
- Lung function as measured by spirometry
- Modified Reiff scoring [21] of the latest CT scan performed as part of standard care
- Quality of Life measurements including Quality of Life Bronchiectasis (QoL-B) questionnaire [22], St George's Respiratory Questionnaire (SGRQ) [4] and the 5-level EuroQol5D index (EQ-5D-5L) questionnaire [23]
- BSI Score [11]
- Assessments of breathlessness including the MRC Dyspnoea Score [24] and BDI and TDI respectively [25]

For peer review only - http://bmjopen.bmj.com/site/about/guidelines.xhtml

- Health Economics questionnaires
- Baseline bloods (full blood count including differentials)

All participants of childbearing must be willing to use an acceptable form of contraception (as listed in the PIS) throughout the trial from the date of consent until 7 days after their last dose of trial medication. Additionally, women of childbearing potential will be required to have a negative urine pregnancy test at baseline/screening to be eligible for the trial.

Randomisation

Patients who are confirmed as eligible will be randomised to one of three treatment arms dual therapy (LABA + LAMA), triple therapy (LABA + LAMA + ICS) or matched placebo, in a 2:2:1 ratio (respectively). Randomisation is carried out by appropriately delegated members of the research team using random permuted blocks of variable length within strata via the Sealed Envelope system; a central, secure, 24-hour web-based randomisation system with concealed allocation.

Randomisation is stratified by two variables: BSI score (BSI score of 0-8 or 9+) and by baseline ICS drug therapy (ICS user or non-ICS user at baseline).

Intervention

Treatment allocation is double-blind, each inhaler is identical in appearance to maintain the blind. Participants will have a dose of one inhalation of their trial inhaler per day, it is advised that the dose is taken at the same time of day wherever possible, for the 12-month (365 days) trial period. Dose modifications are not permitted.
The contents of each inhaler are described in ***Table 2***.

Table 2. Inhaler contents

INHALER	CONTENTS OF EACH DOSE DELIVERED	NAME OF EQUIVALENT COMMERCIALLY AVAILABLE PRODUCT
Dual therapy (LAMA/LABA)	55 micrograms umeclidinium<br>22 micrograms vilanterol	Anoro Ellipta dry powder inhaler
Triple therapy (ICS/LAMA/LABA)	92 micrograms fluticasone furoate<br>55 micrograms umeclidinium<br>22 micrograms vilanterol	Trelegy Ellipta dry powder inhaler
Placebo	placebo	Matched placebo dry powder inhaler

For peer review only - http://bmjopen.bmj.com/site/about/guidelines.xhtml

Each inhaler contains 30 doses of medication. Due to the shelf life of the labelled inhalers, they will be shipped to participants approximately monthly throughout their participation in the trial. Confirmation of receipt of trial medication will be carried out by a member of the research staff telephoning the patient following each trial medication shipment. Trial inhalers are returned by participants at the end of their involvement in the trial and compliance monitored.

Follow-up visits and participant assessments

Follow-up visits will take place at the local trial site at 1-, 6- and 12-months with assessments performed as detailed in Table 3.

Participants will be required to complete an Exacerbation Diary weekly throughout their 12-month participation in the trial. In this diary participants will record whether or not they have experienced any exacerbations during the week and any steps or treatment taken for the exacerbation. This will be reviewed by a member of the research team at each trial visit and collected at the final 12-month follow-up.

Long term follow-up will be carried out 24 months after the screening/baseline visit for participants who reach this time point within the lifetime of the trial. Data collected at this time point are mortality data, the number of emergency hospital admissions and the number of emergency hospital admissions with exacerbations of bronchiectasis. Participants will not be contacted for this follow-up, instead data will be collected from sources such as patient medical records, BronchUK [19] and/or EMBARC [20] registries or routinely collected Hospital Episode Statistics (HES) and Office of National Statistics (ONS) data.

At the end of their trial participation, participants will stop taking trial medication and continue to have their condition managed through the standard care pathway.

Table 3. Schedule of Events

**Assessment/Activity**	**Visit 1**	**Visit 2**	**Telephone call to participant**	**Visit 3**	**Telephone call to participant**	**Visit 4**	**Telephone call to participant**	**24 Month Follow up**
	**Screening / Baseline**	**1 Month Follow Up**		**6 Month Follow Up**		**12 Month Follow Up**		
	**Day 0**	**1 month (-1/+ 2 weeks)**	**3 days (+/- 1 day) following dispensing 2 to 6/7**	**6 months (-1/+2 weeks)**	**3 days (+/- 1 day) following dispensing 6/7 to 13**	**12 months (+2 weeks)**	**7 days after last dose of IMP (+ 3 days)**	
Written informed consent[a]	X							
Demographics	X							
Contact details (participant telephone number and address)	X	X	X	X	X			

For peer review only - http://bmjopen.bmj.com/site/about/guidelines.xhtml

Assessment/Activity	Visit 1	Visit 2	Telephone call to participant	Visit 3	Telephone call to participant	Visit 4	Telephone call to participant	24 Month Follow up
	Screening / Baseline	1 Month Follow Up		6 Month Follow Up		12 Month Follow Up		
	Day 0	1 month (-1/+ 2 weeks)	3 days (+/- 1 day) following dispensing 2 to 6/7	6 months (-1/+2 weeks)	3 days (+/- 1 day) following dispensing 6/7 to 13	12 months (+2 weeks)	7 days after last dose of IMP (+ 3 days)	
Medical history	X							
Medication history	X							
Smoking history	X	X		X		X		
Spirometry including % predicted (FEV1 & FVC)	X	X		X		X		
Bronchiectasis Severity Index calculation	X					X		
Modified Reiff scoring of prior CT scan	X							
St George's Respiratory Questionnaire (SGRQ)	X	X		X		X		
Quality of Life – Bronchiectasis (QOL-B)	X	X		X		X		
Breathlessness – Baseline and Transition Dyspnoea Indices (BDI and TDI)	X	X		X		X		
MRC Dyspnoea Score	X	X		X		X		
Healthcare Utilisation Questionnaire	X	X		X		X		
Time and Travel Questionnaire						X		
Quality of life EQ-5D-5L	X	X		X		X		
Baseline blood test (FBC with differentials)	X							
Pregnancy test (urine, females of childbearing potential)	X							
Contraception discussion	X							
GP results request/ prior eosinophil levels extracted from medical records/ primary care datasets	X							
Eligibility confirmation	X							
Randomisation	X							
Stop pre-randomisation inhalers with the exception of SABA and commence trial inhalers	X							
Trial inhaler delivered to participant on site at face to face visit	X							
Trial inhaler delivered to participant by post (2-4 days prior to each telephone call)			X	X	X			
Confirmation of trial inhaler receipt			X	X	X			
Inhaler technique	X	X		X				
Concomitant medication		X		X		X		
Issue Patient Exacerbation Diary	X							
Participant exacerbation diary review (or reminder to use at telephone calls)		X	X	X	X	X		

For peer review only

For peer review only - http://bmjopen.bmj.com/site/about/guidelines.xhtml

Assessment/Activity	Visit 1	Visit 2	Telephone call to participant	Visit 3	Telephone call to participant	Visit 4	Telephone call to participant	24 Month Follow up
	Screening / Baseline	1 Month Follow Up		6 Month Follow Up		12 Month Follow Up		
	Day 0	1 month (-1/+ 2 weeks)	3 days (+/- 1 day) following dispensing 2 to 6/7	6 months (-1/+2 weeks)	3 days (+/- 1 day) following dispensing 6/7 to 13	12 months (+2 weeks)	7 days after last dose of IMP (+ 3 days)	
Compliance check and documentation (count of returned trial inhalers and dose counts)		X		X		X		
Number of hospital admissions for bronchiectasis exacerbation		X		X		X		X
Number of emergency hospital admissions		X		X		X		X
Adverse event reporting		X	X	X	X	X	X	
Review of primary care records						X		
Mortality Data								X

For peer review only

Pharmacovigilance

All adverse events (AEs) will be recorded in both the participant's medical records and on the electronic Case Report Forms (eCRF) within the trial database. Adverse events that are judged by an investigator as consistent with the usual clinical pattern for patients with bronchiectasis (such as cough, increased sputum volume and/or consistency, change in sputum colour, wheeze, breathlessness, fatigue and haemoptysis) are not reportable AEs. AEs meeting the seriousness criteria (Serious Adverse Events [SAEs]) will be reported within 24 hours of awareness. Suspected Unexpected Serious Adverse Reactions (SUSAR) will be reported to the MHRA (Medicines and Healthcare products Regulatory Agency) and REC (Research Ethics Committee) within the required regulatory reporting timelines.

Emergency unblinding is available for valid medical or safety reasons where it is necessary for the treating clinician to know which treatment the participant has been receiving. Emergency unblinding should be carried out by the site Principal Investigator (PI) or another delegated medically qualified member of the research team by accessing the 24-hour web-based randomisation system.

Discontinuation and Withdrawal

Participants are free to discontinue trial treatment or withdraw from the trial at any time without detriment to their care. Participants choosing to discontinue trial treatment will be invited to complete all trial follow-up visits and assessments. Participants choosing to withdraw from the trial will not take part in any further trial activity. Data collected up to the point of withdrawal will be retained and

For peer review only - http://bmjopen.bmj.com/site/about/guidelines.xhtml

included in analysis. Participants withdrawn from the trial after they have been randomised will not be replaced.

Data Management

Participant data will be entered by delegated site staff into the trial-specific Clinical Data Management System (CDMS) using Sealed Envelope's Red Pill (Sealed Envelope Ltd, London, UK). CDMS users have password limited access restricted to own role and site as appropriate to their delegated duties. Each potential participant will be assigned a unique sequential screening number by site staff, this unique identifier is used to add the participant to the CDMS and becomes their participant ID at the time of randomisation. Overall responsibility for data collection, quality and retention lies with the Chief Investigator who will also hold the final trial dataset. Data will be handled, computerised, stored and archived in accordance with the General Data Protection Regulation (2018), and the latest Directive on GCP (2005/28/EC). Newcastle Clinical Trials Unit (NCTU) staff monitor trial conduct and data integrity in accordance with the trial Monitoring Plan. The trial specific Data Management Plan (DMP) and Data Validation Plan (DVP) include details on how data will be managed and validated throughout the trial.

Analysis

Analysis of the Primary Outcome Measure

The primary outcome - number of bronchiectasis exacerbations per participant requiring antibiotics over 12 months - will be compared between randomised groups using negative binomial regression adjusted for stratification factors. This model will be used to compute confidence intervals (CIs) to test the superiority and non-inferiority hypotheses. The two-sided 95% CI for difference in mean number of exacerbations per year between the combination of LAMA/LABA and ICS/LAMA/LABA compared to placebo will be found: the placebo vs LAMA/LABA hypothesis will be rejected if the upper limit is lower than 0. We will estimate the difference between LAMA/LABA and ICS/LAMA/LABA arms on the relative scale using the incidence rate ratio, and test whether the upper boundary of the two-sided 90% CI is lower than 1.2. We will consider the time at risk to be the time not spent in exacerbation (so that whilst a patient is in an exacerbation they are not included as at risk for another).

A secondary analysis will define the at-risk time as the entire length of follow-up for the patient. Estimates will then be adjusted for sites, the stratification factors and other baseline covariates that are known to be strongly related to outcome (e.g. age, smoking, bronchiectasis hospitalisations in year prior to trial – these will be pre-specified in the Statistical Analysis Plan (SAP)).

We will undertake a sensitivity analysis by excluding those participants who have died. If there is any indication of a differential effect on deaths by treatment, we may consider models that allow the

For peer review only - http://bmjopen.bmj.com/site/about/guidelines.xhtml

censoring to be informative. For participants that are lost to follow up by 12 months, their information will be included in the statistical models up to the point that they are lost to follow up. If loss to follow-up is higher than 10% we will conduct sensitivity analyses to investigate the impact.

We will also explore time to first exacerbation using a Cox regression, and a recurrent events analysis to allow for subsequent exacerbations. In addition, we will utilise mortality and hospitalisations due to bronchiectasis exacerbation data collected up until 24 months to extend the modelling beyond 12 months as a sensitivity analysis.

For the primary superiority hypothesis, statistical analyses will be according to the intention to treat principle with a per protocol analysis performed as a sensitivity analysis. The per protocol analysis will exclude participants who were not compliant (at less than 75%) with their trial medication or who had a major protocol violation (to be pre-specified in the SAP).

Analysis of Secondary Outcome Measures

The secondary outcomes: total number of bronchiectasis exacerbations requiring hospital admission, total number of emergency hospital admissions (all causes) will each be analysed as for the primary outcome described above. Disease related health status (measured using the SGRQ, FEV1 and FVC) will be analysed using a mixed effects model adjusted for site, stratification factors, patient characteristics and/or baseline clinical variables (to be pre-specified in the Statistical Analysis Plan). Random effects for patient will be included.

Exploratory and Subgroup Analyses

Exploratory analyses will investigate the relationship between key outcomes (exacerbations and quality of life as measured by SGRQ and QOL-B) with baseline eosinophils (single level recorded at baseline), median eosinophil level (median of last 3 available recordings when not on oral steroids) and baseline BSI.

Subgroup analyses of suspected aetiology comparing idiopathic and post infectious to all other aetiologies for the key outcomes of exacerbations and quality of life (SGRQ and QOL-B) will also be carried out.

All analyses will be governed by this comprehensive SAP which will be agreed by the TSC and reviewed by the IDMEC prior to any analyses being undertaken. Unless pre-specified, a 5% two-sided significance level will be used to denote statistical significance throughout.

Economic Evaluation

For peer review only - http://bmjopen.bmj.com/site/about/guidelines.xhtml

Both a within-trial and model-based analysis will be undertaken. Both analyses will estimate the incremental cost per QALY gained but over different time horizons; 12-months post-randomisation (within-trial) and over the patients' life course (economic model).

The economic analysis will take the perspective of the NHS and personal and social services (PSS). Sensitivity analyses will widen the perspective to incorporate costs incurred by participants. Intervention costs will be estimated based on the manufacturers list price of the medications and inhalers provided to participants. Primary and secondary healthcare resource use will be estimated based on responses to the Health Care Utilisation Questionnaire (HCUQ) administered at baseline, 1-, 6-, and 12-months post-randomisation. These data will be combined with unit costs obtained from routine sources to estimate the total healthcare utilisation cost per participant. Additionally, patients with bronchiectasis are likely to experience AEs, treatments and hospitalisations associated with these AEs will be recorded on the eCRF and will be incorporated into the total healthcare utilisation cost per participant in a sensitivity analysis.

Direct (e.g. out-of-pocket payments) and indirect (e.g. time away from work) costs borne by participants will be collected via the Time and Travel questionnaire administered at 12-months post-randomisation. Similar to the healthcare costs these study specific estimates will be combined with routine sources to estimate the total costs incurred for each participant.

Health-related quality of life will be assessed based on responses to the EQ-5D-5L administered at baseline, 1-, 6-, and 12-months post-randomisation. Utility scores will be derived based on responses to the EQ-5D-5L questionnaire and the national tariff relevant at the time the study reports. QALYs will be estimated using the utility values estimated at baseline, 1-, 6-, and 12-months using the area under the curve method [26].

The total cost and QALY per participant will be summarised as the average total cost and average total QALY for each of the randomised arms. All data will be presented as point estimates of incremental costs, QALYs, cost-effectiveness. The incremental cost per QALY gained will be estimated as the difference in costs divided by the difference in QALYs if one of the arms is not dominant (i.e. less costly and more effective). The difference in costs and QALYs between the arms will be estimated using seemingly unrelated regression which can control for observed and unobserved characteristics that can affect costs and/or QALYs [27]. Uncertainty in these results, estimated using the bootstrapping technique [28], will be presented on cost-effectiveness planes and cost-effectiveness acceptability curves (CEACs).

For peer review only - http://bmjopen.bmj.com/site/about/guidelines.xhtml

Given that patients with bronchiectasis are expected to take their medications over their life horizon an economic model will be used to extrapolate the economic results beyond the 12-month treatment period. It is likely that this model will take the form of a Markov model but the specific form will be decided based on the within-trial results during the model development. Data to design and populate the model will be taken directly from the trial, routine sources (ONS and HES data), literature and clinical opinion. Cost and QALYs incurred after the first 12-months will be discounted at the recommended rate [29]. The model will be developed using the guidance for good practice in conceptualising an economic model [30]. Similar to the within-trial analysis these results will be presented as point estimates of costs, QALYs and cost-effectiveness. Uncertainty in the economic model will be estimated using monte carlo simulations and presented as cost-effectiveness planes and CEACs [31, 32].

Sample Size Calculations

The original sample size calculation was based on the number of exacerbations amongst a similar population in prior national audits [15, 23]. The average number of exacerbations was 2.3 per year. Restricting trial entry to those who had >=3, the original inclusion criteria, (assuming a Poisson distribution) gives an average of around 3.8. We therefore assumed, given Hawthorne effect and regression to the mean that the placebo arm would have a lower mean exacerbation rate than this, and assumed a mean of 2.4 over 1 year. The sample size was chosen so that the trial was well powered to detect a clinically meaningful fall in mean exacerbation rates for bronchiectasis exacerbations to 1.9 year in LAMA/LABA and ICS/LAMA/LABA arms (approximately 20% reduction). This effect size is realistic when compared with the 20-30% reduction seen in COPD trials with dual bronchodilators/ triple therapy and is accepted as clinically meaningful. Although likely studying a different sub population in bronchiectasis, a recent inhaled antibiotic trial in bronchiectasis reduced exacerbations by 39% [4].

For 90% power (two-sided 5% significance level) to conclude that LAMA/LABA is more effective than placebo with the above parameters, we have calculated (assuming large-sample approximation of the Poisson distribution) a sample size of 600 participants is needed, randomised 240:240:120 between LAMA/LABA, ICS/LAMA/LABA and placebo. This allows for a 5% loss to follow-up. This represents a conservative retention rate compared to over 95% observed in the NIHR HTA TWICS study with similar pragmatic design and limited patient burden. This calculation assumes a difference between placebo and LABA/LAMA of 0.5 exacerbations per year (21% relative reduction).

For peer review only - http://bmjopen.bmj.com/site/about/guidelines.xhtml

If superiority of LAMA/LABA vs placebo is concluded, we will then test non-inferiority of LAMA/LABA against ICS/LAMA/LABA. The sample size will give 90% power (one-sided 5% type I error) with a 0.38 non-inferiority margin (reflecting 20% of the assumed LAMA/LABA rate).

In the early stages of the trial it was found that exacerbation rates in pre-baseline periods were lower than they had been historically, mostly due to the presumed effect of COVID-19 restrictions. The inclusion criteria were therefore updated in protocol V5.0 (see Amendments). A recalculation of the power of the trial was conducted assuming that there would be lower exacerbation rates in the follow-up period. We assumed that the placebo exacerbation rate would be an average of 1.9 per year. We recalculated the power for two scenarios: 1) assuming that the same absolute difference (0.5) between LAMA/LABA vs placebo and the same non-inferiority margin (0.38) as previously; 2) the same relative difference (21%) between LAMA/LABA vs placebo and relative non-inferiority margin (20%) as before. Table 4 shows the power of the trial to conclude non-inferiority and superiority.

Table 4. Power Calculations

**Scenario**	**Mean exacerbation rate**			**NI Margin (relative to active control)**	**Power***	
	**Placebo**	**ICS/LAMA/LABA (Intervention)**	**LAMA/LABA (Active control)**		**Non-inferiority**	**Superiority**
Presented in original grant application	2.4	1.9	1.9	0.38 (20%)	90.3%	89.8%
Updated inclusion criteria (same absolute differences)	1.9	1.4	1.4	0.38 (26.7%)	96.3%	95.8%
Updated inclusion criteria (same relative differences)	1.9	1.5	1.5	0.3 (20%)	83.4%	83.4%

For peer review only - http://bmjopen.bmj.com/site/about/guidelines.xhtml

*Note this is assuming analytical formulae, with simulations giving consistent but slightly higher powers

Amendments

A number of amendments have been approved for the trial. Of note is Amendment 06, a substantial amendment for which the primary purpose was to change eligibility criteria and to extend the pilot phase of the trial.

Initially the protocol required that patients experience 3 exacerbations of bronchiectasis within the preceding 12 months to be eligible for the trial. However, this criterion posed a challenge to recruitment given that the biology of bronchiectasis has changed following the shielding behaviour of patients with bronchiectasis during the COVID-19 pandemic. These behaviours have resulted in a reduction in exacerbations being observed in the U.K. [33] and hence, a reduction in the pool of potentially participants for the trial. In discussion with the oversight committees and funder for the trial it was agreed to extend the potential participant pool for the trial by changing this eligibility criterion to include patients who have experienced 2 or more exacerbations within any 12 month period at any time in the preceding two years.

As a reflection of the slower recruitment and the challenges faced by sites setting up a respiratory trial during COVID-19, it was agreed to extend the pilot phase of the trial from 6 to 12 months.

Patient and Public Involvement (PPI)

PPI were involved in the trial design and are co-applicants for the research. Independent PPI have oversight of the trial and results dissemination as members of the TSC.

Trial Status

The DIBS trial recruitment opened on the 29th July 2021 and closed on the 21st October 2022, the last patient last visit will be in October 2023. This manuscript is based in protocol version 6.0 dated 15th July 2022.

ETHICS AND DISSEMINATION

The trial received a favourable ethical opinion from the North East – Newcastle & North Tyneside 2 Research Ethics Committee (reference: 21/NE/0020) in March 2021. Trial results will be disseminated in peer reviewed publications, at national and international academic conferences and in the NIHR Health Technology Assessments (HTA) journal. Results will also be disseminated to the public and participants using lay language.

For peer review only - http://bmjopen.bmj.com/site/about/guidelines.xhtml

] **AUTHOR CONTRIBUTIONS**

ADS is the trial Chief Investigator and senior author. AS, RM, CH, JH, ADS prepared the funding application. All authors contributed to protocol development and amendments and have revised the manuscript and given approval for the final version. NW, JW and SC have advised on trial design and provide statistical oversight and analysis. TH and LT have advised on trial design and provide health economic oversight and analysis. MA and AW provide IMP oversight, MM, LS, AS, RM, AA, RJ, VH and RL provide trial and database design and management and trial monitoring.

FUNDING STATEMENT

This work was supported by the National Institute for Health Research (NIHR) Health Technology Assessment (HTA), funder reference NIHR127460. The views expressed are those of the author(s) and not necessarily those of the NIHR or the Department of Health and Social Care.

The inhalers studies within the main study have been provided by Glaxo Smith Kline as part of an unrestricted medical education grant. The company have not played a role in the study design or conduct.

COMPETING INTERESTS STATEMENT

ADS has received speakers fees or advisory board fees from AstraZeneca, Bayer, GSK, Insmed, Novartis, Gilead and Zambon and has received grants from AstraZeneca, GSK, Novartis and the US COPD Foundation**.**

FULL REFERENCES

1. Pasteur, M.C., et al., *An investigation into causative factors in patients with bronchiectasis.* Am J Respir Crit Care Med, 2000. **162**(4 Pt 1): p. 1277-84.
2. Shoemark, A., L. Ozerovitch, and R. Wilson, *Aetiology in adult patients with bronchiectasis.* Respir Med, 2007. **101**(6): p. 1163-70.
3. O'Leary, C.J., et al., *Relationship between psychological well-being and lung health status in patients with bronchiectasis.* Respir Med, 2002. **96**(9): p. 686-92.
4. Wilson, C.B., et al., *Validation of the St. George's Respiratory Questionnaire in bronchiectasis.* Am J Respir Crit Care Med, 1997. **156**(2 Pt 1): p. 536-41.
5. De-Soyza, A., et al. *Earlier onset of fatigue in bronchiectasis as compared with COPD*. in *European Respiratory Society Annual Congress*. 2010. Newcastle University.
6. Hester, K.L., et al., *Fatigue in bronchiectasis.* Qjm, 2012. **105**(3): p. 235-40.
7. Patel, I.S., et al., *Bronchiectasis, exacerbation indices, and inflammation in chronic obstructive pulmonary disease.* Am J Respir Crit Care Med, 2004. **170**(4): p. 400-7.
8. Shahab, L., et al., *Prevalence, diagnosis and relation to tobacco dependence of chronic obstructive pulmonary disease in a nationally representative population sample.* Thorax, 2006. **61**(12): p. 1043-7.
9. Roberts, H.J. and R. Hubbard, *Trends in bronchiectasis mortality in England and Wales.* Respir Med, 2010. **104**(7): p. 981-5.

For peer review only - http://bmjopen.bmj.com/site/about/guidelines.xhtml

For peer review only

10. Loebinger, M.R., et al., *Mortality in bronchiectasis: a long-term study assessing the factors influencing survival.* Eur Respir J, 2009. **34**(4): p. 843-9.
11. Chalmers, J.D., et al., *The bronchiectasis severity index. An international derivation and validation study.* Am J Respir Crit Care Med, 2014. **189**(5): p. 576-85.
12. Seitz, A.E., et al., *Trends and burden of bronchiectasis-associated hospitalizations in the United States, 1993-2006.* Chest, 2010. **138**(4): p. 944-9.
13. Quint, J.K., et al., *Changes in the incidence, prevalence and mortality of bronchiectasis in the UK from 2004 to 2013: a population-based cohort study.* Eur Respir J, 2016. **47**(1): p. 186-93.
14. Kelly, M.G., S. Murphy, and J.S. Elborn, *Bronchiectasis in secondary care: a comprehensive profile of a neglected disease.* Eur J Intern Med, 2003. **14**(8): p. 488-492.
15. Hill, A.T., et al., *British Thoracic Society national bronchiectasis audit 2010 and 2011.* Thorax, 2012. **67**(10): p. 928-30.
16. *Global Initiative for Asthma. Global Strategy for Asthma Management and Prevention. Available from:* www.ginasthma.org. 2022.
17. *Global Initiative for Chronic Obstructive Lung Disease - goldcopd.org*. 2022.
18. Chan, A.W., et al., *SPIRIT 2013 explanation and elaboration: guidance for protocols of clinical trials.* Bmj, 2013. **346**: p. e7586.
19. De Soyza, A., et al., *BronchUK: protocol for an observational cohort study and biobank in bronchiectasis.* ERJ Open Res, 2021. **7**(2).
20. Chalmers, J.D., et al., *The European Multicentre Bronchiectasis Audit and Research Collaboration (EMBARC): experiences from a successful ERS Clinical Research Collaboration.* Breathe (Sheff), 2017. **13**(3): p. 180-192.
21. Reiff, D.B., et al., *CT findings in bronchiectasis: limited value in distinguishing between idiopathic and specific types.* AJR Am J Roentgenol, 1995. **165**(2): p. 261-7.
22. Quittner, A.L., et al., *A preliminary quality of life questionnaire-bronchiectasis: a patient-reported outcome measure for bronchiectasis.* Chest, 2014. **146**(2): p. 437-448.
23. Devlin, N.J., et al., *Valuing health-related quality of life: An EQ-5D-5L value set for England.* Health Econ, 2018. **27**(1): p. 7-22.
24. *Standardized Questionaries on Respiratory Symptoms.* Br Med J, 1960. **2**(5213): p. 1665.
25. Mahler, D.A., et al., *The measurement of dyspnea. Contents, interobserver agreement, and physiologic correlates of two new clinical indexes.* Chest, 1984. **85**(6): p. 751-8.
26. Matthews, J.N., et al., *Analysis of serial measurements in medical research.* Bmj, 1990. **300**(6719): p. 230-5.
27. Fiebig, D.G., *A Companion to Theoretical Econometrics*. Seemingly Unrealted Regression., ed. B.H. Baltagi. 2003, Malden, MA, USA: Blackwell Publishing Ltd.
28. Barber, J.A. and S.G. Thompson, *Analysis of cost data in randomized trials: an application of the non-parametric bootstrap.* Stat Med, 2000. **19**(23): p. 3219-36.
29. National Institute for, H. and E. Care, *NICE Process and Methods Guides*, in *Guide to the Methods of Technology Appraisal 2013*. 2013, National Institute for Health and Care Excellence (NICE): London.
30. Philips, Z., et al., *Review of guidelines for good practice in decision-analytic modelling in health technology assessment.* Health Technol Assess, 2004. **8**(36): p. iii-iv, ix-xi, 1-158.
31. Black, W.C., *The CE plane: a graphic representation of cost-effectiveness.* Med Decis Making, 1990. **10**(3): p. 212-4.
32. Fenwick, E., K. Claxton, and M. Sculpher, *Representing uncertainty: the role of cost-effectiveness acceptability curves.* Health Econ, 2001. **10**(8): p. 779-87.
33. Crichton, M.L., A. Shoemark, and J.D. Chalmers, *The Impact of the COVID-19 Pandemic on Exacerbations and Symptoms in Bronchiectasis: A Prospective Study.* Am J Respir Crit Care Med, 2021. **204**(7): p. 857-859.

For peer review only - http://bmjopen.bmj.com/site/about/guidelines.xhtml

For peer review only

For peer review only - http://bmjopen.bmj.com/site/about/guidelines.xhtml

TO BE PRINTED ON TRUST HEADED PAPER

Dual Bronchodilators in Bronchiectasis Study

DIBS

Participant Information Sheet

Version 4.0: 18th February 2022

INVITATION

We are inviting you to take part in our research study called DIBS. Please read the following information to help you decide if you want to take part. We would like you to understand why this research is being done and what it would involve for you. You don't have to decide straight away and you can talk to your friends and family or GP. A member of our team will go through this information sheet with you and answer any questions you may have. The study is being funded by the National Institute for Health Research (NIHR) which is the Health Research arm of the NHS.

TRIAL SUMMARY

- This study is looking at the use of bronchodilator inhalers for preventing exacerbations of bronchiectasis. Bronchodilators are a type of medication taken that relax the muscles in the lungs and widen the airways to make breathing easier. The inhalers being tested are a dual bronchodilator inhaler and a dual bronchodilator combined with corticosteroid inhaler (corticosteroid inhalers are also sometimes referred to as steroid inhalers). You will stop taking any inhaler(s) you are already on with the exception of your quick-acting inhaler (such as salbutamol). You will be randomised to either one of the two treatment inhalers or the placebo inhaler (a 'dummy' treatment). You will have four times the chance of

For peer review only - http://bmjopen.bmj.com/site/about/guidelines.xhtml

being on either one of the treatment inhalers as on the placebo inhaler. You and the study doctor will not know which inhaler you are taking.

- You will use your study inhaler once a day for 12 months (365 days).
- You will have 4 hospital visits for the study over the 12 months. These visits will take place alongside your normal hospital appointments or as additional visits. During these visits you will have some tests and complete some questionnaires.
- You will have a telephone call approximately every month to check that you have received your replacement inhaler through the post.
- You will be asked to complete an exacerbation diary each week for 12 months to record any bronchiectasis exacerbations.
- The study will include 600 bronchiectasis patients at over 20 hospitals.
- Your participation is voluntary, you will be part of a national effort to better understand bronchiectasis and its treatment.

Please read the following information for further details about the study if you are interested in taking part.

WHY IS DIBS NEEDED?

There are over 300,000 patients in the UK who suffer from bronchiectasis. There are currently no licensed treatments and no cures, which means there are large differences in care. Patients with bronchiectasis are normally treated with inhaled medicines approved for asthma and smoking-related chronic obstructive pulmonary disease (COPD) without any proof they work in bronchiectasis. We do not know which inhaled treatment is best for bronchiectasis; this is why we are conducting this research study. We will look at whether a dual bronchodilator inhaler or a dual bronchodilator plus a corticosteroid inhaler can reduce the number of bronchiectasis exacerbations (or flare ups) requiring antibiotic or steroid treatment compared to no treatment. An exacerbation (or flare up) is a sustained deterioration in your condition that would lead you to think about seeing your healthcare team or starting emergency packs.

For peer review only - http://bmjopen.bmj.com/site/about/guidelines.xhtml

The safety and side effects of these inhalers in other respiratory conditions are well known.

WHY AM I BEING INVITED TO TAKE PART?

You are being invited to take part in this research study because you have been diagnosed with a respiratory condition called bronchiectasis. You are also being invited because within the last 2 years you have had 2 or more exacerbations (or flare ups) within 12 months of each other which have required antibiotic or steroid treatment.

DO I HAVE TO TAKE PART?

No, it is up to you to decide whether you want to join the study. If you agree to take part, we will ask you to complete a consent form. If you choose not to, you will continue to get the standard care arranged by your doctor.

If you agree to take part, you can change your mind at any time. You can stop taking study medication and carry on with the questionnaires and study visits, or you can withdraw from the study completely. You do not have to give a reason, but it is helpful to the study if you do so ways it can be improved can be understood. If you decide to withdraw completely, data collected up to this point will be retained for analysis. This will not affect the care that you receive.

WHAT WILL GIVING CONSENT MEAN FOR ME?

By signing a consent form, this means that you fully understand what taking part in the study means for you. That's why it is really important that you take as much time as you want to read this information sheet and ask lots of questions.

WHAT WOULD TAKING PART INVOLVE?

We will ask you to attend 4 hospital visits:

- Initial visit
- 1 month follow-up visit
- 6 month follow-up visit
- 12 month follow-up visit

For peer review only - http://bmjopen.bmj.com/site/about/guidelines.xhtml

These visits are not part of your standard care but could take place alongside your standard care visits if it is possible. The diagram on page 66 shows the details of the study hospital visits and what will take place at each visit.

During your study hospital visits you will be asked to complete some questionnaires. Some will be completed by you and others will be completed by a member of the study team who will ask you questions. These questionnaires are used to assess any breathlessness you may have, the impact of your health on your usual activities, your mental health and quality of life. There are also questionnaires about how often you have had to access healthcare for routine appointments and urgent care, any costs you have incurred accessing healthcare and time taken to travel to access healthcare. We will use this to compare the overall healthcare costs between each treatment arm in the study.

A blood sample and urine sample (if needed for a pregnancy test) will be taken at the first (baseline) study visit. The blood sample is taken to look at reasons why patients may respond differently to the inhalers and as a baseline test for safety reasons. We will collect approximately 1 teaspoon (5 mL) full of blood. These samples will be analysed in the local hospital laboratory and will be destroyed after analysis. No samples (blood or urine) will be stored for the purposes of this study.

All of your study visits and procedures will be carried out in line with your study hospital's COVID-19 policies and procedures. These will be explained to you when your appointments are arranged.

For the 12 months of the study you will be asked to complete an exacerbation diary each week. You will record any exacerbations of your bronchiectasis in the last week and any antibiotic or steroid treatment.

We will arrange delivery of your inhalers in between the face to face visits, at a time convenient for you. These will be delivered by post or courier, depending on your local research team's policy.

You will have phone calls approximately every month with a member of the research team to check:

- That you have received your replacement inhaler in the post

For peer review only - http://bmjopen.bmj.com/site/about/guidelines.xhtml

- The current dose count on your inhaler
- Whether your contact details have changed
- Whether you have any planned travel/holidays that may affect the timing of the subsequent inhaler postings
- Completion of your exacerbation diary each week
- Whether you have had any adverse events (new or worsened signs or symptoms) or any hospital admissions

You will have to stop taking any current inhalers, with the exception if you have a blue (Salbutamol) inhaler used to relieve a sudden attack of breathlessness or wheezing. If you get this sort of attack you must continue to use a quick-acting inhaler (such as salbutamol).

You will have a phone call between 7 and 10 days after your last dose of the study inhaler. This call is to check whether you have had any adverse events (new or worsened signs or symptoms) or any hospital admissions since your most recent study visit.

We are interested in the long-term health of participants in this study. With your permission we will continue to ask for information relating to your health following the end of your participation in the study for up to one year (referred to in the study as 24 month follow up data), depending on the date you join the study. You will not need to visit hospital or be called by the research team for this. Instead, we will ask for information from Bronchiectasis Registries (BronchUK [The United Kingdom Bronchiectasis Registry] and EMBARC [European Multicentre Bronchiectasis Audit and Research Collaboration]) if you have already given consent to these projects for health follow ups. Or, we may ask for information from routinely collected sources such as NHS Digital (including Hospital Episodes Statistics [HES]) and the Office for National Statistics (ONS) (ONS/HES data will not be collected for participants with trial visits at Scottish hospitals). Members of the hospital research team may also review your medical records. The data collected will only include information about you for up to one year after your last dose of study medication, but, because data is requested in batches from HES, ONS and other databases, the request for your data may be made up to two years after your participation in the study has ended.

For peer review only - http://bmjopen.bmj.com/site/about/guidelines.xhtml

Figure 1. Hospital visit details

SCREENING/BASELINE VISIT – VISIT LENGTH 2-3 HOURS

- Written informed consent
- Eligibility for the trial confirmed
- Medical history, smoking history and medication review
- Urine pregnancy test (if needed)
- Blood tests
- Questionnaires
- Lung function tests
- Randomisation to treatment arm
- Receive first inhaler
- Test of inhaler technique

1 AND 6 MONTH FOLLOW UP – VISIT LENGTH 1-2 HOURS

- Check contact details
- Medication and smoking review
- Lung function tests
- Questionnaires
- Test of inhaler technique and used inhaler return
- Review of exacerbation diary and any adverse events including hospital visits

12 MONTH FOLLOW UP – VISIT LENGTH 1-2 HOURS

- Medication and smoking review
- Lung function tests
- Questionnaires
- Used inhaler return
- Review of exacerbation diary and any adverse events including hospital visits

For peer review only - http://bmjopen.bmj.com/site/about/guidelines.xhtml

WHAT TREATMENT WOULD I BE ON?

The active treatments we are testing have already been tested and shown to work in the smoking related lung condition called COPD. The inhalers are already in wide use across the UK for that condition. If you decide to take part in the study, you will be randomised by computer to one of three treatment groups: each arm has ONLY one inhalation from one inhaler per day.

- Dual inhaler (dual bronchodilator; Umeclidinium/Vilanterol)
- Triple inhaler (dual bronchodilator plus a corticosteroid; Umeclidinium/ Vilanterol & Fluticasone Furoate)
- Placebo inhaler (a 'dummy' treatment)

The dual inhaler contains 2 active medicines: Umeclidinium and Vilanterol; these both belong to a group of medicines called bronchodilators. Bronchodilators widen the muscles around the airways in the lungs which makes it easier for air to get in and out of the lungs. When used regularly, bronchodilators can help to control breathing difficulties.

The triple inhaler contains 3 active medicines: Umeclidinium, Vilanterol and Fluticasone Furoate. Umeclidinium and Vilanterol are the same bronchodilator medicines that are in the dual inhaler. The Fluticasone Furoate is a steroid medicine. Bronchodilators widen the muscles in the lungs and steroids reduce swelling and irritation in the small air passages which makes it easier for air to get in and out of the lungs. When used regularly bronchodilators and steroids can help to control breathing difficulties.

The placebo inhaler does not contain any active medicines; it contains a dry powder that should not have any effect on you.

The dual and triple inhalers have already been tested in large research studies for safety, side effects, and effectiveness in treating COPD and have been licenced for use in the treatment of COPD. The DIBS study is a phase 3 clinical trial. A phase 3 clinical trial is a study which includes a large number of patients to look at whether a treatment works well. We hope to see in DIBS whether bronchodilators and/or bronchodilators and steroids, which have been used already to treat COPD, are effective treatments for patients with bronchiectasis.

For peer review only - http://bmjopen.bmj.com/site/about/guidelines.xhtml

For peer review only

The inhalers all look the same. You will have four times the chance of getting an active inhaler as being in the placebo group (i.e. if there are 5 participants, 2 will have the dual inhaler, 2 will have the triple inhaler, and 1 will have the placebo inhaler).

Which inhaler you are given will be completely random. You and the healthcare professionals involved in the study will not know which type of inhaler you are allocated to.

You will be prescribed study inhalers for 12 months (365 days in total). Each inhaler has 30 doses and you need to take one puff of your inhaler every day for 12 months (365 days in total). You should try to take your inhaler at the same time every day. You will be given your first inhaler at your screening/baseline hospital visit and then receive replacement inhalers by post. The inhalers will be sent by recorded delivery and you may need to sign to confirm you have received them. On study visit days, do **not** use your inhaler before you arrive in hospital. You will be asked to bring your current inhaler to hospital and to take your daily dose whilst attending hospital. This is to check whether you are using your inhaler correctly.

PLEASE KEEP ALL OF YOUR USED INHALERS

It is vitally important that you keep hold of all your study inhalers, even the empty ones. You will be asked to return all inhalers at your next study visit so that the number of doses you have used can be checked and recorded.

WHAT ARE THE POSSIBLE BENEFITS OF TAKING PART?

Patients with other lung conditions where these inhalers are already proven to work reported better lung function, better quality of life and less exacerbations (or flare ups). We cannot promise this study will help you directly but the information we get from this study may help to improve treatment for people with bronchiectasis in the future. If you want to find out more about taking part in research studies you can visit the NHS Choices website https://www.nhs.uk/conditions/clinical-trials/

For peer review only - http://bmjopen.bmj.com/site/about/guidelines.xhtml

EXPENSES

Your travel expenses (up to £25 per visit) for each hospital visit will be paid for by the study. Alternatively, transport may be arranged for you if your local hospital is able to offer this. In addition, should you prefer to contact the study team for your monthly telephone call rather than the study team telephoning you, the costs of these telephone calls will be paid for by the study.

Your study team will manage any payments to reimburse costs to you and you may be asked to provide receipts for your travel.

WILL MY GP KNOW THAT I'M TAKING PART IN THE STUDY?

Yes, we will send a letter to your GP to inform them that you are taking part in this study, and a copy will be filed in your hospital notes. This is so that your medical records at your GP practice and in hospital contain documentation that you are taking part in a clinical study. Any test results from taking part in this study will also be added to your medical records. Your GP will be asked to inform the study team of any adverse reactions to the study medication or emergency hospital admissions.

Your GP will not know if you have received active treatment or placebo.

WHAT ARE THE POSSIBLE DISADVANTAGES AND RISKS OF TAKING PART?

We want you to be safe in this study at all times, but all medical treatments carry some risk. The inhalers used in this study are widely used by NHS patients with respiratory conditions, and there are some known side effects.

If you react badly to the inhaler your study doctor will be able to change your medication and treat you to try to ease your symptoms. If they need to find out which treatment you are taking (one of the two active treatments or placebo), this information is available in cases of emergency.

If you feel unwell during your time in the study then please contact your research team or study doctor.

POSSIBLE SIDE EFFECTS

For peer review only - http://bmjopen.bmj.com/site/about/guidelines.xhtml

As with most medicines the study inhalers do have some side effects which are listed in the table below. As these drugs are widely used in COPD our experience is that very few of these side effects are common causes for patients to stop their inhalers. If you are assigned to the triple inhaler group there may be a higher risk of you getting pneumonia. This is because the steroid inhalers have been shown to make the risk of getting pneumonia higher in patients with COPD. Pneumonia is when there is swelling of the tissue in one or both lungs, usually caused by an infection. The steroid inhaler reduces COPD exacerbations a lot more than the risk of pneumonia it brings to these patients. If you experience any of the side effects listed in the table below, it is important that you let your study team know straight away. The study team can talk to you more about what these mean if you are unsure about any of them. These side effects often are temporary.

Frequency	Dual inhaler (dual bronchodilator)	Triple inhaler (dual bronchodilator and corticosteroid)
**Common or very common**	▪ Increased risk of infection<br>▪ Cough<br>▪ Throat pain<br>▪ Constipation<br>▪ Dry mouth<br>▪ Headache	▪ Increased risk of infection<br>▪ Headache<br>▪ Cough<br>▪ Throat pain<br>▪ Constipation<br>▪ Joint pain<br>▪ Back pain<br>▪ Thrush in the mouth and throat
**Uncommon**	▪ Rash<br>▪ Tremor<br>▪ Altered taste<br>▪ Abnormal heart rhythms<br>▪ Palpitations<br>▪ Hoarse voice	▪ Abnormal heart rhythms<br>▪ Dry mouth<br>▪ Bone fracture<br>▪ Altered taste<br>▪ Hoarse voice<br>▪ Chest infection
**Rare or very rare**	▪ Swelling<br>▪ Anaphylaxis<br>▪ Blurred vision<br>▪ Glaucoma	▪ Swelling<br>▪ Anaphylaxis<br>▪ Skin rash

For peer review only - http://bmjopen.bmj.com/site/about/guidelines.xhtml

	▪ Pressure inside the eye<br>▪ Tightening of air passages (sometimes severe)<br>▪ Urinary problems	
**Frequency unknown***	▪ Dizziness	▪ Blurred vision

* the frequency of these side effects are unknown as there is not enough data available at the moment to estimate the frequency.

You could be randomised to receive the placebo 'dummy' treatment. If you get the placebo treatment it could be the case that you experience more exacerbations than on your current treatment/inhaler. You and the study team will not know which treatment you are receiving.

PREGNANCY AND CONTRACEPTION

As we do not know if the study medicines will harm an unborn baby those planning to become pregnant (or their partner becoming pregnant) will be excluded from the study.

To prevent pregnancy during the study, all participants of childbearing potential must use contraception from the day that consent is given to take part in the study up until 7 days after the last dose of study medication is taken.

For male participants contraceptive methods include:

- condom
- practice true abstinence in line with preferred and usual lifestyle

For female participants contraceptive methods include:

- combined hormonal contraception (oral, intravaginal, transdermal)
- progestogen only hormonal contraception (oral, injectable, implantable)
- intrauterine device (IUD)
- intrauterine hormone-releasing system (IUS)
- vasectomised partner
- bilateral tubal occlusion

For peer review only - http://bmjopen.bmj.com/site/about/guidelines.xhtml

- practice true abstinence in line with preferred and usual lifestyle

If you or your partner become pregnant during the course of the study, you must tell your study doctor **immediately** so appropriate action can be discussed.

If you do become pregnant during the study, you must stop taking your study inhaler. We will ask you to return your study inhaler at your next follow-up visit.

We will monitor your pregnancy carefully and will ask if we can collect some information on the health of your baby when it is born. A children's doctor will also be asked to check your baby carefully when it is born.

If your partner becomes pregnant during the study, we will ask them to sign a consent form. This will allow the study team to collect safety information about their pregnancy and their baby.

BREASTFEEDING

If you are breastfeeding you will not be allowed to take part in the study. This is because we don't know if the study medicine is passed into breastmilk or if it is safe in babies.

WHAT WILL HAPPEN TO ME WHEN THE STUDY ENDS?

You will stop taking the study inhalers and you will continue to receive standard care like any other patient with your condition, under the care of your respiratory doctor. You and your respiratory doctor can discuss which treatment would be best for you at that time. You and your doctors will not find out which inhaler you were taking during the study.

WHAT IF SOMETHING GOES WRONG?

If you have a concern about any aspect of this study, you can speak to a member of the study team who will do their best to answer your questions. Further contact details are included at the end of this information sheet. If you are still unhappy and wish to raise your concerns with someone who is not directly involved in your care, you can contact <site to localise with local details such as PALS (or equivalent) phone number and email address>

In the unlikely event that you are harmed during the study and this is due to someone's negligence (they were careless) you may have grounds for legal

For peer review only - http://bmjopen.bmj.com/site/about/guidelines.xhtml

action and compensation, but you may need to meet your own legal costs. NHS Indemnity does not offer no-fault compensation (for harm that is not anyone's fault).

The Newcastle Clinical Trials Unit, part of Newcastle University, are managing the study on behalf of the study NHS Sponsor. Newcastle University also have insurance arrangements in place to cover Newcastle University staff involved in designing and managing the DIBS study.

WHO IS ORGANISING AND FUNDING THE STUDY?

The doctor in charge of the study (the Chief Investigator) is Professor Anthony De Soyza, a Respiratory Consultant. He is based in Newcastle upon Tyne.

Study Sponsor: Newcastle upon Tyne Hospitals NHS Foundation Trust. The study Sponsor has responsibility for the study. The study is managed by the Newcastle Clinical Trials Unit, Newcastle University, on behalf of the Sponsor.

Study Funder: National Institute for Health Research Health Technology Assessment programme. This body is funded by the UK government to carry out research for the benefit of the NHS and its patients.

GlaxoSmithKline plc, a pharmaceutical company, have supported the study by providing their inhalers to the study team free of charge.

WHO HAS REVIEWED THIS STUDY?

This study was reviewed and approved by the Research Ethics Committee (North East- Newcastle & North Tyneside 2), the Health Research Authority (HRA) and the Medicines and Healthcare products Regulatory Agency (MHRA). The MHRA are responsible for approving all studies involving medicines. The Newcastle Upon Tyne Hospitals NHS Foundation Trust has reviewed all the study documentation and assessed the risks of this study as part of their responsibility, as study Sponsor. This is to ensure that we are not doing anything harmful to you during the study and that your data is collected safely and stored securely. Bronchiectasis patients helped to design this study. They have also looked at the patient information sheet, the consent form and the exacerbation diary.

WHO IS PROVIDING THE STUDY INHALERS?

For peer review only - http://bmjopen.bmj.com/site/about/guidelines.xhtml

The inhalers for all three treatment groups in this study have been provided free of charge by GlaxoSmithKline plc, a pharmaceutical company. They recognise patients with bronchiectasis have no licenced therapies.

WHAT IF RELEVANT NEW INFORMATION BECOMES AVAILABLE?

If, during the course of the study, new information becomes available that is relevant to you, we will tell you about it. We will discuss whether you should or would like to stop the study treatment or withdraw from the study.

WHAT WILL HAPPEN TO THE RESULTS OF THE RESEARCH STUDY?

- The results will be written in medical journals and presented in meetings to other doctors, nurses, researchers and patients.
- A report will be written for the study funder and put on their website.
- All study data that is published will be anonymous. Your identity will always be protected.
- The results will be available at the end of the study through publications, in the wider press and directly to patient groups. You will be able to request a copy of the summary of the results from your study hospital.
- Fully anonymised data may be made available to other researchers to help inform other research studies.

WILL MY TAKING PART IN THIS STUDY BE KEPT CONFIDENTIAL?

Yes. All of the information collected in the study will be entered on computers that are kept secure and password protected.

- You will be given a unique study identification number (called a Participant ID Number) instead of your name being written on study documents. The study team at your hospital will be able to link this number back to you using your date of birth, name, and NHS number (or other nations equivalent). A restricted number of the study team at the Newcastle Clinical Trials Unit may have access to your date of birth, Participant ID number and NHS numbers together. This is for the purpose of requesting the 24 month follow up data, depending on when you join the study.

For peer review only - http://bmjopen.bmj.com/site/about/guidelines.xhtml

- The study team at your hospital will have access to your information during the study to organise planned visits as well as for ongoing safety. They will be in contact with you about receipt of your replacement inhalers and your health
- Your hospital pharmacy/study team will have access to your contact details for posting your replacement study medication
- Your contact details will never be shared with anyone outside of the study with the exception of Royal Mail or an alternative postal/courier service used by your local hospital. You will be asked to consent to the postal service your local hospital uses to have access to your contact details so that they can deliver your replacement inhalers to your home address. The postal services will not know you are in the study just that they need to deliver a package to you
- You will not be named in any results, reports or on websites
- Very occasionally, information might be given during the study that we would have a legal obligation to pass on to others (for instance information which suggested you or others were at risk of harm). In this case, confidentiality would be broken so that we could pass this information to the relevant people. You would be informed of this
- At the end of the study, all study information will be kept in a secure storage area (this is called archiving) for at least 5 years. This makes sure any queries about the running of the study have been answered. All information will be held securely to make sure we protect your confidentiality, after which it will be safely destroyed
- If there are any serious adverse events, we would send details of them to the government medicines agency (MHRA); only your participant ID number would be sent to them. This information may also be sent to researchers outside of the United Kingdom (UK) in the European Economic Area (EEA) who are involved in overseeing the study.
- Some parts of your medical records and the data collected for the study may be looked at by authorised persons from the MHRA, Sponsor (Newcastle Hospitals NHS Foundation Trust) and or the Newcastle Clinical Trials Unit to check that the study is being conducted to the correct standards. All will have a duty of confidentiality to you as a research participant.

For peer review only - http://bmjopen.bmj.com/site/about/guidelines.xhtml

- GlaxoSmithKline plc have asked for de-identified data (age, ethnicity, sex at birth (male/female), site number and disease status and new participant identifier) to be shared with them. This is to allow them to complete their own analyses. This data may be used by anyone in the international company and may leave the European Economic Area (EEA).
- In order to learn about the long-term health of participants in this study your NHS number (or other nations equivalent) may be sent to the Newcastle Clinical Trials Unit, part of Newcastle University. Your information would be transferred and stored securely with access limited to the people that need to know this information.

HOW WILL WE USE INFORMATION ABOUT YOU?

We will need to use information from you, from your medical records and from your GP for this research project.

This information will include your initials, date of birth, NHS number (or other nations equivalent), name and contact details. People will use this information to do the research or to check your records to make sure that the research is being done properly.

People who do not need to know who you are will not be able to see your name or contact details. Your data will have a code number, your unique Participant ID, instead.

We will keep all information about you safe and secure.

Once we have finished the study, we will keep some of the data so we can check the results. We will write our reports in a way that no-one can work out that you took part in the study.

WHAT ARE YOUR CHOICES ABOUT HOW YOUR INFORMATION IS USED?

- You can stop being part of the study at any time, without giving a reason, but we will keep information about you that we already have.

For peer review only - http://bmjopen.bmj.com/site/about/guidelines.xhtml

- If you choose to stop taking part in the study, we would like to continue collecting information about your health from central NHS (or other nations equivalent) records, your hospital and your GP. If you do not want this to happen, tell us and we will stop.
- We need to manage your records in specific ways for the research to be reliable. This means that we won't be able to let you see or change the data we hold about you.

WHERE CAN YOU FIND OUT MORE ABOUT HOW YOUR INFORMATION IS USED?

You can find out more about how we use your information

- at www.hra.nhs.uk/information-about-patients/
- our leaflet available from https://www.newcastle-hospitals.nhs.uk/help/privacy/privacy-notice-for-patients/
- by asking one of the research team
- by sending an email to the Sponsor Data Protection Officer at nuth.dpo@nhs.net
- by ringing the Newcastle upon Tyne Hospital Data Protection Officer on 0191 223 1474

FURTHER INFORMATION AND CONTACT DETAILS

If you have any further questions or would like any further information about the study or the rights of participants, please feel free to contact the people below.

They are also who you or your doctor should contact in the event of an emergency, if your study participation is in any way involved.

[LOCAL CONTACT DETAILS]

Thank you for reading this information sheet.

For peer review only - http://bmjopen.bmj.com/site/about/guidelines.xhtml

Newcastle University

Print on Trust headed paper

Dual Bronchodilators in Bronchiectasis Study (DIBS)

DIBS

INFORMED CONSENT FORM

V2.0 29th March 2021

Participant ID: | | | | | | |

Principal Investigator: ________________________________

		Please INITIAL the boxes if
1.	I confirm that I have read the Participant Information Sheet dated __ __/__ __ __/__ __ __ __ version ___ for the above study and that I have had the opportunity to consider the information, ask questions and have had these answered satisfactorily.	
2.	I understand that my participation is voluntary and that I am free to withdraw at any time without giving any reason, without my medical care or legal rights being affected.	
3.	I consent to receiving the study medication outlined in the Participant Information Sheet.	
4.	I understand that my personal data (name, address, telephone number) will be used for the purposes of posting study inhalers to my home address and for contacting me. I give permission for this information to be stored by responsible people at my local NHS Trust and for them to be transferred to Royal Mail, or any alternative postal or courier service used by my local hospital, so that my replacement inhalers can be posted to my home address.	
5.	I understand that information about me that is relevant to this study will leave the Trust and be sent securely to Newcastle University. This includes my date of birth, gender and ethnicity which will be stored on the study database. My NHS number will also be collected and stored separately in order to collect data about my long-term health, and could be accessed for up to three years after my enrollment into this study. I understand that my data will be stored securely and managed confidentially as part of this study.	
6.	I understand that any personal information collected about me for the study will be kept confidential and not be made public. I understand that data from the study will be	

For peer review only - http://bmjopen.bmj.com/site/about/guidelines.xhtml

	published in medical journals, at research meetings and shared with other researchers, including researchers potentially outside the United Kingdom (UK) in the European Economic Area (EEA). I understand that data from the study will be de-identified and that I will not be directly identified in the published results.	
7.	I understand that de-identified data from the study will be shared with GlaxoSmithKline plc (GSK), including the following personal information:<br>• Age<br>• Sex at birth (female/male)<br>• Information about my bronchiectasis disease<br>• Ethnicity<br>• Study identification number<br>I understand that I will not be directly identified in any data shared with GSK.	
8.	I consent to study data being transferred to GSK plc. I understand that GSK is an international company and that data may be transferred outside of the United Kingdom (UK).	
9.	I understand that the information held and maintained by:<br>• Bronchiectasis Databases (including BronchUK [The United Kingdom Bronchiectasis Registry] and EMBARC [European Multicentre Bronchiectasis Audit and Research Collaboration], if you have previously consented to take part in these projects)<br>• NHS Digital (including Hospital Episodes Statistics [HES]-not applicable for Scottish sites)<br>• Central UK NHS bodies (including the Office for National Statistics [ONS]-not applicable for Scottish sites)<br>may be used to provide information about my health status to the research team. I consent to the research team requesting information about my health from routine sources, including Bronchiectasis Databases, NHS Digital and ONS (if applicable) as needed, including up to one year after my participation in the study finishes, and to reviewing my medical records to compare this information.	
10.	I understand that the information collected about me will be used to support other research in the future, and may be shared anonymously with other researcher projects and researchers.	

For peer review only - http://bmjopen.bmj.com/site/about/guidelines.xhtml

11.	I understand that parts of my medical records and data collected during the study may be looked at by responsible people. I give my permission for these people to have access to my medical records.<br>This includes people from Newcastle University, study Sponsor, regulatory authorities and local NHS Trust where it is relevant to my taking part in research.	
12.	I understand that the information provided in this study is being managed by the Newcastle Clinical Trials Unit, which is part of Newcastle University.	
13.	I agree to my General Practitioner being involved in the study, including any necessary exchange of information about me between my GP and the research team	
14.	I agree to the information provided and this signed consent form being stored for 5 years after the end of the study.	
15.	I agree to take part in the above study.	

For participants of childbearing potential:		
16.	I understand that I will have to use an effective form of contraception as outlined in the Participant Information Sheet, if sexually active.	
17.	Females only: I understand that if I am of childbearing potential, I will need to have a urine pregnancy test to ensure that I am not pregnant. I understand that this is for safety reasons.	

Name of Participant	Date	Signature
Name of Person taking consent	Date	Signature

When completed: File 1 copy in the investigator site file (original); Provide 1 copy for the study participant; File 1 copy in the participant's medical notes.

For peer review only - http://bmjopen.bmj.com/site/about/guidelines.xhtml

For peer review only

For peer review only - http://bmjopen.bmj.com/site/about/guidelines.xhtml

Reporting checklist for protocol of a clinical trial.

Based on the SPIRIT guidelines.

Instructions to authors

Complete this checklist by entering the page numbers from your manuscript where readers will find each of the items listed below.

Your article may not currently address all the items on the checklist. Please modify your text to include the missing information. If you are certain that an item does not apply, please write "n/a" and provide a short explanation.

Upload your completed checklist as an extra file when you submit to a journal.

In your methods section, say that you used the SPIRITreporting guidelines, and cite them as:

Chan A-W, Tetzlaff JM, Gøtzsche PC, Altman DG, Mann H, Berlin J, Dickersin K, Hróbjartsson A, Schulz KF, Parulekar WR, Krleža-Jerić K, Laupacis A, Moher D. SPIRIT 2013 Explanation and Elaboration: Guidance for protocols of clinical trials. BMJ. 2013;346:e7586

		Reporting Item	Page Number
**Administrative information**			
Title	#1	Descriptive title identifying the study design, population, interventions, and, if applicable, trial acronym	1
Trial registration	#2a	Trial identifier and registry name. If not yet registered, name of intended registry	2
Trial registration: data set	#2b	All items from the World Health Organization Trial Registration Data Set	1-22
Protocol version	#3	Date and version identifier	19
Funding	#4	Sources and types of financial, material, and other support	21
Roles and responsibilities: contributorship	#5a	Names, affiliations, and roles of protocol contributors	21

For peer review only

Roles and responsibilities: sponsor contact information	#5b	Name and contact information for the trial sponsor	4
Roles and responsibilities: sponsor and funder	#5c	Role of study sponsor and funders, if any, in study design; collection, management, analysis, and interpretation of data; writing of the report; and the decision to submit the report for publication, including whether they will have ultimate authority over any of these activities	4
Roles and responsibilities: committees	#5d	Composition, roles, and responsibilities of the coordinating centre, steering committee, endpoint adjudication committee, data management team, and other individuals or groups overseeing the trial, if applicable (see Item 21a for data monitoring committee)	4 - 5
**Introduction**			
Background and rationale	#6a	Description of research question and justification for undertaking the trial, including summary of relevant studies (published and unpublished) examining benefits and harms for each intervention	3 - 4
Background and rationale: choice of comparators	#6b	Explanation for choice of comparators	3 - 5
Objectives	#7	Specific objectives or hypotheses	6 - 9
Trial design	#8	Description of trial design including type of trial (eg, parallel group, crossover, factorial, single group), allocation ratio, and framework (eg, superiority, equivalence, non-inferiority, exploratory)	4
**Methods: Participants, interventions, and outcomes**			

For peer review only

Study setting	#9	Description of study settings (eg, community clinic, academic hospital) and list of countries where data will be collected. Reference to where list of study sites can be obtained	4
Eligibility criteria	#10	Inclusion and exclusion criteria for participants. If applicable, eligibility criteria for study centres and individuals who will perform the interventions (eg, surgeons, psychotherapists)	5 - 6
Interventions: description	#11a	Interventions for each group with sufficient detail to allow replication, including how and when they will be administered	10 – 11
Interventions: modifications	#11b	Criteria for discontinuing or modifying allocated interventions for a given trial participant (eg, drug dose change in response to harms, participant request, or improving / worsening disease)	10, 13
Interventions: adherance	#11c	Strategies to improve adherence to intervention protocols, and any procedures for monitoring adherence (eg, drug tablet return; laboratory tests)	11
Interventions: concomitant care	#11d	Relevant concomitant care and interventions that are permitted or prohibited during the trial	N/A – none specified by trial
Outcomes	#12	Primary, secondary, and other outcomes, including the specific measurement variable (eg, systolic blood pressure), analysis metric (eg, change from baseline, final value, time to event), method of aggregation (eg, median, proportion), and time point for each outcome. Explanation of the clinical relevance of chosen efficacy and harm outcomes is strongly recommended	6 - 9
Participant timeline	#13	Time schedule of enrolment, interventions (including any run-ins and washouts), assessments, and visits for participants. A schematic diagram is highly recommended (see Figure)	11 - 12

For peer review only

Sample size	#14	Estimated number of participants needed to achieve study objectives and how it was determined, including clinical and statistical assumptions supporting any sample size calculations	17 - 18
Recruitment	#15	Strategies for achieving adequate participant enrolment to reach target sample size	9, 18
**Methods: Assignment of interventions (for controlled trials)**			
Allocation: sequence generation	#16a	Method of generating the allocation sequence (eg, computer-generated random numbers), and list of any factors for stratification. To reduce predictability of a random sequence, details of any planned restriction (eg, blocking) should be provided in a separate document that is unavailable to those who enrol participants or assign interventions	10
Allocation concealment mechanism	#16b	Mechanism of implementing the allocation sequence (eg, central telephone; sequentially numbered, opaque, sealed envelopes), describing any steps to conceal the sequence until interventions are assigned	10
Allocation: implementation	#16c	Who will generate the allocation sequence, who will enrol participants, and who will assign participants to interventions	10
Blinding (masking)	#17a	Who will be blinded after assignment to interventions (eg, trial participants, care providers, outcome assessors, data analysts), and how	10 - 11
Blinding (masking): emergency unblinding	#17b	If blinded, circumstances under which unblinding is permissible, and procedure for revealing a participant's allocated intervention during the trial	13
**Methods: Data collection,**			

For peer review only

**management, and analysis**			
Data collection plan	#18a	Plans for assessment and collection of outcome, baseline, and other trial data, including any related processes to promote data quality (eg, duplicate measurements, training of assessors) and a description of study instruments (eg, questionnaires, laboratory tests) along with their reliability and validity, if known. Reference to where data collection forms can be found, if not in the protocol	13 - 14
Data collection plan: retention	#18b	Plans to promote participant retention and complete follow-up, including list of any outcome data to be collected for participants who discontinue or deviate from intervention protocols	13
Data management	#19	Plans for data entry, coding, security, and storage, including any related processes to promote data quality (eg, double data entry; range checks for data values). Reference to where details of data management procedures can be found, if not in the protocol	13 – 14
Statistics: outcomes	#20a	Statistical methods for analysing primary and secondary outcomes. Reference to where other details of the statistical analysis plan can be found, if not in the protocol	14
Statistics: additional analyses	#20b	Methods for any additional analyses (eg, subgroup and adjusted analyses)	15 - 16
Statistics: analysis population and missing data	#20c	Definition of analysis population relating to protocol non-adherence (eg, as randomised analysis), and any statistical methods to handle missing data (eg, multiple imputation)	14 - 15
**Methods: Monitoring**			
Data monitoring: formal committee	#21a	Composition of data monitoring committee (DMC); summary of its role and reporting	5

For peer review only

		structure; statement of whether it is independent from the sponsor and competing interests; and reference to where further details about its charter can be found, if not in the protocol. Alternatively, an explanation of why a DMC is not needed	
Data monitoring: interim analysis	#21b	Description of any interim analyses and stopping guidelines, including who will have access to these interim results and make the final decision to terminate the trial	No interim analysis
Harms	#22	Plans for collecting, assessing, reporting, and managing solicited and spontaneously reported adverse events and other unintended effects of trial interventions or trial conduct	14
Auditing	#23	Frequency and procedures for auditing trial conduct, if any, and whether the process will be independent from investigators and the sponsor	13
**Ethics and dissemination**			
Research ethics approval	#24	Plans for seeking research ethics committee / institutional review board (REC / IRB) approval	19
Protocol amendments	#25	Plans for communicating important protocol modifications (eg, changes to eligibility criteria, outcomes, analyses) to relevant parties (eg, investigators, REC / IRBs, trial participants, trial registries, journals, regulators)	18 - 19
Consent or assent	#26a	Who will obtain informed consent or assent from potential trial participants or authorised surrogates, and how (see Item 32)	9
Consent or assent: ancillary studies	#26b	Additional consent provisions for collection and use of participant data and biological specimens in ancillary studies, if applicable	N/A – no ancillary studies
Confidentiality	#27	How personal information about potential and enrolled participants will be collected, shared, and	13 - 14

For peer review only

		maintained in order to protect confidentiality before, during, and after the trial	
Declaration of interests	#28	Financial and other competing interests for principal investigators for the overall trial and each study site	22
Data access	#29	Statement of who will have access to the final trial dataset, and disclosure of contractual agreements that limit such access for investigators	13 - 14
Ancillary and post trial care	#30	Provisions, if any, for ancillary and post-trial care, and for compensation to those who suffer harm from trial participation	
Dissemination policy: trial results	#31a	Plans for investigators and sponsor to communicate trial results to participants, healthcare professionals, the public, and other relevant groups (eg, via publication, reporting in results databases, or other data sharing arrangements), including any publication restrictions	19
Dissemination policy: authorship	#31b	Authorship eligibility guidelines and any intended use of professional writers	N/A
Dissemination policy: reproducible research	#31c	Plans, if any, for granting public access to the full protocol, participant-level dataset, and statistical code	N/A
**Appendices**			
Informed consent materials	#32	Model consent form and other related documentation given to participants and authorised surrogates	Supplementary
Biological specimens	#33	Plans for collection, laboratory evaluation, and storage of biological specimens for genetic or molecular analysis in the current trial and for future use in ancillary studies, if applicable	N/A – no biological specimens

None The SPIRIT Explanation and Elaboration paper is distributed under the terms of the Creative Commons Attribution License CC-BY-NC. This checklist can be completed online using

For peer review only

https://www.goodreports.org/, a tool made by the EQUATOR Network in collaboration with Penelope.ai

For peer review only

BMJ Open

Dual Bronchodilators in Bronchiectasis Study (DIBS): protocol for a pragmatic, multicentre, placebo controlled, 3 arm, double-blinded, randomised controlled trial studying bronchodilators in preventing exacerbations of bronchiectasis

Journal:	*BMJ Open*
Manuscript ID	bmjopen-2023-071906.R1
Article Type:	Protocol
Date Submitted by the Author:	08-Jun-2023
Complete List of Authors:	Morton, Miranda; Newcastle University, Newcastle Clinical Trials Unit<br>Wilson, Nina; Newcastle University Faculty of Medical Sciences, Population Health Sciences Institute<br>Homer, Tara; Newcastle University, Health Economics Group<br>Simms, Laura; Newcastle University,<br>Steel, Alison; Newcastle University, Newcastle Clinical Trials Unit<br>Maier, Rebecca; Newcastle University, Newcastle Clinical Trials Unit<br>Wason, James; Newcastle University, Population Health Sciences Institute<br>Ternent, Laura; Newcastle University, Health Economics Group<br>Abouhajar, Alaa; Newcastle University, Newcastle Clinical Trials Unit<br>Allen, Maria; The Newcastle upon Tyne Hospitals NHS Foundation Trust<br>Joyce, Richard; Newcastle University, Newcastle Clinical Trials Unit<br>Hildreth, Victoria; Newcastle University, Newcastle Clinical Trials Unit<br>Lakey, Rachel; Newcastle University, Newcastle Clinical Trials Unit<br>Cherlin, Svetlana; Newcastle University<br>Walker, Adam; The Newcastle upon Tyne Hospitals NHS Foundation Trust<br>Devereux, Graham ; Liverpool School of Tropical Medicine, Department of Clinical Sciences<br>Chalmers, J; University of Dundee,<br>Hill, A; The University of Edinburgh<br>Haworth, Charles; Royal Papworth Hospital NHS Foundation Trust<br>Hurst, John; UCL Medical School,, Academic Unit of Respiratory Medicine<br>De Soyza, Anthony; Newcastle University
**Primary Subject Heading**:	Respiratory medicine
Secondary Subject Heading:	Research methods
Keywords:	RESPIRATORY MEDICINE (see Thoracic Medicine), Adult thoracic medicine < THORACIC MEDICINE, Clinical trials < THERAPEUTICS

Dual Bronchodilators in Bronchiectasis Study (DIBS): protocol for a pragmatic, multicentre, placebo controlled, 3 arm, double-blinded, randomised controlled trial studying bronchodilators in preventing exacerbations of bronchiectasis

Authors: Miranda Morton[1], Nina Wilson[2], Tara Homer[3], Laura Simms[1], Alison Steel[1], Rebecca Maier[1], James Wason[2], Laura Ternent[3], Alaa Abouhajar[1], Maria Allen[4], Richard Joyce[1], Victoria Hildreth[1], Rachel Lakey[1], Svetlana Cherlin[2], Adam Walker[5], Graham Devereux[6], James Chalmers[7], Adam T Hill[8], Charles Haworth[9], John Hurst[10], Anthony De Soyza[11]

[1] Newcastle Clinical Trials Unit, Newcastle University, Newcastle upon Tyne, UK. [2] Biostatistics Research Group, Newcastle University, Newcastle upon Tyne, UK. [3] Health Economics Group, Newcastle University, Newcastle upon Tyne, UK. [4] R&D Pharmacy, The Newcastle upon Tyne Hospitals NHS Foundation Trust, Newcastle upon Tyne, UK. [5] Newcastle Specials (Pharmacy Production Unit), The Newcastle upon Tyne Hospitals NHS Foundation Trust, Newcastle upon Tyne, UK. [6] Liverpool School of Tropical Medicine, Liverpool, UK. [7] School of Medicine, University of Dundee, Dundee, UK. [8] Department of Respiratory Medicine, Royal Infirmary of Edinburgh, Edinburgh, UK. [9] Cambridge Centre for Lung Infection, Royal Papworth Hospital NHS Foundation Trust, Cambridge, UK. [10] Centre for Inflammation and Tissue Repair, UCL, London, UK. [11] Faculty of Medical Sciences, Newcastle University, Newcastle upon Tyne, UK.

Corresponding Author: Anthony De Soyza, Professor of Pulmonary Medicine. Postal address: Population Health Sciences Institute, Faculty of Medical Sciences, Newcastle University, Room L2060, Framlington Place, Newcastle upon Tyne, NE2 4HH. Email address: Anthony.de-soyza@newcastle.ac.uk

Key words: Bronchiectasis, Bronchodilators, Inhaled steroid

Word count (excluding title page, abstract, references, figures and tables): 5091

For peer review only - http://bmjopen.bmj.com/site/about/guidelines.xhtml

ABSTRACT

Introduction

Bronchiectasis is a long-term lung condition, with dilated bronchi, chronic inflammation, chronic infection and acute exacerbations. Recurrent exacerbations are associated with poorer clinical outcomes such as increased severity of lung disease, further exacerbations, hospitalisations, reduced quality of life and increased risk of death. Despite an increasing prevalence of bronchiectasis, there is a critical lack of high-quality studies into the disease and no treatments specifically approved for its treatment. This trial aims to establish whether inhaled dual bronchodilators (long acting beta agonist [LABA] and long acting muscarinic antagonist [LAMA]) taken as either a stand-alone therapy or in combination with inhaled corticosteroid (ICS) reduce the number of exacerbations of bronchiectasis requiring treatment with antibiotics during a 12 month treatment period.

Methods

This is a multicentre, pragmatic, double blind, randomised controlled trial, incorporating an internal pilot and embedded economic evaluation. 600 adult patients (≥ 18 years) with CT confirmed bronchiectasis will be recruited and randomised to either inhaled dual therapy (LABA + LAMA), triple therapy (LABA + LAMA + ICS) or matched placebo, in a 2:2:1 ratio (respectively). The primary outcome is the number of protocol defined exacerbations requiring treatment with antibiotics during the 12 month treatment period.

Ethics and Dissemination

Favourable ethical opinion was received from the North East – Newcastle & North Tyneside 2 Research Ethics Committee (reference: 21/NE/0020). Results will be disseminated in peer reviewed publications, at national and international conferences, in the NIHR HTA journal and to participants and the public (using lay language).

Registration

ISRCTN15988757

STRENGTHS AND LIMITATIONS OF THIS TRIAL

- Large randomised controlled trial to address unmet clinical need and critical lack of research into bronchiectasis treatments
- Pragmatic trial design conducted over multiple sites in the UK
- Challenge of delivering a large multicentre trial in the respiratory disease area in the peri COVID-19 era

For peer review only - http://bmjopen.bmj.com/site/about/guidelines.xhtml

- Challenge of a static trial design with changing biology of bronchiectasis following shielding behaviours during the COVID-19 pandemic

INTRODUCTION

Bronchiectasis is a chronic lung condition, characterised by dilated bronchi, leading to symptoms of breathlessness and chronic productive cough, with intermittent infective exacerbations. Bronchiectasis has various potential aetiologies including immune-deficiency syndromes, allergic bronchopulmonary aspergillosis, chronic obstructive pulmonary disease, ciliary dysfunction and post-infectious, yet studies have found that between a quarter and half of cases are idiopathic [1, 2]. Patients often have recurrent, costly hospital admissions, a poorer quality of life [3, 4] and clinically significant fatigue [5, 6].

Current estimates suggest a prevalence of 100,000 adult patients with the condition in the UK. Importantly, studies demonstrate that up to 50% of patients with chronic obstructive pulmonary disease (COPD) have co-existent bronchiectasis [7]. With approximately 1,000,000 patients with COPD in the UK [8], there is potential for a significant increase of case-finding of COPD-associated bronchiectasis over the coming years.

Bronchiectasis mortality rates are approximately 50% higher than that of uncomplicated COPD (calculated at 3% per annum) and have been reported to be increasing [9]. Prognosis varies, with a prior study of 91 patients [10] finding that the primary cause of death was usually respiratory, with survival rates of 91% at 4 years and 68.3% at 12.3 years. The same study found factors such as chronic infection with *Pseudomonas aeruginosa* increase mortality.

Multicentre data suggests the Bronchiectasis severity index (BSI) a multi component clinical scoring system is useful in predicting both mortality and morbidity (hospitalisation). This score was validated in 1300 patients across Europe [11].

Infective exacerbations lead to significant morbidity. Within the UK national audit data patient group average exacerbation rate is approximately 3 per year (nearly twice the rate of COPD) with an attendant risk of hospitalisation. This is consistent with published American data on the increasing burden of bronchiectasis [12] and in the UK [13]. Previously published UK data also emphasises the burden of bronchiectasis, uncertainties in aetiology and lack of evidence for the treatments that are often used [14]. Hence, improved interventions in bronchiectasis are urgently required.

There is no cure for bronchiectasis. Current modalities of treatment include oral, inhaled or intravenous antibiotics given regularly with additional courses administered for exacerbations. Mucolytics and regular physiotherapy are used to aid sputum clearance and there are additional

For peer review only - http://bmjopen.bmj.com/site/about/guidelines.xhtml

guidelines for investigation, diagnosis and management of bronchiectasis produced by the British Thoracic Society (BTS) [15].

National audits in the UK suggest up to 80% of patients with bronchiectasis are on inhaled therapy despite limited evidence [15]. Prior studies of inhaled cortico-steroids (ICS) in bronchiectasis have shown no clear benefit [15] and are therefore not recommended in prevailing bronchiectasis guidelines. ICS, however, have been shown to have benefits in asthma [16] and in COPD [17]. More recent data emerging from COPD suggests that ICS reduce COPD exacerbation rates but increases pneumonia rates, a side effect that may be very pertinent in bronchiectasis. International guidelines for COPD now suggest a selective application of ICS and that not all patients with COPD benefit from ICS therapy [17]

The role of triple therapy (ICS/LABA/LAMA) and dual bronchodilation (LABA/LAMA) therapy is unclear in bronchiectasis with no robust randomised placebo-controlled data available. Given the likely higher bacterial and inflammatory load in bronchiectasis airways these therapies need be specifically studied within this population

METHODS AND ANALYSIS

Trial methods and analysis are reported as per SPIRIT reporting guidelines [18].

Trial Design

DIBS is a pragmatic, multi-centre, placebo-controlled, three-arm, double blinded, randomised controlled trial, incorporating an internal pilot. The trial aim is to recruit a total of 600 adult patients with bronchiectasis from up to 25 secondary care NHS sites. The trial is Sponsored by The Newcastle upon Tyne Hospitals NHS Foundation Trust (tnu-tr.sponsormanagement@nhs.net).

The internal pilot involves approximately 15 secondary care NHS sites recruiting for a 12-month recruitment period (following activation of the first site to recruitment). Site activation of the NHS sites will be as soon as possible with a staggered approach. The recruitment aim is to recruit 98-125 participants equating to an average recruitment between 1.25 and 1.6 participants recruited per site per month. At the end of the internal pilot a review of will be made by the Trial Management Group (TMG), the Trial Steering Committee (TSC), the Independent Data Monitoring and Ethics Committee (IDMEC) and a consultation with the funder against the following progression criteria to proceed from the internal pilot to the main trial recruitment:

- Average recruitment ≥ 1.6 participants/month/site activated – continue to main trial and open additional sites (up to 25 sites total)

For peer review only - http://bmjopen.bmj.com/site/about/guidelines.xhtml

- Average recruitment ≥ 1.25 participants/month/site activated – continue to main trial and open additional sites (up to 25 sites total) plus provide an improvement plan after identifying barriers to recruitment through discussion with sites, TMG, TSC and IDMEC as required
- Average recruitment < 1.25 participants/month/site activated – seek further guidance from funder

The TSC and IDMEC are composed of independent experts in the field (clinicians, statisticians) and patient a public representatives. Each committees' roles are defined in their charters.

The main trial phase will follow on from the pilot without a break in recruitment. The main phase of the trial will involve an additional 10 secondary care NHS sites bringing the total number of sites recruiting to the trial to 25 sites. The recruitment target for each site remains 1 – 2 patients per month throughout the main phase of recruitment which will last for 14 months. The last patient last visit will be 12 months after the last participant has been recruited.

Patient eligibility – Inclusion and Exclusion Criteria

Patients are eligible for the trial is all of the following inclusion criteria apply:

1) Adult patients with CT (Computed Tomography) scan confirmed bronchiectasis and bronchiectasis is the predominant primary respiratory disease in the view of the investigator (CT images / CT reports must be available to complete radiological scoring for Bronchiectasis Severity Index)
2) History of 2 or more exacerbations in any 12-month period in the preceding 2 years requiring antibiotics and/or steroids
3) Evidence of airflow limitation with an FEV1/FVC ratio less than 0.7 and/or daily mucus expectoration
4) Have either (i) less than 20 pack year history of smoking OR (ii) greater than 20 pack year history of smoking with FEV1 >79% predicted (to exclude COPD)
5) For patients taking ICS, LAMA or LABA treatment prior to recruitment, willing to have these treatments changed or stopped
6) Stable bronchiectasis with no exacerbations for 4 weeks prior to baseline
7) Stable dose of oral steroid for 4 weeks prior to baseline (only applicable for patients taking oral steroid as part of standard care)

Patients are excluded from the trial if any of the following exclusion criteria apply:

1) Cystic fibrosis related bronchiectasis

For peer review only - http://bmjopen.bmj.com/site/about/guidelines.xhtml

2) Where bronchiectasis is not the main disease or there are contraindications to ICS withdrawal
3) Predominant COPD or asthma *
   *Patients who have a historical diagnosis of asthma and/or COPD but where the investigator has sufficient evidence to refute these diagnoses can still be included
4) Indication to remain on ICS (e.g. asthma, COPD, allergic bronchopulmonary aspergillosis, inflammatory bowel disease) or known intolerance to any of the trial drugs or their ingredients
5) Patients with galactose intolerance, total lactase deficiency or glucose-galactose malabsorption
6) Inability to perform spirometry or quality of life questionnaires
7) Patients who are:
   a) pregnant
   b) breast feeding
   c) of childbearing potential with a positive urine pregnancy test prior to starting trial IMP
   d) male or female of childbearing potential unwilling to use contraception throughout the trial (postmenopausal women must be amenorrhoeic for at least 12 months to be considered of non-childbearing potential).
8) Anyone with cognitive impairment who may not be able to consent
9) Those who do not speak English or cannot comply with trial procedures
10) Any potential participant who the investigator believes will not be able to complete the trial visits and procedures
11) A history of allergy or hypersensitivity to any corticosteroid, anticholinergic/muscarinic receptor antagonist, β2-agonist, lactose/milk protein or magnesium stearate or a medical condition such as narrow-angle glaucoma, prostatic hypertrophy or bladder neck obstruction that, in the opinion of the investigator contraindicates trial participation
12) Use of acute antibiotics or systemic steroids within 4 weeks of baseline (except for antibiotics and/or stable doses of prednisone ≤ 5mg used to treat non-respiratory conditions)
13) Malignancy diagnosed within 5 years of the first trial medication administration where the investigator feels the trial may be affected by recurrence or progression of the malignancy (e.g. patients with stable breast cancer, current prostate cancer or “expected curative” cancer surgery) may not be excluded at the investigators discretion
14) Administration of an investigational agent within 30 days of first dose of trial medication

Objectives and Outcomes

For peer review only - http://bmjopen.bmj.com/site/about/guidelines.xhtml

The primary objective for the trial is to compare the effects of inhaled dual bronchodilators either as a stand-alone dual therapy (LABA + LAMA) or in combination with inhaled corticosteroid as a triple therapy (LABA + LAMA + ICS) to placebo on the number of protocol defined bronchiectasis exacerbations experienced per participant over the 12-month trial treatment period. The protocol definition of a bronchiectasis exacerbation is a continued worsening of one or more of symptoms including increased cough, sputum discolouration, excess sputum production, breathlessness and/or fatigue, that require treatment with antibiotic(s).

Additionally, there is a primary economic objective to compare the cost-effectiveness, measured in terms of incremental cost per quality-adjusted life-year (QALY) gained over the 12-month trial treatment period.

Secondary objectives include comparisons between the effects of dual therapy (LABA + LAMA), triple therapy (LABA + LAMA + ICS) and placebo on the number of bronchiectasis related hospital admissions, the number of emergency hospital admissions, the time to the first exacerbation of bronchiectasis following randomisation, the number of adverse events and cessation of treatment, quality of life, breathlessness and lung function, respiratory and cardiac mortality, economic costs and the incremental cost per QALY gained over the patient's life course. Objectives and Outcome measures are described fully in

Table 1.

Table 1. Objectives and outcome measures

Objectives		Outcome Measures
**Primary**	Compare effects of inhaled dual bronchodilators either as a stand-alone therapy (LABA/LAMA) or in combination with ICS (ICS/LABA/LAMA; triple therapy) therapy to placebo on the number of protocol defined bronchiectasis exacerbations (per participant)	• Number of bronchiectasis exacerbations requiring treatment with antibiotics during 12-month treatment period as measured using participant reports and completed weekly exacerbation diary.
**Primary Economic**	Compare cost-effectiveness measured in terms of incremental cost-per-QALY gained over the 12 month treatment period.	• Incremental cost-per QALY at 12 months. Costs based on the cost of the interventions, use of health services via a Health Care Utilisation Questionnaire administered at baseline, 1, 6 and 12 months post randomisation and adverse events.<br>• Transport and time for participants to utilise health care appointments will be assessed via the Time and Travel Questionnaire administered at 12 months post randomisation.

For peer review only - http://bmjopen.bmj.com/site/about/guidelines.xhtml

**Secondary**	To compare the effects of dual bronchodilators (LABA/LAMA) and triple (ICS/LABA/LAMA) therapy and placebo on:	
	• Hospital admissions with a primary diagnosis of exacerbation of bronchiectasis	• Number of hospital admissions for bronchiectasis exacerbations during 12-month treatment period as measured using participant reports and completed weekly exacerbation diary and verified where possible by hospital discharge summary/HES data.<br>• Hospitalisation due to bronchiectasis exacerbation data collected up to 24 months after visit 1: screening/baseline, will be used to extend modelling beyond 12 months as a sensitivity analysis.
	• Time to first exacerbation of bronchiectasis	• Time to first exacerbation of bronchiectasis as measured using participant reports /completed weekly exacerbation diary.
	• Number of emergency hospital admissions	• Number of emergency hospital admissions (all cause) as ascertained at 1, 6 and 12 months visits and where needed from primary care records.
	• Adverse events/drug reactions and cessation of treatment	• Number of adverse events/drug reactions and cessation of treatment as reported by participant to research team and at 1-, 6- and 12-months visits.
	• Disease related health status using the St Georges Respiratory questionnaire (SGRQ) and Quality of Life Bronchiectasis (QOL-B) questionnaire	• SGRQ and QOL-B at baseline, 1-, 6- and 12-months visits.
	• Health related quality of life using the EQ-5D-5L questionnaire	• EQ-5D-5L at baseline, 1-, 6- and 12-months visits.
	• Breathlessness using Baseline and Transition Dyspnoea Indices (BDI and TDI)	• BDI at baseline and TDI at 1-, 6- and 12-months visits.
	• Lung function using spirometry	Post bronchodilator lung function (LABA within 8 hours, short acting beta2 agonist within 2 hours) as measured by

For peer review only - http://bmjopen.bmj.com/site/about/guidelines.xhtml

		spirometry performed to ATS/ERS standards at baseline, 1, 6 and 12 month visits:<br>• forced expiratory volume in 1 second (FEV1)<br>• forced vital capacity (FVC)
	• All-cause, respiratory and cardiac mortality	• As ascertained from medical records or Office for National Statistics (ONS) records of trial participants (collected up to 24 months after visit 1: screening/baseline).
	• Incremental cost per exacerbation avoided	• Costs based on cost of the interventions (micro-costed) and use of health services collected via a Health Care Utilisation Questionnaire administered at baseline, 1-, 6- and 12-months post randomisation and adverse events collected via case report forms.
	• Costs to the NHS and patients and lifetime cost-effectiveness based on extrapolation modelling	• Data to populate the model will come from the trial and will be supplemented by Hospital Episode Statistics (HES) and ONS data (collected up to 24 months after visit 1: screening/baseline) and by relevant literature and expert opinion and extrapolated over a patient lifetime.
	• Rates of radiologically confirmed pneumonia, compared to participant's normal baseline	• Number of pneumonia events and total number of participants suffering pneumonia. This will be measured by asking the participants during follow up visits.
**Exploratory**	• To investigate the relationship between key outcomes (exacerbations and quality of life as measured by SGRQ and QOL-B) with baseline eosinophil (single level recorded at baseline), median eosinophil level (median of last 3 available recording when not on oral steroids) and baseline BSI	• Eosinophil measurement at baseline and the median of the last 3 measurements available<br>• SGRQ and QOL-B as completed by participants at 1-, 6- and 12-month follow-up visits<br>• BSI measured at baseline<br>• The number of protocol defined bronchiectasis exacerbations requiring treatment with antibiotics during 12-month treatment period as

For peer review only - http://bmjopen.bmj.com/site/about/guidelines.xhtml

		measured using participant reports/participant completed weekly exacerbation diary.

Identification, consent and screening of patients

Potential participants aged 18 years or older with CT scan confirmed bronchiectasis, where bronchiectasis is the predominant primary respiratory disease, will be identified and recruited through secondary care NHS sites. Potential participants will be identified by clinicians and members of the research team from patients attending outpatient clinics, spirometry, oxygen services, in-patient admissions and by examination of databases such as local, BronchUK [19] and EMBARC [20] registries. Primary care practices may also be set up as Participant Identification Centres (PICs) to identify potential participants and signpost them to an appropriate secondary care recruitment site.

Potential participants will be approached in person or by post with a trial specific Patient Information Sheet (PIS) (see supplementary file). Each potential participant will have time to read and consider the PIS before having an opportunity to discuss the trial further with a member of the research team. Written, informed consent using a trial specific Consent Form (see supplementary file) will be obtained by a medically qualified member of the research team prior to any trial specific screening activity.

Screening of potential participants will involve:

- Collection of demographic data, medical history, medication history and smoking history
- Lung function as measured by spirometry
- Modified Reiff scoring [21] of the latest CT scan performed as part of standard care
- Quality of Life measurements including Quality of Life Bronchiectasis (QoL-B) questionnaire [22], St George's Respiratory Questionnaire (SGRQ) [4] and the 5-level EuroQol5D index (EQ-5D-5L) questionnaire [23]
- BSI Score [11]
- Assessments of breathlessness including the MRC Dyspnoea Score [24] and BDI and TDI respectively [25]
- Health Economics questionnaires
- Baseline bloods (full blood count including differentials)

All participants of childbearing must be willing to use an acceptable form of contraception (as listed in the PIS) throughout the trial from the date of consent until 7 days after their last dose of trial medication. Additionally, women of childbearing potential will be required to have a negative urine pregnancy test at baseline/screening to be eligible for the trial.

For peer review only - http://bmjopen.bmj.com/site/about/guidelines.xhtml

Randomisation

Patients who are confirmed as eligible will be randomised to one of three treatment arms dual therapy (LABA + LAMA), triple therapy (LABA + LAMA + ICS) or matched placebo, in a 2:2:1 ratio (respectively). Randomisation is carried out by appropriately delegated members of the research team using random permuted blocks of variable length within strata via the Sealed Envelope system; a central, secure, 24-hour web-based randomisation system with concealed allocation.

Randomisation is stratified by two variables: BSI score (BSI score of 0-8 or 9+) and by baseline ICS drug therapy (ICS user or non-ICS user at baseline).

Intervention

Treatment allocation is double-blind, each inhaler is identical in appearance to maintain the blind. Participants will have a dose of one inhalation of their trial inhaler per day, it is advised that the dose is taken at the same time of day wherever possible, for the 12-month (365 days) trial period. Dose modifications are not permitted.

The contents of each inhaler are described in ***Table 2***.

Table 2. Inhaler contents

INHALER	CONTENTS OF EACH DOSE DELIVERED	NAME OF EQUIVALENT COMMERCIALLY AVAILABLE PRODUCT
Dual therapy (LAMA/LABA)	55 micrograms umeclidinium<br>22 micrograms vilanterol	Anoro Ellipta dry powder inhaler
Triple therapy (ICS/LAMA/LABA)	92 micrograms fluticasone furoate<br>55 micrograms umeclidinium<br>22 micrograms vilanterol	Trelegy Ellipta dry powder inhaler
Placebo	placebo	Matched placebo dry powder inhaler

Each inhaler contains 30 doses of medication. Due to the shelf life of the labelled inhalers, they will be shipped to participants approximately monthly throughout their participation in the trial. Confirmation of receipt of trial medication will be carried out by a member of the research staff telephoning the patient following each trial medication shipment. Trial inhalers are returned by participants at the end of their involvement in the trial and compliance monitored.

Follow-up visits and participant assessments

For peer review only - http://bmjopen.bmj.com/site/about/guidelines.xhtml

For peer review only

Follow-up visits will take place at the local trial site at 1-, 6- and 12-months with assessments performed as detailed in Table 3.

Participants will be required to complete an Exacerbation Diary weekly throughout their 12-month participation in the trial. In this diary participants will record whether or not they have experienced any exacerbations during the week and any steps or treatment taken for the exacerbation. This will be reviewed by a member of the research team at each trial visit and collected at the final 12-month follow-up.

Long term follow-up will be carried out 24 months after the screening/baseline visit for participants who reach this time point within the lifetime of the trial. Data collected at this time point are mortality data, the number of emergency hospital admissions and the number of emergency hospital admissions with exacerbations of bronchiectasis. Participants will not be contacted for this follow-up, instead data will be collected from sources such as patient medical records, BronchUK [19] and/or EMBARC [20] registries or routinely collected Hospital Episode Statistics (HES) and Office of National Statistics (ONS) data.

At the end of their trial participation, participants will stop taking trial medication and continue to have their condition managed through the standard care pathway.

Table 3. Schedule of Events

**Assessment/Activity**	**Visit 1**<br>**Screening / Baseline**<br>**Day 0**	**Visit 2**<br>**1 Month Follow Up**<br>**1 month (-1/+ 2 weeks)**	**Telephone call to participant**<br>**3 days (+/- 1 day) following dispensing 2 to 6/7**	**Visit 3**<br>**6 Month Follow Up**<br>**6 months (-1/+2 weeks)**	**Telephone call to participant**<br>**3 days (+/- 1 day) following dispensing 6/7 to 13**	**Visit 4**<br>**12 Month Follow Up**<br>**12 months (+2 weeks)**	**Telephone call to participant**<br>**7 days after last dose of IMP (+ 3 days)**	**24 Month Follow up**
Written informed consent[a]	X							
Demographics	X							
Contact details (participant telephone number and address)	X	X	X	X	X			
Medical history	X							
Medication history	X							
Smoking history	X	X		X		X		
Spirometry including % predicted (FEV1 & FVC)	X	X		X		X		
Bronchiectasis Severity Index calculation	X					X		
Modified Reiff scoring of prior CT scan	X							
St George's Respiratory Questionnaire (SGRQ)	X	X		X		X		

For peer review only - http://bmjopen.bmj.com/site/about/guidelines.xhtml

Assessment/Activity	Visit 1<br>Screening / Baseline<br>Day 0	Visit 2<br>1 Month Follow Up<br>1 month (-1/+ 2 weeks)	Telephone call to participant<br>3 days (+/- 1 day) following dispensing 2 to 6/7	Visit 3<br>6 Month Follow Up<br>6 months (-1/+2 weeks)	Telephone call to participant<br>3 days (+/- 1 day) following dispensing 6/7 to 13	Visit 4<br>12 Month Follow Up<br>12 months (+2 weeks)	Telephone call to participant<br>7 days after last dose of IMP<br>(+ 3 days)	24 Month Follow up
Quality of Life – Bronchiectasis (QOL-B)	X	X		X		X		
Breathlessness – Baseline and Transition Dyspnoea Indices (BDI and TDI)	X	X		X		X		
MRC Dyspnoea Score	X	X		X		X		
Healthcare Utilisation Questionnaire	X	X		X		X		
Time and Travel Questionnaire						X		
Quality of life EQ-5D-5L	X	X		X		X		
Baseline blood test (FBC with differentials)	X							
Pregnancy test (urine, females of childbearing potential)	X							
Contraception discussion	X							
GP results request/ prior eosinophil levels extracted from medical records/ primary care datasets	X							
Eligibility confirmation	X							
Randomisation	X							
Stop pre-randomisation inhalers with the exception of SABA and commence trial inhalers	X							
Trial inhaler delivered to participant on site at face to face visit	X							
Trial inhaler delivered to participant by post (2-4 days prior to each telephone call)			X	X	X			
Confirmation of trial inhaler receipt			X	X	X			
Inhaler technique	X	X		X				
Concomitant medication		X		X		X		
Issue Patient Exacerbation Diary	X							
Participant exacerbation diary review (or reminder to use at telephone calls)		X	X	X	X	X		
Compliance check and documentation (count of returned trial inhalers and dose counts)		X		X		X		
Number of hospital admissions for bronchiectasis exacerbation		X		X		X		X
Number of emergency hospital admissions		X		X		X		X
Adverse event reporting		X	X	X	X	X	X	
Review of primary care records						X		
Mortality Data								X

For peer review only

For peer review only - http://bmjopen.bmj.com/site/about/guidelines.xhtml

Pharmacovigilance

All adverse events (AEs) will be recorded in both the participant's medical records and on the electronic Case Report Forms (eCRF) within the trial database. Adverse events that are judged by an investigator as consistent with the usual clinical pattern for patients with bronchiectasis (such as cough, increased sputum volume and/or consistency, change in sputum colour, wheeze, breathlessness, fatigue and haemoptysis) are not reportable AEs. AEs meeting the seriousness criteria (Serious Adverse Events [SAEs]) will be reported within 24 hours of awareness. Suspected Unexpected Serious Adverse Reactions (SUSAR) will be reported to the MHRA (Medicines and Healthcare products Regulatory Agency) and REC (Research Ethics Committee) within the required regulatory reporting timelines.

Emergency unblinding is available for valid medical or safety reasons where it is necessary for the treating clinician to know which treatment the participant has been receiving. Emergency unblinding should be carried out by the site Principal Investigator (PI) or another delegated medically qualified member of the research team by accessing the 24-hour web-based randomisation system.

Discontinuation and Withdrawal

Participants are free to discontinue trial treatment or withdraw from the trial at any time without detriment to their care. Participants choosing to discontinue trial treatment will be invited to complete all trial follow-up visits and assessments. Participants choosing to withdraw from the trial will not take part in any further trial activity. Data collected up to the point of withdrawal will be retained and included in analysis. Participants withdrawn from the trial after they have been randomised will not be replaced.

Data Management

Participant data will be entered by delegated site staff into the trial-specific Clinical Data Management System (CDMS) using Sealed Envelope's Red Pill (Sealed Envelope Ltd, London, UK). CDMS users have password limited access restricted to own role and site as appropriate to their delegated duties. Each potential participant will be assigned a unique sequential screening number by site staff, this unique identifier is used to add the participant to the CDMS and becomes their participant ID at the time of randomisation. Overall responsibility for data collection, quality and retention lies with the Chief Investigator who will also hold the final trial dataset. Data will be handled, computerised, stored and archived in accordance with the General Data Protection Regulation (2018), and the latest Directive on GCP (2005/28/EC). Newcastle Clinical Trials Unit (NCTU) staff monitor trial conduct and data integrity in accordance with the trial Monitoring Plan. The trial specific Data Management Plan (DMP)

For peer review only - http://bmjopen.bmj.com/site/about/guidelines.xhtml

and Data Validation Plan (DVP) include details on how data will be managed and validated throughout the trial.

Analysis

Analysis of the Primary Outcome Measure

The primary outcome - number of bronchiectasis exacerbations per participant requiring antibiotics over 12 months - will be compared between randomised groups using negative binomial regression adjusted for stratification factors. This model will be used to compute confidence intervals (CIs) to test the superiority and non-inferiority hypotheses. The two-sided 95% CI for difference in mean number of exacerbations per year between the combination of LAMA/LABA and ICS/LAMA/LABA compared to placebo will be found: the placebo vs LAMA/LABA hypothesis will be rejected if the upper limit is lower than 0. We will estimate the difference between LAMA/LABA and ICS/LAMA/LABA arms on the relative scale using the incidence rate ratio, and test whether the upper boundary of the two-sided 90% CI is lower than 1.2. We will consider the time at risk to be the time not spent in exacerbation (so that whilst a patient is in an exacerbation they are not included as at risk for another).

A secondary analysis will define the at-risk time as the entire length of follow-up for the patient. Estimates will then be adjusted for sites, the stratification factors and other baseline covariates that are known to be strongly related to outcome (e.g. age, smoking, bronchiectasis hospitalisations in year prior to trial – these will be pre-specified in the Statistical Analysis Plan (SAP)).

We will undertake a sensitivity analysis by excluding those participants who have died. If there is any indication of a differential effect on deaths by treatment, we may consider models that allow the censoring to be informative. For participants that are lost to follow up by 12 months, their information will be included in the statistical models up to the point that they are lost to follow up. If loss to follow-up is higher than 10% we will conduct sensitivity analyses to investigate the impact.

We will also explore time to first exacerbation using a Cox regression, and a recurrent events analysis to allow for subsequent exacerbations. In addition, we will utilise mortality and hospitalisations due to bronchiectasis exacerbation data collected up until 24 months to extend the modelling beyond 12 months as a sensitivity analysis.

For the primary superiority hypothesis, statistical analyses will be according to the intention to treat principle with a per protocol analysis performed as a sensitivity analysis. The per protocol analysis will exclude participants who were not compliant (at less than 75%) with their trial medication or who had a major protocol violation (to be pre-specified in the SAP).

Analysis of Secondary Outcome Measures

For peer review only - http://bmjopen.bmj.com/site/about/guidelines.xhtml

The secondary outcomes: total number of bronchiectasis exacerbations requiring hospital admission, total number of emergency hospital admissions (all causes) will each be analysed as for the primary outcome described above. Disease related health status (measured using the SGRQ, FEV1 and FVC) will be analysed using a mixed effects model adjusted for site, stratification factors, patient characteristics and/or baseline clinical variables (to be pre-specified in the Statistical Analysis Plan). Random effects for patient will be included.

Exploratory and Subgroup Analyses

Exploratory analyses will investigate the relationship between key outcomes (exacerbations and quality of life as measured by SGRQ and QOL-B) with baseline eosinophils (single level recorded at baseline), median eosinophil level (median of last 3 available recordings when not on oral steroids) and baseline BSI.

Subgroup analyses of suspected aetiology comparing idiopathic and post infectious to all other aetiologies for the key outcomes of exacerbations and quality of life (SGRQ and QOL-B) will also be carried out.

All analyses will be governed by this comprehensive SAP which will be agreed by the TSC and reviewed by the IDMEC prior to any analyses being undertaken. Unless pre-specified, a 5% two-sided significance level will be used to denote statistical significance throughout.

Economic Evaluation

Both a within-trial and model-based analysis will be undertaken. Both analyses will estimate the incremental cost per QALY gained but over different time horizons; 12-months post-randomisation (within-trial) and over the patients' life course (economic model).

The economic analysis will take the perspective of the NHS and personal and social services (PSS). Sensitivity analyses will widen the perspective to incorporate costs incurred by participants. Intervention costs will be estimated based on the manufacturers list price of the medications and inhalers provided to participants. Primary and secondary healthcare resource use will be estimated based on responses to the Health Care Utilisation Questionnaire (HCUQ) administered at baseline, 1-, 6-, and 12-months post-randomisation. These data will be combined with unit costs obtained from routine sources to estimate the total healthcare utilisation cost per participant. Additionally, patients with bronchiectasis are likely to experience AEs, treatments and hospitalisations associated with these AEs will be recorded on the eCRF and will be incorporated into the total healthcare utilisation cost per participant in a sensitivity analysis.

For peer review only - http://bmjopen.bmj.com/site/about/guidelines.xhtml

Direct (e.g. out-of-pocket payments) and indirect (e.g. time away from work) costs borne by participants will be collected via the Time and Travel questionnaire administered at 12-months post-randomisation. Similar to the healthcare costs these study specific estimates will be combined with routine sources to estimate the total costs incurred for each participant.

Health-related quality of life will be assessed based on responses to the EQ-5D-5L administered at baseline, 1-, 6-, and 12-months post-randomisation. Utility scores will be derived based on responses to the EQ-5D-5L questionnaire and the national tariff relevant at the time the study reports. QALYs will be estimated using the utility values estimated at baseline, 1-, 6-, and 12-months using the area under the curve method [26].

The total cost and QALY per participant will be summarised as the average total cost and average total QALY for each of the randomised arms. All data will be presented as point estimates of incremental costs, QALYs, cost-effectiveness. The incremental cost per QALY gained will be estimated as the difference in costs divided by the difference in QALYs if one of the arms is not dominant (i.e. less costly and more effective). The difference in costs and QALYs between the arms will be estimated using seemingly unrelated regression which can control for observed and unobserved characteristics that can affect costs and/or QALYs [27]. Uncertainty in these results, estimated using the bootstrapping technique [28], will be presented on cost-effectiveness planes and cost-effectiveness acceptability curves (CEACs).

Given that patients with bronchiectasis are expected to take their medications over their life horizon an economic model will be used to extrapolate the economic results beyond the 12-month treatment period. It is likely that this model will take the form of a Markov model but the specific form will be decided based on the within-trial results during the model development. Data to design and populate the model will be taken directly from the trial, routine sources (ONS and HES data), literature and clinical opinion. Cost and QALYs incurred after the first 12-months will be discounted at the recommended rate [29]. The model will be developed using the guidance for good practice in conceptualising an economic model [30]. Similar to the within-trial analysis these results will be presented as point estimates of costs, QALYs and cost-effectiveness. Uncertainty in the economic model will be estimated using monte carlo simulations and presented as cost-effectiveness planes and CEACs [31, 32].

Sample Size Calculations

The original sample size calculation was based on the number of exacerbations amongst a similar population in prior national audits [15, 23]. The average number of exacerbations was 2.3 per year.

For peer review only - http://bmjopen.bmj.com/site/about/guidelines.xhtml

Restricting trial entry to those who had >=3, the original inclusion criteria, (assuming a Poisson distribution) gives an average of around 3.8. We therefore assumed, given Hawthorne effect and regression to the mean that the placebo arm would have a lower mean exacerbation rate than this, and assumed a mean of 2.4 over 1 year. The sample size was chosen so that the trial was well powered to detect a clinically meaningful fall in mean exacerbation rates for bronchiectasis exacerbations to 1.9 year in LAMA/LABA and ICS/LAMA/LABA arms (approximately 20% reduction). This effect size is realistic when compared with the 20-30% reduction seen in COPD trials with dual bronchodilators/ triple therapy and is accepted as clinically meaningful. Although likely studying a different sub population in bronchiectasis, a recent inhaled antibiotic trial in bronchiectasis reduced exacerbations by 39% [4].

For 90% power (two-sided 5% significance level) to conclude that LAMA/LABA is more effective than placebo with the above parameters, we have calculated (assuming large-sample approximation of the Poisson distribution) a sample size of 600 participants is needed, randomised 240:240:120 between LAMA/LABA, ICS/LAMA/LABA and placebo. This allows for a 5% loss to follow-up. This represents a conservative retention rate compared to over 95% observed in the NIHR HTA TWICS study with similar pragmatic design and limited patient burden. This calculation assumes a difference between placebo and LABA/LAMA of 0.5 exacerbations per year (21% relative reduction).

If superiority of LAMA/LABA vs placebo is concluded, we will then test non-inferiority of LAMA/LABA against ICS/LAMA/LABA. The sample size will give 90% power (one-sided 5% type I error) with a 0.38 non-inferiority margin (reflecting 20% of the assumed LAMA/LABA rate).

In the early stages of the trial it was found that exacerbation rates in pre-baseline periods were lower than they had been historically, mostly due to the presumed effect of COVID-19 restrictions. The inclusion criteria were therefore updated in protocol V5.0 (see Amendments). A recalculation of the power of the trial was conducted assuming that there would be lower exacerbation rates in the follow-up period. We assumed that the placebo exacerbation rate would be an average of 1.9 per year. We recalculated the power for two scenarios: 1) assuming that the same absolute difference (0.5) between LAMA/LABA vs placebo and the same non-inferiority margin (0.38) as previously; 2) the same relative difference (21%) between LAMA/LABA vs placebo and relative non-inferiority margin (20%) as before. Table 4 shows the power of the trial to conclude non-inferiority and superiority.

Table 4. Power Calculations

Scenario	Mean exacerbation rate	NI Margin	Power*

For peer review only - http://bmjopen.bmj.com/site/about/guidelines.xhtml

	Placebo	ICS/LAMA/LABA (Intervention)	LAMA/LABA (Active control)	(relative to active control)	Non-inferiority	Superiority
Presented in original grant application	2.4	1.9	1.9	0.38 (20%)	90.3%	89.8%
Updated inclusion criteria (same absolute differences)	1.9	1.4	1.4	0.38 (26.7%)	96.3%	95.8%
Updated inclusion criteria (same relative differences)	1.9	1.5	1.5	0.3 (20%)	83.4%	83.4%

*Note this is assuming analytical formulae, with simulations giving consistent but slightly higher powers

Amendments

A number of amendments have been approved for the trial. Of note is Amendment 06, a substantial amendment for which the primary purpose was to change eligibility criteria and to extend the pilot phase of the trial.

Initially the protocol required that patients experience 3 exacerbations of bronchiectasis within the preceding 12 months to be eligible for the trial. However, this criterion posed a challenge to recruitment given that the biology of bronchiectasis has changed following the shielding behaviour of patients with bronchiectasis during the COVID-19 pandemic. These behaviours have resulted in a reduction in exacerbations being observed in the U.K. [33] and hence, a reduction in the pool of potentially participants for the trial. In discussion with the oversight committees and funder for the trial it was agreed to extend the potential participant pool for the trial by changing this eligibility criterion to include patients who have experienced 2 or more exacerbations within any 12 month period at any time in the preceding two years.

For peer review only - http://bmjopen.bmj.com/site/about/guidelines.xhtml

As a reflection of the slower recruitment and the challenges faced by sites setting up a respiratory trial during COVID-19, it was agreed to extend the pilot phase of the trial from 6 to 12 months.

Patient and Public Involvement (PPI)

PPI were involved in the trial design and are co-applicants for the research. Independent PPI have oversight of the trial and results dissemination as members of the TSC.

Trial Status

The DIBS trial recruitment opened on the 29th July 2021 and closed on the 21st October 2022, the last patient last visit will be in October 2023. This manuscript is based in protocol version 6.0 dated 15th July 2022.

ETHICS AND DISSEMINATION

The trial received a favourable ethical opinion from the North East – Newcastle & North Tyneside 2 Research Ethics Committee (reference: 21/NE/0020) in March 2021. Trial results will be disseminated in peer reviewed publications, at national and international academic conferences and in the NIHR Health Technology Assessments (HTA) journal. Results will also be disseminated to the public and participants using lay language.

AUTHOR CONTRIBUTIONS

ADS is the trial Chief Investigator and senior author. AS, RM, CH, JH, ADS prepared the funding application. MM, NW, TH, LS, AS, RM, JW, LT, AA, MA, RJ, VH, RL, SC, AW, JC, AH, CH, JH and ADS have contributed to protocol development and amendments and have revised the manuscript and given approval for the final version. NW, JW and SC have advised on trial design and provide statistical oversight and analysis. TH and LT have advised on trial design and provide health economic oversight and analysis. MA and AW provide IMP oversight, MM, LS, AS, RM, AA, RJ, VH and RL provide trial and database design and management and trial monitoring.

FUNDING STATEMENT

National Institute for Health Research (NIHR) Health Technology Assessment (HTA), funder reference NIHR127460.

The views expressed are those of the author(s) and not necessarily those of the NIHR or the Department of Health and Social Care.

COMPETING INTERESTS STATEMENT

For peer review only - http://bmjopen.bmj.com/site/about/guidelines.xhtml

ADS has received speakers fees or advisory board fees from AstraZeneca, Bayer, GSK, Insmed, Novartis, Gilead and Zambon and has received grants from AstraZeneca, GSK, Novartis and the US COPD Foundation.

FULL REFERENCES

1. Pasteur, M.C., et al., *An investigation into causative factors in patients with bronchiectasis.* Am J Respir Crit Care Med, 2000. **162**(4 Pt 1): p. 1277-84.
2. Shoemark, A., L. Ozerovitch, and R. Wilson, *Aetiology in adult patients with bronchiectasis.* Respir Med, 2007. **101**(6): p. 1163-70.
3. O'Leary, C.J., et al., *Relationship between psychological well-being and lung health status in patients with bronchiectasis.* Respir Med, 2002. **96**(9): p. 686-92.
4. Wilson, C.B., et al., *Validation of the St. George's Respiratory Questionnaire in bronchiectasis.* Am J Respir Crit Care Med, 1997. **156**(2 Pt 1): p. 536-41.
5. De-Soyza, A., et al. *Earlier onset of fatigue in bronchiectasis as compared with COPD*. in *European Respiratory Society Annual Congress*. 2010. Newcastle University.
6. Hester, K.L., et al., *Fatigue in bronchiectasis.* Qjm, 2012. **105**(3): p. 235-40.
7. Patel, I.S., et al., *Bronchiectasis, exacerbation indices, and inflammation in chronic obstructive pulmonary disease.* Am J Respir Crit Care Med, 2004. **170**(4): p. 400-7.
8. Shahab, L., et al., *Prevalence, diagnosis and relation to tobacco dependence of chronic obstructive pulmonary disease in a nationally representative population sample.* Thorax, 2006. **61**(12): p. 1043-7.
9. Roberts, H.J. and R. Hubbard, *Trends in bronchiectasis mortality in England and Wales.* Respir Med, 2010. **104**(7): p. 981-5.
10. Loebinger, M.R., et al., *Mortality in bronchiectasis: a long-term study assessing the factors influencing survival.* Eur Respir J, 2009. **34**(4): p. 843-9.
11. Chalmers, J.D., et al., *The bronchiectasis severity index. An international derivation and validation study.* Am J Respir Crit Care Med, 2014. **189**(5): p. 576-85.
12. Seitz, A.E., et al., *Trends and burden of bronchiectasis-associated hospitalizations in the United States, 1993-2006.* Chest, 2010. **138**(4): p. 944-9.
13. Quint, J.K., et al., *Changes in the incidence, prevalence and mortality of bronchiectasis in the UK from 2004 to 2013: a population-based cohort study.* Eur Respir J, 2016. **47**(1): p. 186-93.
14. Kelly, M.G., S. Murphy, and J.S. Elborn, *Bronchiectasis in secondary care: a comprehensive profile of a neglected disease.* Eur J Intern Med, 2003. **14**(8): p. 488-492.
15. Hill, A.T., et al., *British Thoracic Society national bronchiectasis audit 2010 and 2011.* Thorax, 2012. **67**(10): p. 928-30.
16. *Global Initiative for Asthma. Global Strategy for Asthma Management and Prevention. Available from:* www.ginasthma.org. 2022.
17. *Global Initiative for Chronic Obstructive Lung Disease - goldcopd.org*. 2022.
18. Chan, A.W., et al., *SPIRIT 2013 explanation and elaboration: guidance for protocols of clinical trials.* Bmj, 2013. **346**: p. e7586.
19. De Soyza, A., et al., *BronchUK: protocol for an observational cohort study and biobank in bronchiectasis.* ERJ Open Res, 2021. **7**(2).
20. Chalmers, J.D., et al., *The European Multicentre Bronchiectasis Audit and Research Collaboration (EMBARC): experiences from a successful ERS Clinical Research Collaboration.* Breathe (Sheff), 2017. **13**(3): p. 180-192.
21. Reiff, D.B., et al., *CT findings in bronchiectasis: limited value in distinguishing between idiopathic and specific types.* AJR Am J Roentgenol, 1995. **165**(2): p. 261-7.
22. Quittner, A.L., et al., *A preliminary quality of life questionnaire-bronchiectasis: a patient-reported outcome measure for bronchiectasis.* Chest, 2014. **146**(2): p. 437-448.

For peer review only - http://bmjopen.bmj.com/site/about/guidelines.xhtml

23. Devlin, N.J., et al., *Valuing health-related quality of life: An EQ-5D-5L value set for England.* Health Econ, 2018. **27**(1): p. 7-22.
24. *Standardized Questionaries on Respiratory Symptoms.* Br Med J, 1960. **2**(5213): p. 1665.
25. Mahler, D.A., et al., *The measurement of dyspnea. Contents, interobserver agreement, and physiologic correlates of two new clinical indexes.* Chest, 1984. **85**(6): p. 751-8.
26. Matthews, J.N., et al., *Analysis of serial measurements in medical research.* Bmj, 1990. **300**(6719): p. 230-5.
27. Fiebig, D.G., *A Companion to Theoretical Econometrics*. Seemingly Unrealted Regression., ed. B.H. Baltagi. 2003, Malden, MA, USA: Blackwell Publishing Ltd.
28. Barber, J.A. and S.G. Thompson, *Analysis of cost data in randomized trials: an application of the non-parametric bootstrap.* Stat Med, 2000. **19**(23): p. 3219-36.
29. National Institute for, H. and E. Care, *NICE Process and Methods Guides*, in *Guide to the Methods of Technology Appraisal 2013*. 2013, National Institute for Health and Care Excellence (NICE): London.
30. Philips, Z., et al., *Review of guidelines for good practice in decision-analytic modelling in health technology assessment.* Health Technol Assess, 2004. **8**(36): p. iii-iv, ix-xi, 1-158.
31. Black, W.C., *The CE plane: a graphic representation of cost-effectiveness.* Med Decis Making, 1990. **10**(3): p. 212-4.
32. Fenwick, E., K. Claxton, and M. Sculpher, *Representing uncertainty: the role of cost-effectiveness acceptability curves.* Health Econ, 2001. **10**(8): p. 779-87.
33. Crichton, M.L., A. Shoemark, and J.D. Chalmers, *The Impact of the COVID-19 Pandemic on Exacerbations and Symptoms in Bronchiectasis: A Prospective Study.* Am J Respir Crit Care Med, 2021. **204**(7): p. 857-859.

For peer review only

For peer review only - http://bmjopen.bmj.com/site/about/guidelines.xhtml

TO BE PRINTED ON TRUST HEADED PAPER

Dual Bronchodilators in Bronchiectasis Study

DIBS

Participant Information Sheet

Version 4.0: 18th February 2022

INVITATION

We are inviting you to take part in our research study called DIBS. Please read the following information to help you decide if you want to take part. We would like you to understand why this research is being done and what it would involve for you. You don't have to decide straight away and you can talk to your friends and family or GP. A member of our team will go through this information sheet with you and answer any questions you may have. The study is being funded by the National Institute for Health Research (NIHR) which is the Health Research arm of the NHS.

TRIAL SUMMARY

- This study is looking at the use of bronchodilator inhalers for preventing exacerbations of bronchiectasis. Bronchodilators are a type of medication taken that relax the muscles in the lungs and widen the airways to make breathing easier. The inhalers being tested are a dual bronchodilator inhaler and a dual bronchodilator combined with corticosteroid inhaler (corticosteroid inhalers are also sometimes referred to as steroid inhalers). You will stop taking any inhaler(s) you are already on with the exception of your quick-acting inhaler (such as salbutamol). You will be randomised to either one of the two treatment inhalers or the placebo inhaler (a 'dummy' treatment). You will have four times the chance of

For peer review only - http://bmjopen.bmj.com/site/about/guidelines.xhtml

being on either one of the treatment inhalers as on the placebo inhaler. You and the study doctor will not know which inhaler you are taking.

- You will use your study inhaler once a day for 12 months (365 days).
- You will have 4 hospital visits for the study over the 12 months. These visits will take place alongside your normal hospital appointments or as additional visits. During these visits you will have some tests and complete some questionnaires.
- You will have a telephone call approximately every month to check that you have received your replacement inhaler through the post.
- You will be asked to complete an exacerbation diary each week for 12 months to record any bronchiectasis exacerbations.
- The study will include 600 bronchiectasis patients at over 20 hospitals.
- Your participation is voluntary, you will be part of a national effort to better understand bronchiectasis and its treatment.

Please read the following information for further details about the study if you are interested in taking part.

WHY IS DIBS NEEDED?

There are over 300,000 patients in the UK who suffer from bronchiectasis. There are currently no licensed treatments and no cures, which means there are large differences in care. Patients with bronchiectasis are normally treated with inhaled medicines approved for asthma and smoking-related chronic obstructive pulmonary disease (COPD) without any proof they work in bronchiectasis. We do not know which inhaled treatment is best for bronchiectasis; this is why we are conducting this research study. We will look at whether a dual bronchodilator inhaler or a dual bronchodilator plus a corticosteroid inhaler can reduce the number of bronchiectasis exacerbations (or flare ups) requiring antibiotic or steroid treatment compared to no treatment. An exacerbation (or flare up) is a sustained deterioration in your condition that would lead you to think about seeing your healthcare team or starting emergency packs.

For peer review only - http://bmjopen.bmj.com/site/about/guidelines.xhtml

The safety and side effects of these inhalers in other respiratory conditions are well known.

WHY AM I BEING INVITED TO TAKE PART?

You are being invited to take part in this research study because you have been diagnosed with a respiratory condition called bronchiectasis. You are also being invited because within the last 2 years you have had 2 or more exacerbations (or flare ups) within 12 months of each other which have required antibiotic or steroid treatment.

DO I HAVE TO TAKE PART?

No, it is up to you to decide whether you want to join the study. If you agree to take part, we will ask you to complete a consent form. If you choose not to, you will continue to get the standard care arranged by your doctor.

If you agree to take part, you can change your mind at any time. You can stop taking study medication and carry on with the questionnaires and study visits, or you can withdraw from the study completely. You do not have to give a reason, but it is helpful to the study if you do so ways it can be improved can be understood. If you decide to withdraw completely, data collected up to this point will be retained for analysis. This will not affect the care that you receive.

WHAT WILL GIVING CONSENT MEAN FOR ME?

By signing a consent form, this means that you fully understand what taking part in the study means for you. That's why it is really important that you take as much time as you want to read this information sheet and ask lots of questions.

WHAT WOULD TAKING PART INVOLVE?

We will ask you to attend 4 hospital visits:

- Initial visit
- 1 month follow-up visit
- 6 month follow-up visit
- 12 month follow-up visit

For peer review only - http://bmjopen.bmj.com/site/about/guidelines.xhtml

These visits are not part of your standard care but could take place alongside your standard care visits if it is possible. The diagram on page 6 shows the details of the study hospital visits and what will take place at each visit.

During your study hospital visits you will be asked to complete some questionnaires. Some will be completed by you and others will be completed by a member of the study team who will ask you questions. These questionnaires are used to assess any breathlessness you may have, the impact of your health on your usual activities, your mental health and quality of life. There are also questionnaires about how often you have had to access healthcare for routine appointments and urgent care, any costs you have incurred accessing healthcare and time taken to travel to access healthcare. We will use this to compare the overall healthcare costs between each treatment arm in the study.

A blood sample and urine sample (if needed for a pregnancy test) will be taken at the first (baseline) study visit. The blood sample is taken to look at reasons why patients may respond differently to the inhalers and as a baseline test for safety reasons. We will collect approximately 1 teaspoon (5 mL) full of blood. These samples will be analysed in the local hospital laboratory and will be destroyed after analysis. No samples (blood or urine) will be stored for the purposes of this study.

All of your study visits and procedures will be carried out in line with your study hospital's COVID-19 policies and procedures. These will be explained to you when your appointments are arranged.

For the 12 months of the study you will be asked to complete an exacerbation diary each week. You will record any exacerbations of your bronchiectasis in the last week and any antibiotic or steroid treatment.

We will arrange delivery of your inhalers in between the face to face visits, at a time convenient for you. These will be delivered by post or courier, depending on your local research team's policy.

You will have phone calls approximately every month with a member of the research team to check:

- That you have received your replacement inhaler in the post
- The current dose count on your inhaler

For peer review only - http://bmjopen.bmj.com/site/about/guidelines.xhtml

- Whether your contact details have changed
- Whether you have any planned travel/holidays that may affect the timing of the subsequent inhaler postings
- Completion of your exacerbation diary each week
- Whether you have had any adverse events (new or worsened signs or symptoms) or any hospital admissions

You will have to stop taking any current inhalers, with the exception if you have a blue (Salbutamol) inhaler used to relieve a sudden attack of breathlessness or wheezing. If you get this sort of attack you must continue to use a quick-acting inhaler (such as salbutamol).

You will have a phone call between 7 and 10 days after your last dose of the study inhaler. This call is to check whether you have had any adverse events (new or worsened signs or symptoms) or any hospital admissions since your most recent study visit.

We are interested in the long-term health of participants in this study. With your permission we will continue to ask for information relating to your health following the end of your participation in the study for up to one year (referred to in the study as 24 month follow up data), depending on the date you join the study. You will not need to visit hospital or be called by the research team for this. Instead, we will ask for information from Bronchiectasis Registries (BronchUK [The United Kingdom Bronchiectasis Registry] and EMBARC [European Multicentre Bronchiectasis Audit and Research Collaboration]) if you have already given consent to these projects for health follow ups. Or, we may ask for information from routinely collected sources such as NHS Digital (including Hospital Episodes Statistics [HES]) and the Office for National Statistics (ONS) (ONS/HES data will not be collected for participants with trial visits at Scottish hospitals). Members of the hospital research team may also review your medical records. The data collected will only include information about you for up to one year after your last dose of study medication, but, because data is requested in batches from HES, ONS and other databases, the request for your data may be made up to two years after your participation in the study has ended.

For peer review only - http://bmjopen.bmj.com/site/about/guidelines.xhtml

Figure 1. Hospital visit details

SCREENING/BASELINE VISIT – VISIT LENGTH 2-3 HOURS

- Written informed consent
- Eligibility for the trial confirmed
- Medical history, smoking history and medication review
- Urine pregnancy test (if needed)
- Blood tests
- Questionnaires
- Lung function tests
- Randomisation to treatment arm
- Receive first inhaler
- Test of inhaler technique

1 AND 6 MONTH FOLLOW UP – VISIT LENGTH 1-2 HOURS

- Check contact details
- Medication and smoking review
- Lung function tests
- Questionnaires
- Test of inhaler technique and used inhaler return
- Review of exacerbation diary and any adverse events including hospital visits

12 MONTH FOLLOW UP – VISIT LENGTH 1-2 HOURS

- Medication and smoking review
- Lung function tests
- Questionnaires
- Used inhaler return
- Review of exacerbation diary and any adverse events including hospital visits

For peer review only - http://bmjopen.bmj.com/site/about/guidelines.xhtml

WHAT TREATMENT WOULD I BE ON?

The active treatments we are testing have already been tested and shown to work in the smoking related lung condition called COPD. The inhalers are already in wide use across the UK for that condition. If you decide to take part in the study, you will be randomised by computer to one of three treatment groups: each arm has ONLY one inhalation from one inhaler per day.

- Dual inhaler (dual bronchodilator; Umeclidinium/Vilanterol)
- Triple inhaler (dual bronchodilator plus a corticosteroid; Umeclidinium/ Vilanterol & Fluticasone Furoate)
- Placebo inhaler (a 'dummy' treatment)

The dual inhaler contains 2 active medicines: Umeclidinium and Vilanterol; these both belong to a group of medicines called bronchodilators. Bronchodilators widen the muscles around the airways in the lungs which makes it easier for air to get in and out of the lungs. When used regularly, bronchodilators can help to control breathing difficulties.

The triple inhaler contains 3 active medicines: Umeclidinium, Vilanterol and Fluticasone Furoate. Umeclidinium and Vilanterol are the same bronchodilator medicines that are in the dual inhaler. The Fluticasone Furoate is a steroid medicine. Bronchodilators widen the muscles in the lungs and steroids reduce swelling and irritation in the small air passages which makes it easier for air to get in and out of the lungs. When used regularly bronchodilators and steroids can help to control breathing difficulties.

The placebo inhaler does not contain any active medicines; it contains a dry powder that should not have any effect on you.

The dual and triple inhalers have already been tested in large research studies for safety, side effects, and effectiveness in treating COPD and have been licenced for use in the treatment of COPD. The DIBS study is a phase 3 clinical trial. A phase 3 clinical trial is a study which includes a large number of patients to look at whether a treatment works well. We hope to see in DIBS whether bronchodilators and/or bronchodilators and steroids, which have been used already to treat COPD, are effective treatments for patients with bronchiectasis.

For peer review only

For peer review only - http://bmjopen.bmj.com/site/about/guidelines.xhtml

The inhalers all look the same. You will have four times the chance of getting an active inhaler as being in the placebo group (i.e. if there are 5 participants, 2 will have the dual inhaler, 2 will have the triple inhaler, and 1 will have the placebo inhaler).

Which inhaler you are given will be completely random. You and the healthcare professionals involved in the study will not know which type of inhaler you are allocated to.

You will be prescribed study inhalers for 12 months (365 days in total). Each inhaler has 30 doses and you need to take one puff of your inhaler every day for 12 months (365 days in total). You should try to take your inhaler at the same time every day. You will be given your first inhaler at your screening/baseline hospital visit and then receive replacement inhalers by post. The inhalers will be sent by recorded delivery and you may need to sign to confirm you have received them. On study visit days, do **not** use your inhaler before you arrive in hospital. You will be asked to bring your current inhaler to hospital and to take your daily dose whilst attending hospital. This is to check whether you are using your inhaler correctly.

PLEASE KEEP ALL OF YOUR USED INHALERS

It is vitally important that you keep hold of all your study inhalers, even the empty ones. You will be asked to return all inhalers at your next study visit so that the number of doses you have used can be checked and recorded.

WHAT ARE THE POSSIBLE BENEFITS OF TAKING PART?

Patients with other lung conditions where these inhalers are already proven to work reported better lung function, better quality of life and less exacerbations (or flare ups). We cannot promise this study will help you directly but the information we get from this study may help to improve treatment for people with bronchiectasis in the future. If you want to find out more about taking part in research studies you can visit the NHS Choices website https://www.nhs.uk/conditions/clinical-trials/

For peer review only - http://bmjopen.bmj.com/site/about/guidelines.xhtml

EXPENSES

Your travel expenses (up to £25 per visit) for each hospital visit will be paid for by the study. Alternatively, transport may be arranged for you if your local hospital is able to offer this. In addition, should you prefer to contact the study team for your monthly telephone call rather than the study team telephoning you, the costs of these telephone calls will be paid for by the study.

Your study team will manage any payments to reimburse costs to you and you may be asked to provide receipts for your travel.

WILL MY GP KNOW THAT I'M TAKING PART IN THE STUDY?

Yes, we will send a letter to your GP to inform them that you are taking part in this study, and a copy will be filed in your hospital notes. This is so that your medical records at your GP practice and in hospital contain documentation that you are taking part in a clinical study. Any test results from taking part in this study will also be added to your medical records. Your GP will be asked to inform the study team of any adverse reactions to the study medication or emergency hospital admissions.

Your GP will not know if you have received active treatment or placebo.

WHAT ARE THE POSSIBLE DISADVANTAGES AND RISKS OF TAKING PART?

We want you to be safe in this study at all times, but all medical treatments carry some risk. The inhalers used in this study are widely used by NHS patients with respiratory conditions, and there are some known side effects.

If you react badly to the inhaler your study doctor will be able to change your medication and treat you to try to ease your symptoms. If they need to find out which treatment you are taking (one of the two active treatments or placebo), this information is available in cases of emergency.

If you feel unwell during your time in the study then please contact your research team or study doctor.

POSSIBLE SIDE EFFECTS

For peer review only - http://bmjopen.bmj.com/site/about/guidelines.xhtml

As with most medicines the study inhalers do have some side effects which are listed in the table below. As these drugs are widely used in COPD our experience is that very few of these side effects are common causes for patients to stop their inhalers. If you are assigned to the triple inhaler group there may be a higher risk of you getting pneumonia. This is because the steroid inhalers have been shown to make the risk of getting pneumonia higher in patients with COPD. Pneumonia is when there is swelling of the tissue in one or both lungs, usually caused by an infection. The steroid inhaler reduces COPD exacerbations a lot more than the risk of pneumonia it brings to these patients. If you experience any of the side effects listed in the table below, it is important that you let your study team know straight away. The study team can talk to you more about what these mean if you are unsure about any of them. These side effects often are temporary.

**Frequency**	**Dual inhaler (dual bronchodilator)**	**Triple inhaler (dual bronchodilator and corticosteroid)**
**Common or very common**	▪ Increased risk of infection<br>▪ Cough<br>▪ Throat pain<br>▪ Constipation<br>▪ Dry mouth<br>▪ Headache	▪ Increased risk of infection<br>▪ Headache<br>▪ Cough<br>▪ Throat pain<br>▪ Constipation<br>▪ Joint pain<br>▪ Back pain<br>▪ Thrush in the mouth and throat
**Uncommon**	▪ Rash<br>▪ Tremor<br>▪ Altered taste<br>▪ Abnormal heart rhythms<br>▪ Palpitations<br>▪ Hoarse voice	▪ Abnormal heart rhythms<br>▪ Dry mouth<br>▪ Bone fracture<br>▪ Altered taste<br>▪ Hoarse voice<br>▪ Chest infection
**Rare or very rare**	▪ Swelling<br>▪ Anaphylaxis<br>▪ Blurred vision<br>▪ Glaucoma	▪ Swelling<br>▪ Anaphylaxis<br>▪ Skin rash

For peer review only - http://bmjopen.bmj.com/site/about/guidelines.xhtml

	▪ Pressure inside the eye<br>▪ Tightening of air passages (sometimes severe)<br>▪ Urinary problems	
**Frequency unknown***	▪ Dizziness	▪ Blurred vision

* the frequency of these side effects are unknown as there is not enough data available at the moment to estimate the frequency.

You could be randomised to receive the placebo 'dummy' treatment. If you get the placebo treatment it could be the case that you experience more exacerbations than on your current treatment/inhaler. You and the study team will not know which treatment you are receiving.

PREGNANCY AND CONTRACEPTION

As we do not know if the study medicines will harm an unborn baby those planning to become pregnant (or their partner becoming pregnant) will be excluded from the study.

To prevent pregnancy during the study, all participants of childbearing potential must use contraception from the day that consent is given to take part in the study up until 7 days after the last dose of study medication is taken.

For male participants contraceptive methods include:

- condom
- practice true abstinence in line with preferred and usual lifestyle

For female participants contraceptive methods include:

- combined hormonal contraception (oral, intravaginal, transdermal)
- progestogen only hormonal contraception (oral, injectable, implantable)
- intrauterine device (IUD)
- intrauterine hormone-releasing system (IUS)
- vasectomised partner
- bilateral tubal occlusion

For peer review only - http://bmjopen.bmj.com/site/about/guidelines.xhtml
For peer review only

- practice true abstinence in line with preferred and usual lifestyle

If you or your partner become pregnant during the course of the study, you must tell your study doctor **immediately** so appropriate action can be discussed.

If you do become pregnant during the study, you must stop taking your study inhaler. We will ask you to return your study inhaler at your next follow-up visit.

We will monitor your pregnancy carefully and will ask if we can collect some information on the health of your baby when it is born. A children's doctor will also be asked to check your baby carefully when it is born.

If your partner becomes pregnant during the study, we will ask them to sign a consent form. This will allow the study team to collect safety information about their pregnancy and their baby.

BREASTFEEDING

If you are breastfeeding you will not be allowed to take part in the study. This is because we don't know if the study medicine is passed into breastmilk or if it is safe in babies.

WHAT WILL HAPPEN TO ME WHEN THE STUDY ENDS?

You will stop taking the study inhalers and you will continue to receive standard care like any other patient with your condition, under the care of your respiratory doctor. You and your respiratory doctor can discuss which treatment would be best for you at that time. You and your doctors will not find out which inhaler you were taking during the study.

WHAT IF SOMETHING GOES WRONG?

If you have a concern about any aspect of this study, you can speak to a member of the study team who will do their best to answer your questions. Further contact details are included at the end of this information sheet. If you are still unhappy and wish to raise your concerns with someone who is not directly involved in your care, you can contact <site to localise with local details such as PALS (or equivalent) phone number and email address>

In the unlikely event that you are harmed during the study and this is due to someone's negligence (they were careless) you may have grounds for legal

For peer review only - http://bmjopen.bmj.com/site/about/guidelines.xhtml

action and compensation, but you may need to meet your own legal costs. NHS Indemnity does not offer no-fault compensation (for harm that is not anyone's fault).

The Newcastle Clinical Trials Unit, part of Newcastle University, are managing the study on behalf of the study NHS Sponsor. Newcastle University also have insurance arrangements in place to cover Newcastle University staff involved in designing and managing the DIBS study.

WHO IS ORGANISING AND FUNDING THE STUDY?

The doctor in charge of the study (the Chief Investigator) is Professor Anthony De Soyza, a Respiratory Consultant. He is based in Newcastle upon Tyne.

Study Sponsor: Newcastle upon Tyne Hospitals NHS Foundation Trust. The study Sponsor has responsibility for the study. The study is managed by the Newcastle Clinical Trials Unit, Newcastle University, on behalf of the Sponsor.

Study Funder: National Institute for Health Research Health Technology Assessment programme. This body is funded by the UK government to carry out research for the benefit of the NHS and its patients.

GlaxoSmithKline plc, a pharmaceutical company, have supported the study by providing their inhalers to the study team free of charge.

WHO HAS REVIEWED THIS STUDY?

This study was reviewed and approved by the Research Ethics Committee (North East- Newcastle & North Tyneside 2), the Health Research Authority (HRA) and the Medicines and Healthcare products Regulatory Agency (MHRA). The MHRA are responsible for approving all studies involving medicines. The Newcastle Upon Tyne Hospitals NHS Foundation Trust has reviewed all the study documentation and assessed the risks of this study as part of their responsibility, as study Sponsor. This is to ensure that we are not doing anything harmful to you during the study and that your data is collected safely and stored securely. Bronchiectasis patients helped to design this study. They have also looked at the patient information sheet, the consent form and the exacerbation diary.

WHO IS PROVIDING THE STUDY INHALERS?

For peer review only - http://bmjopen.bmj.com/site/about/guidelines.xhtml

The inhalers for all three treatment groups in this study have been provided free of charge by GlaxoSmithKline plc, a pharmaceutical company. They recognise patients with bronchiectasis have no licenced therapies.

WHAT IF RELEVANT NEW INFORMATION BECOMES AVAILABLE?

If, during the course of the study, new information becomes available that is relevant to you, we will tell you about it. We will discuss whether you should or would like to stop the study treatment or withdraw from the study.

WHAT WILL HAPPEN TO THE RESULTS OF THE RESEARCH STUDY?

- The results will be written in medical journals and presented in meetings to other doctors, nurses, researchers and patients.
- A report will be written for the study funder and put on their website.
- All study data that is published will be anonymous. Your identity will always be protected.
- The results will be available at the end of the study through publications, in the wider press and directly to patient groups. You will be able to request a copy of the summary of the results from your study hospital.
- Fully anonymised data may be made available to other researchers to help inform other research studies.

WILL MY TAKING PART IN THIS STUDY BE KEPT CONFIDENTIAL?

Yes. All of the information collected in the study will be entered on computers that are kept secure and password protected.

- You will be given a unique study identification number (called a Participant ID Number) instead of your name being written on study documents. The study team at your hospital will be able to link this number back to you using your date of birth, name, and NHS number (or other nations equivalent). A restricted number of the study team at the Newcastle Clinical Trials Unit may have access to your date of birth, Participant ID number and NHS numbers together. This is for the purpose of requesting the 24 month follow up data, depending on when you join the study.

For peer review only - http://bmjopen.bmj.com/site/about/guidelines.xhtml

- The study team at your hospital will have access to your information during the study to organise planned visits as well as for ongoing safety. They will be in contact with you about receipt of your replacement inhalers and your health
- Your hospital pharmacy/study team will have access to your contact details for posting your replacement study medication
- Your contact details will never be shared with anyone outside of the study with the exception of Royal Mail or an alternative postal/courier service used by your local hospital. You will be asked to consent to the postal service your local hospital uses to have access to your contact details so that they can deliver your replacement inhalers to your home address. The postal services will not know you are in the study just that they need to deliver a package to you
- You will not be named in any results, reports or on websites
- Very occasionally, information might be given during the study that we would have a legal obligation to pass on to others (for instance information which suggested you or others were at risk of harm). In this case, confidentiality would be broken so that we could pass this information to the relevant people. You would be informed of this
- At the end of the study, all study information will be kept in a secure storage area (this is called archiving) for at least 5 years. This makes sure any queries about the running of the study have been answered. All information will be held securely to make sure we protect your confidentiality, after which it will be safely destroyed
- If there are any serious adverse events, we would send details of them to the government medicines agency (MHRA); only your participant ID number would be sent to them. This information may also be sent to researchers outside of the United Kingdom (UK) in the European Economic Area (EEA) who are involved in overseeing the study.
- Some parts of your medical records and the data collected for the study may be looked at by authorised persons from the MHRA, Sponsor (Newcastle Hospitals NHS Foundation Trust) and or the Newcastle Clinical Trials Unit to check that the study is being conducted to the correct standards. All will have a duty of confidentiality to you as a research participant.

For peer review only - http://bmjopen.bmj.com/site/about/guidelines.xhtml

- GlaxoSmithKline plc have asked for de-identified data (age, ethnicity, sex at birth (male/female), site number and disease status and new participant identifier) to be shared with them. This is to allow them to complete their own analyses. This data may be used by anyone in the international company and may leave the European Economic Area (EEA).
- In order to learn about the long-term health of participants in this study your NHS number (or other nations equivalent) may be sent to the Newcastle Clinical Trials Unit, part of Newcastle University. Your information would be transferred and stored securely with access limited to the people that need to know this information.

HOW WILL WE USE INFORMATION ABOUT YOU?

We will need to use information from you, from your medical records and from your GP for this research project.

This information will include your initials, date of birth, NHS number (or other nations equivalent), name and contact details. People will use this information to do the research or to check your records to make sure that the research is being done properly.

People who do not need to know who you are will not be able to see your name or contact details. Your data will have a code number, your unique Participant ID, instead.

We will keep all information about you safe and secure.

Once we have finished the study, we will keep some of the data so we can check the results. We will write our reports in a way that no-one can work out that you took part in the study.

WHAT ARE YOUR CHOICES ABOUT HOW YOUR INFORMATION IS USED?

- You can stop being part of the study at any time, without giving a reason, but we will keep information about you that we already have.

For peer review only - http://bmjopen.bmj.com/site/about/guidelines.xhtml

- If you choose to stop taking part in the study, we would like to continue collecting information about your health from central NHS (or other nations equivalent) records, your hospital and your GP. If you do not want this to happen, tell us and we will stop.
- We need to manage your records in specific ways for the research to be reliable. This means that we won't be able to let you see or change the data we hold about you.

WHERE CAN YOU FIND OUT MORE ABOUT HOW YOUR INFORMATION IS USED?

You can find out more about how we use your information

- at www.hra.nhs.uk/information-about-patients/
- our leaflet available from https://www.newcastle-hospitals.nhs.uk/help/privacy/privacy-notice-for-patients/
- by asking one of the research team
- by sending an email to the Sponsor Data Protection Officer at nuth.dpo@nhs.net
- by ringing the Newcastle upon Tyne Hospital Data Protection Officer on 0191 223 1474

FURTHER INFORMATION AND CONTACT DETAILS

If you have any further questions or would like any further information about the study or the rights of participants, please feel free to contact the people below.

They are also who you or your doctor should contact in the event of an emergency, if your study participation is in any way involved.

[LOCAL CONTACT DETAILS]

Thank you for reading this information sheet.

For peer review only - http://bmjopen.bmj.com/site/about/guidelines.xhtml

Newcastle University

Print on Trust headed paper

Dual Bronchodilators in Bronchiectasis Study (DIBS)

DIBS

INFORMED CONSENT FORM

V2.0 29th March 2021

Participant ID: [ ][ ][ ][ ][ ][ ]

Principal Investigator: ___________________________________

		**Please <u>INITIAL</u> the boxes if**
1.	I confirm that I have read the Participant Information Sheet dated __/___/____ version ___ for the above study and that I have had the opportunity to consider the information, ask questions and have had these answered satisfactorily.	
2.	I understand that my participation is voluntary and that I am free to withdraw at any time without giving any reason, without my medical care or legal rights being affected.	
3.	I consent to receiving the study medication outlined in the Participant Information Sheet.	
4.	I understand that my personal data (name, address, telephone number) will be used for the purposes of posting study inhalers to my home address and for contacting me. I give permission for this information to be stored by responsible people at my local NHS Trust and for them to be transferred to Royal Mail, or any alternative postal or courier service used by my local hospital, so that my replacement inhalers can be posted to my home address.	
5.	I understand that information about me that is relevant to this study will leave the Trust and be sent securely to Newcastle University. This includes my date of birth, gender and ethnicity which will be stored on the study database. My NHS number will also be collected and stored separately in order to collect data about my long-term health, and could be accessed for up to three years after my enrollment into this study. I understand that my data will be stored securely and managed confidentially as part of this study.	
6.	I understand that any personal information collected about me for the study will be kept confidential and not be made public. I understand that data from the study will be	

For peer review only

For peer review only - http://bmjopen.bmj.com/site/about/guidelines.xhtml

	published in medical journals, at research meetings and shared with other researchers, including researchers potentially outside the United Kingdom (UK) in the European Economic Area (EEA). I understand that data from the study will be de-identified and that I will not be directly identified in the published results.	
7.	I understand that de-identified data from the study will be shared with GlaxoSmithKline plc (GSK), including the following personal information:<br>• Age<br>• Sex at birth (female/male)<br>• Information about my bronchiectasis disease<br>• Ethnicity<br>• Study identification number<br>I understand that I will not be directly identified in any data shared with GSK.	
8.	I consent to study data being transferred to GSK plc. I understand that GSK is an international company and that data may be transferred outside of the United Kingdom (UK).	
9.	I understand that the information held and maintained by:<br>• Bronchiectasis Databases (including BronchUK [The United Kingdom Bronchiectasis Registry] and EMBARC [European Multicentre Bronchiectasis Audit and Research Collaboration], if you have previously consented to take part in these projects)<br>• NHS Digital (including Hospital Episodes Statistics [HES]-not applicable for Scottish sites)<br>• Central UK NHS bodies (including the Office for National Statistics [ONS]-not applicable for Scottish sites)<br>may be used to provide information about my health status to the research team. I consent to the research team requesting information about my health from routine sources, including Bronchiectasis Databases, NHS Digital and ONS (if applicable) as needed, including up to one year after my participation in the study finishes, and to reviewing my medical records to compare this information.	
10.	I understand that the information collected about me will be used to support other research in the future, and may be shared anonymously with other researcher projects and researchers.	
11.	I understand that parts of my medical records and data collected during the study may be looked at by responsible people. I give my permission for these people to have	

For peer review only

For peer review only - http://bmjopen.bmj.com/site/about/guidelines.xhtml

	access to my medical records.<br>This includes people from Newcastle University, study Sponsor, regulatory authorities and local NHS Trust where it is relevant to my taking part in research.	
12.	I understand that the information provided in this study is being managed by the Newcastle Clinical Trials Unit, which is part of Newcastle University.	
13.	I agree to my General Practitioner being involved in the study, including any necessary exchange of information about me between my GP and the research team	
14.	I agree to the information provided and this signed consent form being stored for 5 years after the end of the study.	
15.	I agree to take part in the above study.	

For participants of childbearing potential:		
16.	I understand that I will have to use an effective form of contraception as outlined in the Participant Information Sheet, if sexually active.	
17.	Females only: I understand that if I am of childbearing potential, I will need to have a urine pregnancy test to ensure that I am not pregnant. I understand that this is for safety reasons.	

Name of Participant	Date	Signature
Name of Person taking consent	Date	Signature

When completed: File 1 copy in the investigator site file (original); Provide 1 copy for the study participant; File 1 copy in the participant's medical notes.

For peer review only - http://bmjopen.bmj.com/site/about/guidelines.xhtml

Reporting checklist for protocol of a clinical trial.

Based on the SPIRIT guidelines.

Instructions to authors

Complete this checklist by entering the page numbers from your manuscript where readers will find each of the items listed below.

Your article may not currently address all the items on the checklist. Please modify your text to include the missing information. If you are certain that an item does not apply, please write "n/a" and provide a short explanation.

Upload your completed checklist as an extra file when you submit to a journal.

In your methods section, say that you used the SPIRITreporting guidelines, and cite them as:

Chan A-W, Tetzlaff JM, Gøtzsche PC, Altman DG, Mann H, Berlin J, Dickersin K, Hróbjartsson A, Schulz KF, Parulekar WR, Krleža-Jerić K, Laupacis A, Moher D. SPIRIT 2013 Explanation and Elaboration: Guidance for protocols of clinical trials. BMJ. 2013;346:e7586

		Reporting Item	Page Number
**Administrative information**			
Title	#1	Descriptive title identifying the study design, population, interventions, and, if applicable, trial acronym	1
Trial registration	#2a	Trial identifier and registry name. If not yet registered, name of intended registry	2
Trial registration: data set	#2b	All items from the World Health Organization Trial Registration Data Set	1-22
Protocol version	#3	Date and version identifier	19
Funding	#4	Sources and types of financial, material, and other support	21
Roles and responsibilities: contributorship	#5a	Names, affiliations, and roles of protocol contributors	21

For peer review only

Roles and responsibilities: sponsor contact information	#5b	Name and contact information for the trial sponsor	4
Roles and responsibilities: sponsor and funder	#5c	Role of study sponsor and funders, if any, in study design; collection, management, analysis, and interpretation of data; writing of the report; and the decision to submit the report for publication, including whether they will have ultimate authority over any of these activities	4
Roles and responsibilities: committees	#5d	Composition, roles, and responsibilities of the coordinating centre, steering committee, endpoint adjudication committee, data management team, and other individuals or groups overseeing the trial, if applicable (see Item 21a for data monitoring committee)	4 - 5
**Introduction**			
Background and rationale	#6a	Description of research question and justification for undertaking the trial, including summary of relevant studies (published and unpublished) examining benefits and harms for each intervention	3 - 4
Background and rationale: choice of comparators	#6b	Explanation for choice of comparators	3 - 5
Objectives	#7	Specific objectives or hypotheses	6 - 9
Trial design	#8	Description of trial design including type of trial (eg, parallel group, crossover, factorial, single group), allocation ratio, and framework (eg, superiority, equivalence, non-inferiority, exploratory)	4
**Methods: Participants, interventions, and outcomes**			

For peer review only

Study setting	#9	Description of study settings (eg, community clinic, academic hospital) and list of countries where data will be collected. Reference to where list of study sites can be obtained	4
Eligibility criteria	#10	Inclusion and exclusion criteria for participants. If applicable, eligibility criteria for study centres and individuals who will perform the interventions (eg, surgeons, psychotherapists)	5 - 6
Interventions: description	#11a	Interventions for each group with sufficient detail to allow replication, including how and when they will be administered	10 – 11
Interventions: modifications	#11b	Criteria for discontinuing or modifying allocated interventions for a given trial participant (eg, drug dose change in response to harms, participant request, or improving / worsening disease)	10, 13
Interventions: adherance	#11c	Strategies to improve adherence to intervention protocols, and any procedures for monitoring adherence (eg, drug tablet return; laboratory tests)	11
Interventions: concomitant care	#11d	Relevant concomitant care and interventions that are permitted or prohibited during the trial	N/A – none specified by trial
Outcomes	#12	Primary, secondary, and other outcomes, including the specific measurement variable (eg, systolic blood pressure), analysis metric (eg, change from baseline, final value, time to event), method of aggregation (eg, median, proportion), and time point for each outcome. Explanation of the clinical relevance of chosen efficacy and harm outcomes is strongly recommended	6 - 9
Participant timeline	#13	Time schedule of enrolment, interventions (including any run-ins and washouts), assessments, and visits for participants. A schematic diagram is highly recommended (see Figure)	11 - 12

For peer review only

Sample size	#14	Estimated number of participants needed to achieve study objectives and how it was determined, including clinical and statistical assumptions supporting any sample size calculations	17 - 18
Recruitment	#15	Strategies for achieving adequate participant enrolment to reach target sample size	9, 18
**Methods: Assignment of interventions (for controlled trials)**			
Allocation: sequence generation	#16a	Method of generating the allocation sequence (eg, computer-generated random numbers), and list of any factors for stratification. To reduce predictability of a random sequence, details of any planned restriction (eg, blocking) should be provided in a separate document that is unavailable to those who enrol participants or assign interventions	10
Allocation concealment mechanism	#16b	Mechanism of implementing the allocation sequence (eg, central telephone; sequentially numbered, opaque, sealed envelopes), describing any steps to conceal the sequence until interventions are assigned	10
Allocation: implementation	#16c	Who will generate the allocation sequence, who will enrol participants, and who will assign participants to interventions	10
Blinding (masking)	#17a	Who will be blinded after assignment to interventions (eg, trial participants, care providers, outcome assessors, data analysts), and how	10 - 11
Blinding (masking): emergency unblinding	#17b	If blinded, circumstances under which unblinding is permissible, and procedure for revealing a participant's allocated intervention during the trial	13
**Methods: Data collection,**			

For peer review only

**management, and analysis**			
Data collection plan	#18a	Plans for assessment and collection of outcome, baseline, and other trial data, including any related processes to promote data quality (eg, duplicate measurements, training of assessors) and a description of study instruments (eg, questionnaires, laboratory tests) along with their reliability and validity, if known. Reference to where data collection forms can be found, if not in the protocol	13 - 14
Data collection plan: retention	#18b	Plans to promote participant retention and complete follow-up, including list of any outcome data to be collected for participants who discontinue or deviate from intervention protocols	13
Data management	#19	Plans for data entry, coding, security, and storage, including any related processes to promote data quality (eg, double data entry; range checks for data values). Reference to where details of data management procedures can be found, if not in the protocol	13 – 14
Statistics: outcomes	#20a	Statistical methods for analysing primary and secondary outcomes. Reference to where other details of the statistical analysis plan can be found, if not in the protocol	14
Statistics: additional analyses	#20b	Methods for any additional analyses (eg, subgroup and adjusted analyses)	15 - 16
Statistics: analysis population and missing data	#20c	Definition of analysis population relating to protocol non-adherence (eg, as randomised analysis), and any statistical methods to handle missing data (eg, multiple imputation)	14 - 15
**Methods: Monitoring**			
Data monitoring: formal committee	#21a	Composition of data monitoring committee (DMC); summary of its role and reporting	5

For peer review only

		structure; statement of whether it is independent from the sponsor and competing interests; and reference to where further details about its charter can be found, if not in the protocol. Alternatively, an explanation of why a DMC is not needed	
Data monitoring: interim analysis	#21b	Description of any interim analyses and stopping guidelines, including who will have access to these interim results and make the final decision to terminate the trial	No interim analysis
Harms	#22	Plans for collecting, assessing, reporting, and managing solicited and spontaneously reported adverse events and other unintended effects of trial interventions or trial conduct	14
Auditing	#23	Frequency and procedures for auditing trial conduct, if any, and whether the process will be independent from investigators and the sponsor	13
**Ethics and dissemination**			
Research ethics approval	#24	Plans for seeking research ethics committee / institutional review board (REC / IRB) approval	19
Protocol amendments	#25	Plans for communicating important protocol modifications (eg, changes to eligibility criteria, outcomes, analyses) to relevant parties (eg, investigators, REC / IRBs, trial participants, trial registries, journals, regulators)	18 - 19
Consent or assent	#26a	Who will obtain informed consent or assent from potential trial participants or authorised surrogates, and how (see Item 32)	9
Consent or assent: ancillary studies	#26b	Additional consent provisions for collection and use of participant data and biological specimens in ancillary studies, if applicable	N/A – no ancillary studies
Confidentiality	#27	How personal information about potential and enrolled participants will be collected, shared, and	13 - 14

For peer review only

		maintained in order to protect confidentiality before, during, and after the trial	
Declaration of interests	#28	Financial and other competing interests for principal investigators for the overall trial and each study site	22
Data access	#29	Statement of who will have access to the final trial dataset, and disclosure of contractual agreements that limit such access for investigators	13 - 14
Ancillary and post trial care	#30	Provisions, if any, for ancillary and post-trial care, and for compensation to those who suffer harm from trial participation	
Dissemination policy: trial results	#31a	Plans for investigators and sponsor to communicate trial results to participants, healthcare professionals, the public, and other relevant groups (eg, via publication, reporting in results databases, or other data sharing arrangements), including any publication restrictions	19
Dissemination policy: authorship	#31b	Authorship eligibility guidelines and any intended use of professional writers	N/A
Dissemination policy: reproducible research	#31c	Plans, if any, for granting public access to the full protocol, participant-level dataset, and statistical code	N/A
**Appendices**			
Informed consent materials	#32	Model consent form and other related documentation given to participants and authorised surrogates	Supplementary
Biological specimens	#33	Plans for collection, laboratory evaluation, and storage of biological specimens for genetic or molecular analysis in the current trial and for future use in ancillary studies, if applicable	N/A – no biological specimens

None The SPIRIT Explanation and Elaboration paper is distributed under the terms of the Creative Commons Attribution License CC-BY-NC. This checklist can be completed online using

For peer review only

https://www.goodreports.org/, a tool made by the EQUATOR Network in collaboration with Penelope.ai

For peer review only
